# Intra- *versus* intermolecular electron transfer in radical nucleophilic aromatic substitution of dihalo(hetero)arenes – a tool for estimating π-conjugation in aromatic systems†
†Electronic supplementary information (ESI) available: Experimental details and procedures, ^1^H and ^13^C NMR data, GC traces and mass spectra. CCDC 1526301 and 1526302. For ESI and crystallographic data in CIF or other electronic format see DOI: 10.1039/c7sc00100b



**DOI:** 10.1039/c7sc00100b

**Published:** 2017-02-23

**Authors:** B. Janhsen, C. G. Daniliuc, A. Studer

**Affiliations:** a Organisch Chemisches Institut , Westfälische Wilhelms-Universität Münster , Corrensstraße 40 , 48149 Münster , Germany . Email: studer@uni-muenster.de

## Abstract

In this paper, the application of the double radical nucleophilic aromatic substitution (S_RN_1) in various dihalogenated, mostly diiodinated, π-conjugated systems as a tool for qualitatively estimating their π-conjugation is described. This approach uses electron delocalisation as a measure of π-conjugation. Electron injection into the π-system is achieved *via* reaction of an intermediate aryl radical, itself generated from a dihalogenated π-system *via* SET-reduction of the C–I bond and subsequent reaction with a thiolate anion. The generated arene radical anion can then further react with the second aryl-halogen moiety within the π-system *via* an intramolecular electron transfer process. The efficiency of this intramolecular electron transfer is related to the π-conjugation of the radical anion. If the π-conjugation within the aromatic unit is weak, the arene radical anion reacts *via* an intermolecular ET with the starting dihalide. The intramolecular ET process delivers a product of a double S_RN_1 substitution whereas the intermolecular ET pathway provides a product of a mono- S_RN_1 substitution. By simple product analysis of mono- *versus* double substitution, π-conjugation can be qualitatively evaluated. This mechanistic tool is applied to various dihalogenated π-conjugated systems and the results are discussed within the context of π-conjugation. The conjugation mode within the π-system and the length of the aromatic system are varied, and the effect of relative positioning of the two halides within small π-systems is also addressed.

## Abstract 2

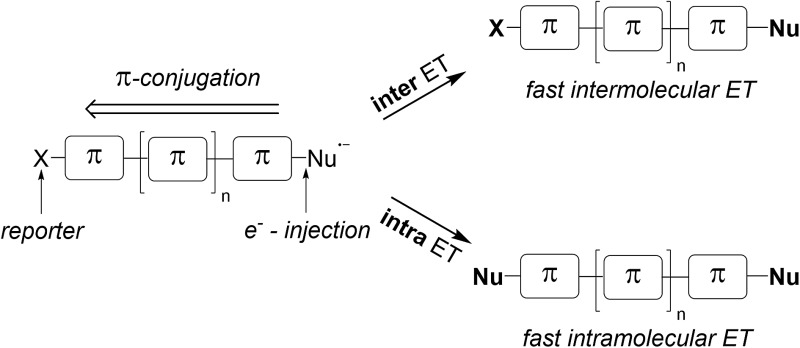
In this paper, the application of the double radical nucleophilic aromatic substitution (S_RN_1) in various dihalogenated, mostly diiodinated, π-conjugated systems as a tool for qualitatively estimating their π-conjugation is described.

## Introduction

**Scheme 1:**
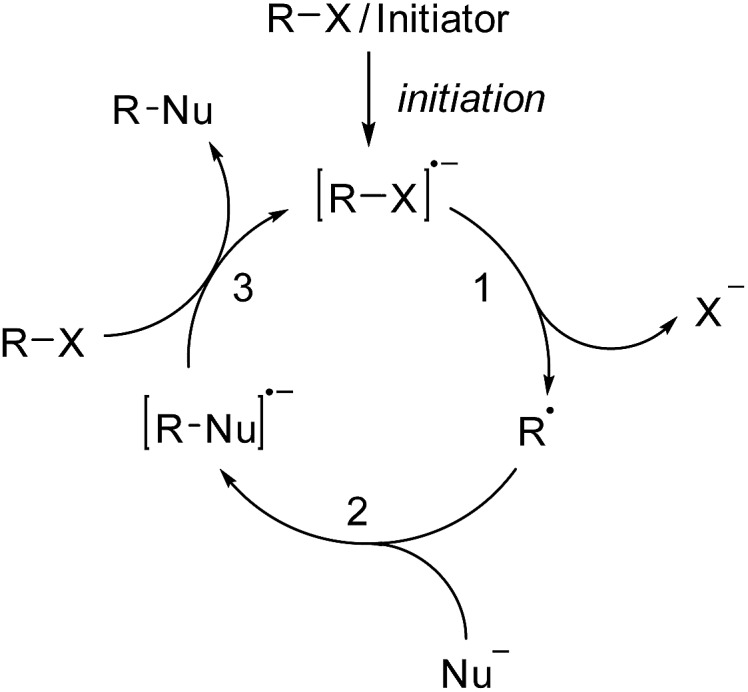
The general mechanism of the S_RN_1 substitution.

Over the past forty years, radical nucleophilic aromatic substitution (S_RN_1) has been found to be a highly valuable reaction in organic synthesis.^1^ This process has been applied to the synthesis of natural products^2^ and also in neighboring disciplines for the preparation of oligomers^3^ and polymers.^4^ Its radical chain mechanism was first proposed by Kornblum^5^ and Russell^6^ for activated aliphatic and aromatic substrates. Later Bunnett^7^ widened the scope by also performing the substitution on unactivated aromatic systems. As for any radical chain reaction, the S_RN_1 reaction needs to be initiated. In many cases light has been used to initiate the chain *via* an electron transfer (ET) process from an initiator to the substrate halide.^1^ The resulting radical anion then fragments to the corresponding C-radical and an anion (step 1, Scheme 1), which is usually a halide.^1^ In some cases the radical anion is not an intermediate (ET and halide fragmentation are concerted in a so-called dissociative ET).^8^ The C-radical is then attacked by an anionic nucleophile (step 2) in order to generate a radical anion, which in turn acts as an electron transfer reagent (reductant). Single electron transfer (SET) to the starting substrate halide eventually provides the substitution product along with the substrate halide radical anion, thereby sustaining the chain (step 3). C–C bond formation has been achieved with enolates as nucleophiles, but in most cases the S_RN_1 reaction has been applied to C-heteroatom bond formation with S-, P-, Sn-, As-, Sb-, Se-, and Te-centered nucleophiles.^1^

**Scheme 2:**
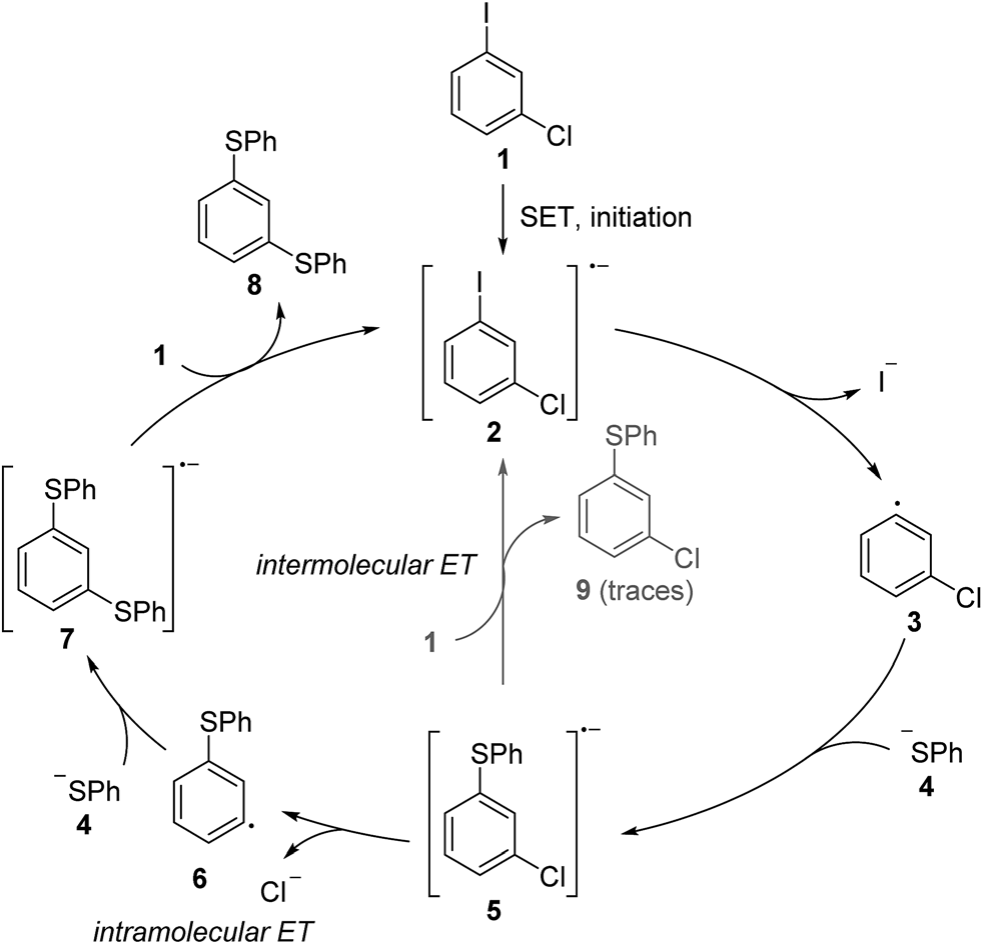
Bunnett and Creary’s experiment.

Since the S_RN_1 reaction belongs to an electron-catalyzed process,^9^ the mechanism depicted in Scheme 1 is drawn accordingly as a catalytic cycle.^10^ The suggested mechanism of the S_RN_1 substitution was further supported by Bunnett and Creary using an elegant experiment.^11^ They showed that the reaction of *meta*-chloro-iodobenzene (**1**) with PhSH in ammonia under S_RN_1 conditions provides the bisthioether **8** as a major product, along with traces of the monosubstituted chlorothioether **9** (Scheme 2). It was also shown that **9** is a sluggish substrate for an S_RN_1 reaction and therefore **8** cannot be derived from **9** under the applied conditions. The following mechanism was suggested based on these experimental observations. ET initiation generates the radical anion **2**, which chemoselectively fragments the iodide anion to give the aryl radical **3**. Addition of the thiolate to the aryl radical affords the radical anion adduct **5**. This intermediate can now either react *via* an intramolecular ET (from the π* of **5** to the σ*_(C–Cl)_)^12^ and subsequent chloride fragmentation to the aryl radical **6** or *via* an intermolecular ET transfer (from the π* of **5** to the π* of substrate **1**) to the monosubstitution product **9**. The aryl radical **6** then reacts with the thiolate *via* **7** to the experimentally observed double substitution product **8**. Due to the fact that double substitution occurred nearly exclusively, the intramolecular ET in the radical anion **5** is far faster than the intermolecular ET.‡
‡This is not surprising considering the fast kinetics of halide fragmentation in halogen substituted arene radical anions, see ref. 25 and references cited therein. This result also provided indirect proof for the existence of the radical anion **5** as an intermediate in the chain reaction and strongly supported the general mechanism of the S_RN_1 reaction that is depicted in Scheme 1.

Dihalo-substituted alkanes including dihalocyclopropanes,^13^ dihaloadamantanes,^14^ and dihalobicyclo[2.2.2]octanes^15^ were also studied with respect to the S_RN_1 double substitution. For aromatic systems, *ortho*-,^1,16^ *meta*-,^11,17^ and *para*-dihalobenzenes^18^ and 1,8-diiodonaphthalene^19^ were investigated along these lines.

However, more complex aromatic structures bearing two halide substituents in extended π-systems have not been studied to date in the context of a double S_RN_1 substitution. We assumed this process to be a simple and efficient tool for analyzing the relative ratio of intramolecular *versus* intermolecular electron transfer in π-conjugated radical anions. Importantly, this ratio should offer a measure for evaluating the π-conjugation in an aromatic system, which is of course an important parameter in various fields of modern materials science.

**Scheme 3:**
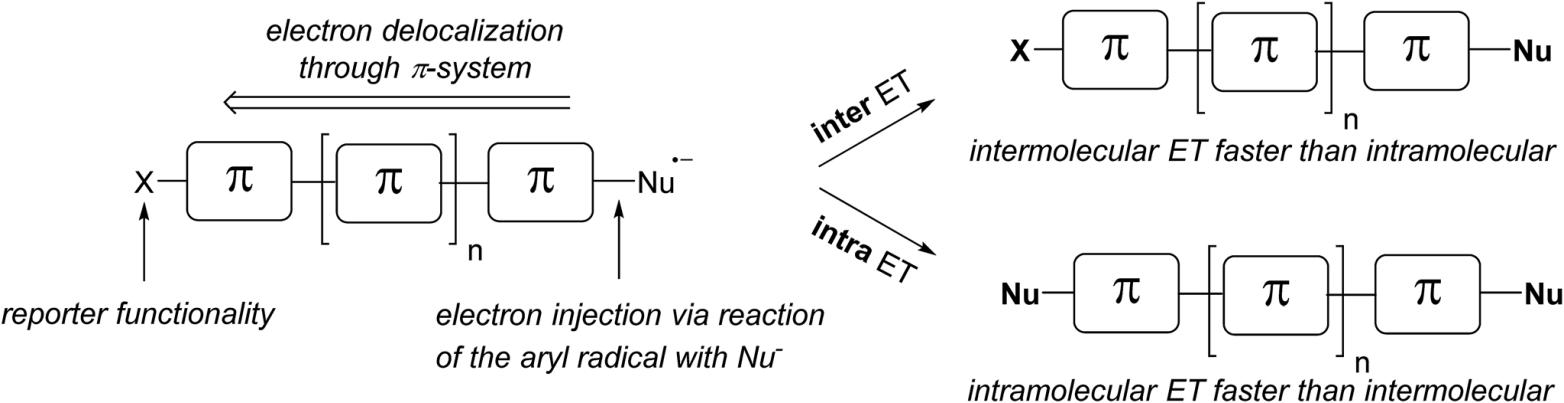
Inter- *versus* intramolecular ET in π-radical anions – a tool for estimating the π-conjugation of an aromatic system.

In this work we introduce the double S_RN_1 substitution in dihalo(hetero)arenes as a novel qualitative tool for the analysis of the relative intra- *versus* intermolecular ET efficiency in various π-radical anions. Electron injection into the π-system is achieved *via* the reaction of an intermediate aryl radical with an anionic nucleophile. Since the aryl radical is generated at a specific site in the molecule, the electron injection occurs site specifically at the σ* orbital of the newly formed C–S bond. The ratio of double *versus* single S_RN_1 substitution, readily determined by product analysis at low conversion, will allow the estimation of the relative inter- *versus* intramolecular ET efficiency (Scheme 3). Importantly, the intramolecular ET efficiency should be related to the π-conjugation of the aromatic system. The conjugation mode within the π-system and the length of the aromatic system will be varied, and the effect of the relative positioning of the two halides within small π-systems will also be addressed.

## Experimental

### Materials and methods

All reactions involving air or moisture sensitive reagents were carried out in flame-dried glassware under an atmosphere of argon. All solvents and reagents were purified according to standard procedures or were used as-received from Sigma Aldrich, Acros, Alfa Aesar, or TCI Europe. Mass spectra were recorded on a Finnigan MAT 4200S, a Bruker Daltonics Micro Tof, and a Waters-Micromass Quattro LCZ (ESI) and peaks are given in *m*/*z* (% of basis peak). ESI-MS (*m*/*z*) and HRMS (*m*/*z*) were performed using a Bruker MicroTof (loop injection; resolution: 10 000), a LTQ Orbitrap XL (nanospray inlet, 1.1 kV, resolution: 30 000), and an Autoflex Speed TOF-MS (Bruker Daltonics). MALDI spectra were recorded with an Autoflex Speed TOF-MS (Bruker Daltonics) in linear mode. GC-MS (EI, 70 eV) was performed on a combined setup of an Agilent 6890N chromatograph equipped with a HP-5 column, using helium (∼1 bar) as the carrier gas, and a Waters Micromass Quattro Micro spectrometer. Gas chromatography (GC-FID) was performed on an Agilent 7890A chromatograph, which was equipped with a HP-5 column (30 m × 0.32 mm, film thickness 0.25 μm), using H_2_ (∼1 bar) as the carrier gas.

### Synthetic procedures

The synthesis of all substrates employed in this work is discussed in detail in the ESI†.

#### General procedure 1 for the substitution reaction initiated with UV-light

4-Methylthiophenol (31 mg, 0.25 mmol, 5.0 equiv.) was dissolved in MeCN (1 mL) and NaH (15 mg, 0.40 mmol, 8.0 equiv.) was added. The resulting mixture was stirred for 1 h and then the dihalide (0.05 mmol, 1 equiv.) was added. The reaction mixture was subjected to UV light (365 nm, 30 W), for times ranging from 5 min to 8 h, obtaining conversions mostly <50% (reaction times and conversions for each individual experiment are given in the ESI†). An aliquot was taken, diluted with EtOAc and washed with water. The solvent was removed *in vacuo* and analyzed using GC-FID/GC-MS or MS.

#### General procedure 2 for the substitution reaction initiated thermally

4-Methylthiophenol (31 mg, 0.25 mmol, 5.0 equiv.) was dissolved in DMSO (1 mL) and NaH (15 mg, 0.40 mmol, 8.0 equiv.) was added. The resulting mixture was stirred for 1 h and then the dihalide (0.05 mmol, 1 equiv.) was added. The reaction mixture was heated to 120 °C for 6–16 h obtaining conversions mostly <50% (reaction times and conversions for each individual experiment are given in the ESI†). An aliquot was taken, diluted with EtOAc and washed with water. The solvent was removed *in vacuo* and analyzed using MALDI spectrometry.

## Results and discussion

Since the iodide anion is a highly efficient leaving group in S_RN_1 chemistry we decided to mainly focus on diiodoarenes as substrates. In order to keep the product analysis as simple as possible, we took symmetrical dihaloarenes as coupling partners. To ensure that the disubstitution product does not derive from the monosubstitution compound *via* a renewed S_RN_1 process, the reactions were stopped at low conversion (mostly <50%). NaSC_6_H_4_CH_3_, which is readily generated by deprotonation of *para*-methylthiophenol with NaH, was chosen as a nucleophile and reactions were conducted in acetonitrile under UV irradiation (365 nm) at room temperature, unless otherwise noted. The formation of mono- and/or double S_RN_1 substitution products was analyzed using gas chromatography or mass spectrometry (see ESI†). The reactions were generally clean and, along with the mono- and/or doubly substituted S_RN_1 products, unreacted starting material and disulfide derived from oxidation of the thiolate during analysis were identified. In most cases, the mono-reduced (hydrodehalogenated) derivatives of the substrate were also formed as typical side-products in photoinitiated S_RN_1 reactions.^1^

**Scheme 4:**
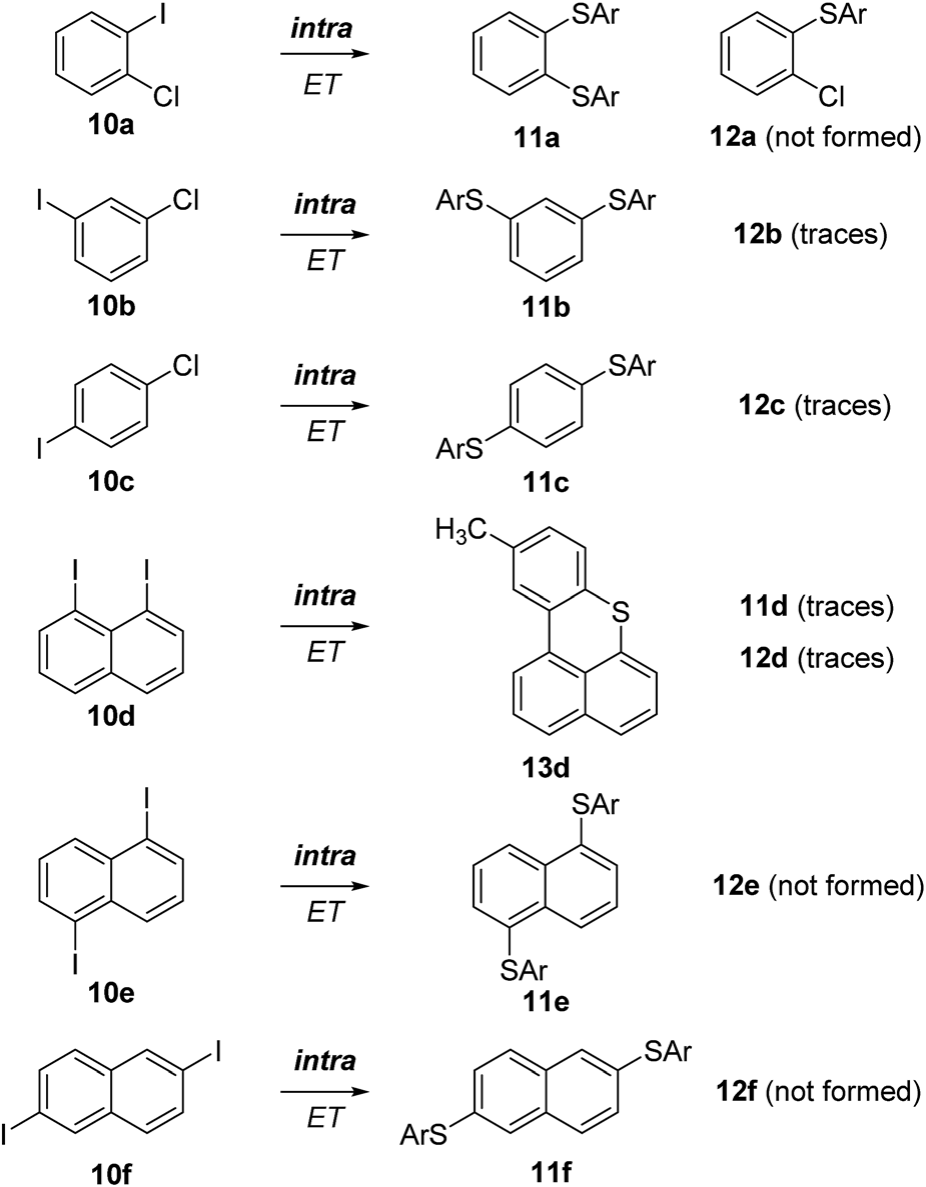
S_RN_1 substitution on various dihalobenzenes and diiodonaphthalenes. The **11**-series, including **13**, represents the double substitution products derived from an intramolecular ET.

The simple dihalobenzenes **10a–c** were investigated first (Scheme 4). All of them showed the corresponding double substitution products **11a**, **11b**, and **11c** derived from intramolecular ET and only traces of the corresponding monosubstituted products **12b** and **12c** were identified. These results are in agreement with literature reports.^11,18^

In the naphthalene series, the 1,8-diiodo derivative **10d** underwent S_RN_1 substitution followed by an intramolecular homolytic aromatic substitution to eventually give **13d**.^19,20^ This product results from an intramolecular ET and only traces of the mono S_RN_1 product **12d**, which is formed *via* an intermolecular ET, were identified in the reaction mixture. A small amount of the double substitution product **11d** was formed along with the main product (**13d**). The 1,5-diiodonaphthalene (**10e**) afforded only the disubstitution product **11e** by an intramolecular ET and monosubstituted **12e** was not formed. The 2,6-isomer **10f** provided selectively the disubstitution product **11f** *via* an efficient intramolecular ET in the intermediate radical anion. Hence, as expected, the intramolecular ET in naphthalene radical anions is highly efficient.

**Scheme 5:**
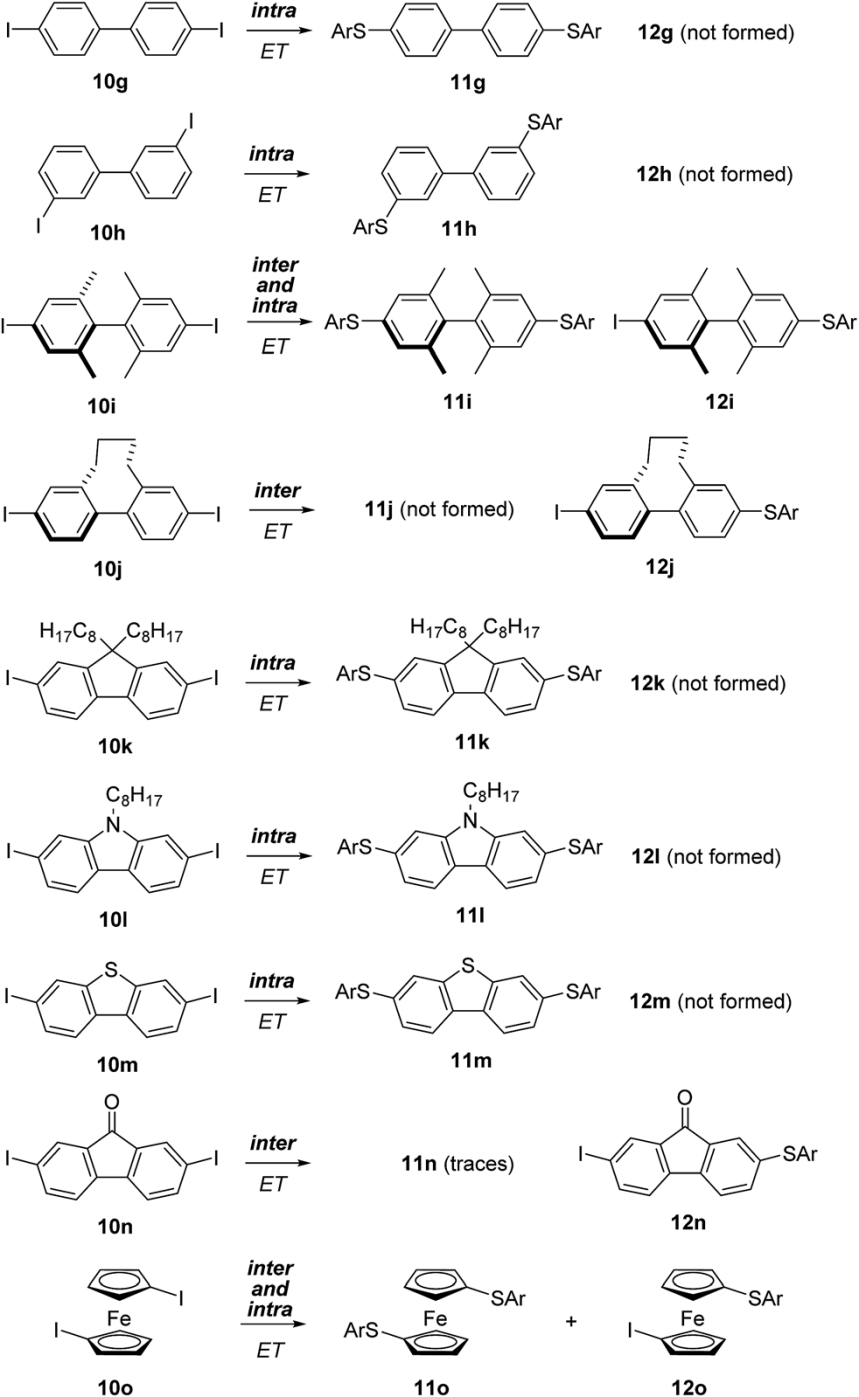
Double S_RN_1 substitution on various diiodo-biaryls.

We then decided to apply the π-conjugation test to more complex aromatic structures, and started to investigate biphenyl derivatives. Biphenyl is the smallest entity containing two adjacent aromatic rings connected *via* a π-bond and various derivatives are synthetically readily accessible. The 4,4′-diiodo biphenyl **10g** and the 3,3′-diiodo congener **10h** exclusively provided the double S_RN_1 substitution products **11g** and **11h** *via* an intramolecular ET process (Scheme 5). It was shown in previous studies that the conductance of a biphenyl-derived molecular conductor is correlated with the torsion angle along the aryl–aryl bond.^21^ For 4,4′- and 3,3′-disubstituted biaryls, a conformation with both arene rings oriented in-plane (torsion angle = 0°) should be readily accessible. For intramolecular ET in biaryl radical anions, a planar conformation with a torsion angle of 0° would be an ideal explanation for the exclusive formation of the double substitution products **11g** and **11h** in the transformations of **10g** and **10h**.

**Fig. 1:**
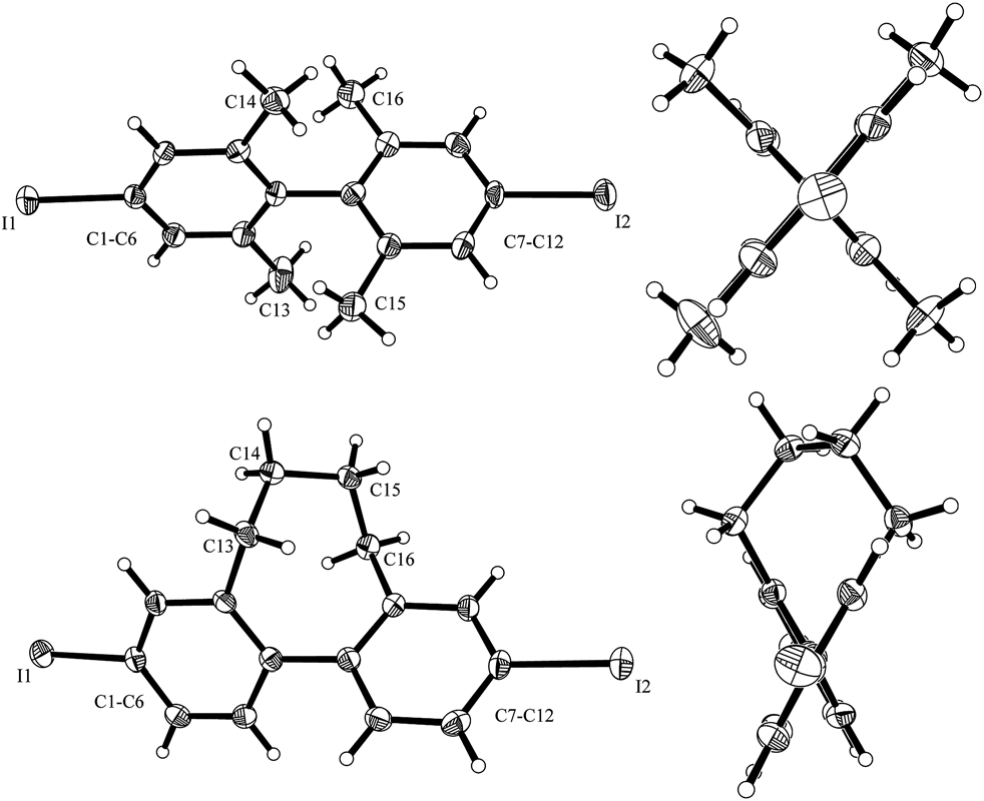
X-ray structure of **10i** (top) and **10j** (bottom) with the side view (left) and view along the biphenyl system (right; thermal ellipsoids are shown with 30% probability). CCDC numbers for **10i**: ; 1526301 and **10j**: ; 1526302.

To further address that important issue, we prepared *para*-diiodo biaryls **10i** and **10j** where conformation around the biaryl axis is controlled by the *ortho*-substitution pattern. The structures of these diiodides were characterized using X-ray crystal structure analysis (Fig. 1). The torsion angle for **10i** is 83.0° and for **10j** it is 58.1°. Despite the large torsion angle in **10i**, both mono- and bis-substitution products **11i** and **12i** were formed in the S_RN_1 substitution. However, in the case of **10j** only the mono-substitution product was identified, showing that the intramolecular ET process is slower than the intermolecular ET process in such a conformationally rigid biaryl radical anion with a fixed torsion angle that does not allow electronic communication between the two arenes. In the case of the radical anion derived from **10i**, despite the larger torsion angle in the crystalline state, intramolecular ET is feasible due to rotational flexibility under the reaction conditions in solution, allowing the two arene moieties in their radical anionic state to interact. The ratio of bis- to monosubstitution (**11i** : **12i**) is dependent on the substrate concentration (see ESI†). The higher the concentration, the more product **12i** derived from an intermolecular ET observed, and the concentration linearly correlates with the **11i** : **12i** ratio.

Based on these results we assumed that the 2,7-diiodo-9,9′-dioctyl fluorene (**10k**) with a fixed planar biaryl structural motif would be a good substrate for intramolecular ET. Indeed, selective formation of the double S_RN_1 product **11k** was obtained in the experiment. Along these lines, the carbazole **10l** and benzothiophene **10m** afforded the corresponding double S_RN_1 substitution products **11l** and **11m** with complete selectivity. The additional heteroatom in these conformationally fixed biaryl radical anions do not influence the intramolecular ET to a large extent. In both cases the intramolecular ET is faster than the intermolecular process.

However, incorporating a carbonyl group as a biaryl bridging moiety changes the reactivity towards formation of, nearly exclusively, the monosubstitution product **12n**. In this substrate the carbonyl group likely stabilizes the intermediate radical anion so that fragmentation of the iodide is slowed down, allowing for intermolecular ET to occur. As an additional substrate, we tested the diiodoferrocene **10o** and found both the mono- (**12o**) and disubstitution (**11o**) products. Hence, electronic communication between the two arenes in the radical anion *via* the Fe-atom is possible but competes with the intermolecular ET.

**Scheme 6:**
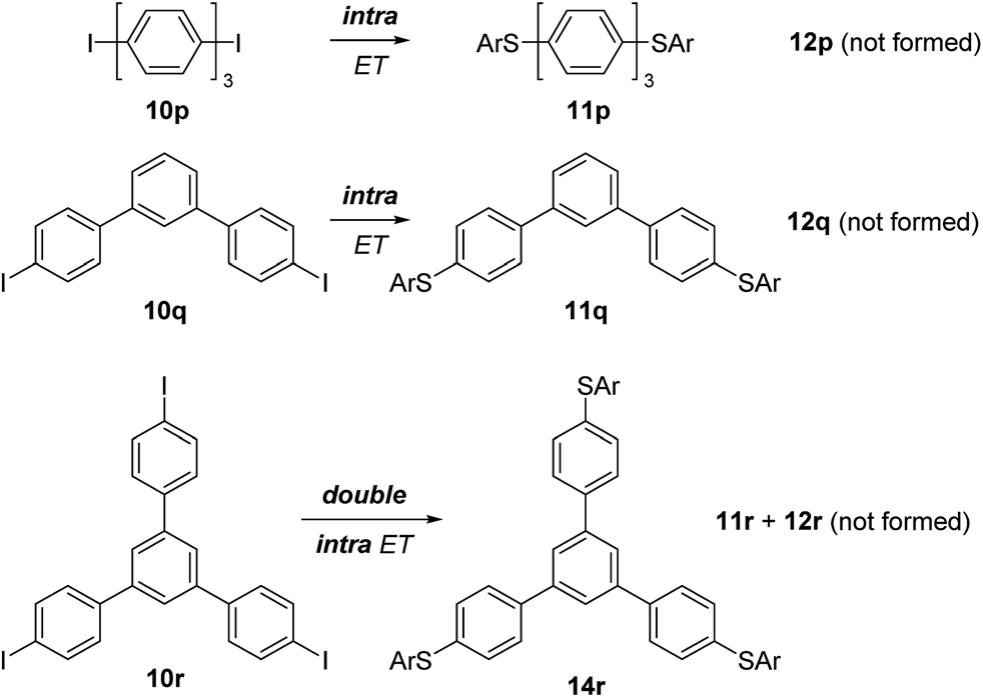
Double S_RN_1 substitution in triaryl systems and triple S_RN_1 substitution in 1,3,5-*tris*(*p*-iodophenyl)benzene (**10r**).

Our next efforts concentrated on the intra/inter electron transfer efficiency in triphenyl systems. 4,4′′-Diiodo-*p*-terphenyl (**10p**) was employed and efficient intramolecular ET in the corresponding intermediate radical anion was observed, leading selectively to **11p** as the only substitution product. *Meta*-connected terphenyl **10q** also provided exclusively doubly substituted **11q** indicating that the connectivity pattern of the aryl moieties does not produce an observable effect. The triiodide **10r** yielded the trisubstitution product **14r** as the only substitution product. This result shows that two consecutive intramolecular ETs in this substrate are faster than a single intermolecular ET from this molecule. To our knowledge, this is the first report on a triple substitution in S_RN_1-type chemistry (Scheme 6).

**Scheme 7:**
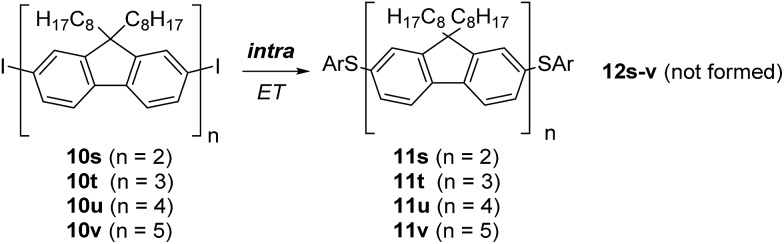
Double S_RN_1 substitution in extended π-systems.

We continued the studies by looking at oligofluorenes, and prepared the dimer **10s**, trimer **10t**, tetramer **10u**, and pentamer **10v** (see ESI†).§
§The starting diiodides contained the monoiodo-derivatives and the doubly hydrodeiodinated oligofluorenes that could not be separated, see ESI.† However, these impurities influence neither the reaction nor the mass spectrometry analysis. The conjugation length in **10s** corresponds to a *para*-quaterphenyl with pairs of phenylene moieties fixed. Accordingly, **10t** would be related to a conformationally partly fixed *para*-sexiphenyl, **10u** to a *para*-octiphenyl, and **10v** to a *para*-deciphenyl. S_RN_1 substitutions were conducted in DMSO with thermal initiation at 110 °C due to the low solubility of these substrates at room temperature. In place of GC-MS analysis, MALDI-MS was employed for reaction analysis (see ESI†). Reaction with the dimer **10s** provided only the disubstitution product **11s**, showing that intramolecular ET *via* the four phenylene moieties is efficient and intermolecular ET cannot compete. Accordingly, **12s** was not identified in the reaction mixture. Even for the pentamer **10v** the intramolecular ET was highly efficient and only the double S_RN_1 substitution product **11v** was formed at low conversion. These results illustrate the high efficiency of the intramolecular ET in oligofluorenes and underline the conductive properties of such π-radical anions (Scheme 7).^22^

**Scheme 8:**
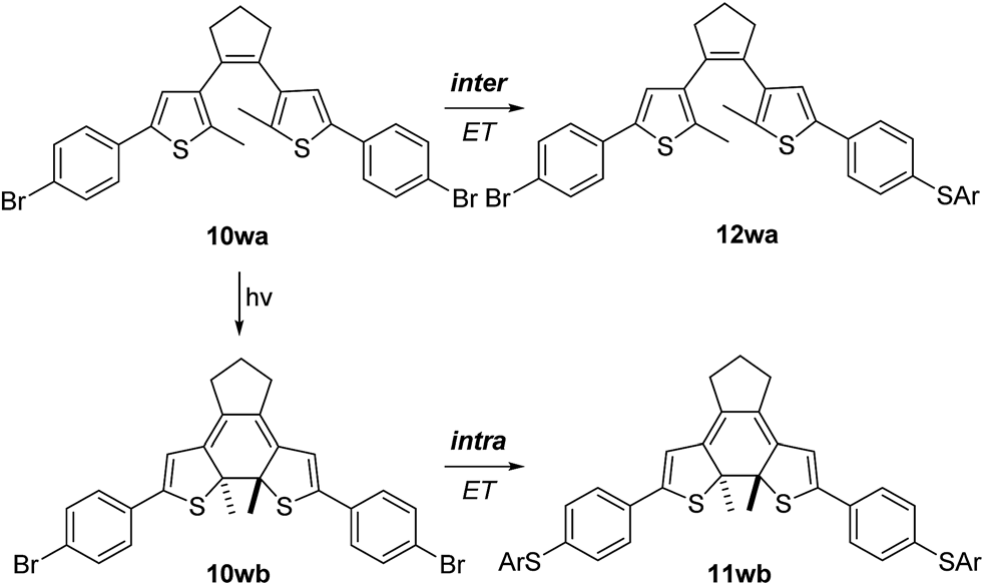
S_RN_1 substitution experiments on the photochemically switchable π-system **10w**.

Finally, we examined the electron-transfer efficiency using the photochemically switchable π-system **10w**. The S_RN_1 test was applied to its open (**10wa**) and closed (**10wb**) form.^23^ We chose the bisbromo compound because the corresponding bisiodide^24^ was not stable under the photochemical conditions used for ring closure. By employing UV light (320 nm) in THF-d8, **10wa** could be readily transformed into **10wb**. S_RN_1 substitution of **10wa** with NaSC_6_H_4_CH_3_ was initiated thermally in MeCN (see ESI†) to prevent light-mediated ring closure during the S_RN_1 reaction, whereas the reaction with **10wb** was initiated by UV light at room temperature to prevent thermal ring opening. The open form **10wa** showed monosubstitution to produce **12wa**.¶
¶The open form **10wa** used contained 10% of the closed form **10wb**. In the S_RN_1 experiment we identified small amounts of the doubly substituted product **11wb** and assumed that this doubly substituted product derived from **10wb** was present as an impurity in the starting material. This was supported by the fact that upon increasing conversion the relative ratio of **12wa**/**11wa** sharply increased, indicating that the closed form reacts exclusively to the monosubstitution product **12wa** (see ESI†). Moreover, we noted **10wb** to be a far more reactive substrate in the S_RN_1 substitution as compared to **10wa**. This is due to the fact that the π-system is not planar and π-conjugation is disrupted, preventing an efficient intramolecular ET. In contrast, for **10wb**, the disubstitution product **11wb** was observed as the exclusive product. In the closed form, the polycyclic core renders the whole molecule planar and rigid, so that efficient intramolecular ET is occurring (Scheme 8).

## Conclusions

We have introduced double S_RN_1 substitution as a tool for estimating the π-conjugation in aromatic systems. In dihalogenated π-systems where good π-conjugation is warranted, the S_RN_1 reaction provides the double substitution product *via* intramolecular electron transfer through the π-system in the corresponding intermediate radical anion. If π-conjugation is low or disturbed, the radical anion reacts *via* intermolecular electron transfer to provide the product of mono S_RN_1 substitution. The product ratio of mono *versus* double S_RN_1 substitution is easily determined using standard analytical tools, allowing for ready qualitative estimation of π-conjugation.

We have shown that, for benzene derivatives, the intramolecular ET in the corresponding radical anions is nearly the exclusive process and that the relative orientation of the two halides is less important. Also, for the π-extended naphthalene systems, almost exclusively intramolecular ET was observed for all systems investigated. In biphenyl derivatives, the torsion angle between the two planes influences the intramolecular ET efficiency. For systems that can reach a planar π-conjugated conformation, only double S_RN_1 substitution *via* intramolecular ET was observed. However, if the two arene moieties in the biaryl radical anions are tilted and conformationally fixed, electron transfer from one arene ring to the other ring is suppressed, and reactions proceed *via* intermolecular ET. This is very similar to electron conductance through biaryls, where torsion angle effects on conductance were also observed.^21^ In π-extended systems represented by oligofluorenes we have shown that the intramolecular electron transfer through the π-system can efficiently compete with the intermolecular ET for a conjugation length of up to 10 phenylene moieties (until *para*-deciphenyl was tested). Moreover, we demonstrated the first example of switchable S_RN_1 reactivity by employing a photochemical switchable substrate.
