# Supplementary material for: Intra- versus intermolecular electron transfer in radical nucleophilic aromatic substitution of dihalo(hetero)arenes – a tool for estimating π-conjugation in aromatic systems
Source: Chem Sci. 2017 Feb 23;8(5):3547–53. doi: 10.1039/c7sc00100b (PMC5436300; doi:10.1039/c7sc00100b)
Supplement: Supplementary file 1 [file SC-008-C7SC00100B-s001.pdf]

Electronic Supplementary Material (ESI) for Chemical Science.
This journal is © The Royal Society of Chemistry 2017

SUPPORTING INFORMATION

Intra- versus Intermolecular Electron Transfer in Radical Nucleophilic Aromatic Substitution of Dihalo(hetero)arenes – a Tool for Estimating Electron Mobility in a π-System

Benjamin Janhsen, Constantin G. Daniliuc and Armido Studer

Organisch-Chemisches Institut, Westfälische Wilhelms-Universität Münster, Corrensstraße 40, 48149 Münster, Germany. E-Mail: studer@uni-muenster.de

Contents

General methods 2
Synthetic procedures 3
References 8
NMR Spectra 9
GC traces and mass spectra analyses of the $S_{RN}1$ substitution reactions 25
Crystallographic data 50

General methods. All reactions involving air- or moisture sensitive reagents were carried out in flame dried glass ware under an atmosphere of argon. THF and diethylether were freshly distilled from potassium under Argon. CH_2Cl_2 was freshly distilled from P_2O_5 under argon. All other solvents and reagents were purified according to standard procedures or were used as received from Sigma Aldrich, Acros, Alfa Aesar or TCI Europe. 1,3,5-Tris(4-iodophenyl)benzene (**10s**),[1] 7-bromo-9,9-dioctyl-9H-fluoren-2-amine,[2] 4,4''-Diiodo-m-terphenyl (**10r**),[3] 2,7-Diiodo-9,9-dioctyl-9*H*-fluorene (**10l**),[4] 2,7-Diiodo-9H-fluoren-9-one (**10o**),[5] 4,4'-Diiodo-2,2',6,6'-tetramethyl-biphenyl (**10j**),[6] 2,2'-Diiodobiphenyl (**10i**),[7] 1,1'-Diiodo-ferrocene (**10p**),[8] 3,7-Diiododibenzo[b,d]thiophene 5,5-dioxide,[9] *N*-octyl-2,7-Diiodocarbazole (**10m**),[10] 4,4'-Diiodo-biphenyl-2,2'-dicarboxylic acid,[11] 7,7''-dibromo-9,9,9',9',9'',9''-hexaoctyl-9H,9'H,9''H-2,2':7',2''-terfluorene[12] and 1,2-Bis(5'-(4''-bromophenyl)-2'-methylthien-3'-yl)-cyclopentene (**10xa**) were synthesized according to known procedures. Diiodo-naphthalenes were synthesized *via Sandmeyer* reaction following a literature procedure.[13] Higher oligomers of fluorene and their brominated analogues were synthesized according to a reported protocol.[12] ^{1}H NMR and ^{13}C NMR spectra were recorded on a Bruker DPX 300 at 300 K. All resonances are reported relative to TMS. Spectra were calibrated relative to the solvent's residual proton and carbon chemical shift. Coupling constants (*J*) are reported in Hz. Infrared spectra were recorded on a Varian Associates FT-IR 3100 equipped with a MKII Golden Gate Single Reflection ATR unit. Recorded IR signals are reported in cm^{-1}. HRMS (ESI): Mass spectra were recorded on a Finnigan MAT 4200S, a Bruker Daltonics Micro Tof, a Waters-Micromass Quatro LCZ (ESI); peaks are given in m/z (% of basis peak). ESI–MS (m/z) and HRMS (m/z) were performed using a Bruker MicroTof (loop injection; resolution: 10,000), a LTQ Orbitrap XL (nanospray inlet, 1.1 KV, resolution: 30,000), and an Autoflex Speed TOF-MS (Bruker Daltonics). MALDI spectra were recorded with an Autoflex Speed TOF-MS (Bruker Daltonics) in linear mode. TLC was performed to monitor reactions using Merck silica gel 60 F-254 plates and detection of compounds was done using UV light. Flash column chromatography (FCC) was performed using Merck silica gel 60 (40-63 µm) to purify products applying a pressure of about 0.2 bar. GC-MS (EI, 70 eV9 was performed on a combined setup of an Agilent 6890N chromatograph equipped with a HP-5 column using helium (~1 bar) as carrier gas and a Waters Micromass Quarto Micro spectrometer. Gas chromatography (GC-FID) was performed on an Agilent 7890A chromatograph equipped with a HP-5 column (30 m x 0.32 mm, film thickness 0.25 µm) using H_2 (~1 bar) as carrier gas.

X-Ray diffraction: Data sets were collected with a Nonius KappaCCD diffractometer. Programs used: data collection, COLLECT (R. W. W. Hooft, Bruker AXS, 2008, Delft, The Netherlands); data reduction Denzo-SMN (Z. Otwinowski, W. Minor, *Methods Enzymol.* **1997**, *276*, 307-326); absorption correction, Denzo (Z. Otwinowski, D. Borek, W. Majewski, W. Minor, *Acta Crystallogr.* **2003**, *A59*, 228-234); structure solution SHELXS-97 (G. M. Sheldrick, *Acta Crystallogr.* **1990**, *A46*, 467-473); structure refinement SHELXL-97 (G. M. Sheldrick, *Acta Crystallogr.* **2008**, *A64*, 112-122) and graphics, XP (BrukerAXS, 2000). *R*-values are given for observed reflections, and wR^2 values are given for all reflections.

Synthetic procedures

General Procedure 1 for the substitution reaction initiated with UV-light

4-Methylthiophenol (31 mg, 0.25 mmol, 5.0 equiv.) was dissolved in MeCN (1 mL) and NaH (15 mg, 0.40 mmol, 8.0 equiv.) was added. The resulting mixture was stirred for 1 h and then the dihalide (0.05 mmol, 1 equiv.) was added. The reaction mixture was subjected to UV light (365 nm, 30 W) for 5 min-8 h to a conversion of <50% (reaction times for each individual experiment are given below). An aliquot was taken, diluted with EtOAc and washed with water. The solvent was removed *in vacuo* and analyzed by GC-FID/GC-MS or MS.

General Procedure 2 for the substitution reaction initiated thermally

4-Methylthiophenol (31 mg, 0.25 mmol, 5.0 equiv.) was dissolved in DMSO (1 mL) and NaH (15 mg, 0.40 mmol, 8.0 equiv.) was added. The resulting mixture was stirred for 1 h and then the dihalide (0.05 mmol, 1 equiv.) was added. The reaction mixture was heated to 120 °C for 6-16 h to a conversion of <50% (reaction times for each individual experiment are given below). An aliquot was taken, diluted with EtOAc and washed with water. The solvent was removed *in vacuo* and analyzed by MALDI.

General Procedure 3 for the oligofluorene Br-I exchange

The corresponding dibromide (n mmol) was dissolved in Et_2O (n mL) and cooled to -78 °C. *n*-Butyllithium (n mmol) was added dropwise and the reaction mixture was stirred at -78 °C for 2 h. Then a solution of iodine in Et_2O was added dropwise and the reaction mixture was left to warm to room temperature overnight. The reaction was quenched by adding a saturated aqueous solution of $Na_2S_2O_3$ (5 mL). The phases were separated and the aqueous layer was extracted with Et_2O (3 x 10 mL). The combined organic layers were washed with brine, dried over $MgSO_4$ and then concentrated *in vacuo*. The crude product was dissolved in hot acetone (2 – 5 mL) and precipitated using MeOH. The diiodide products were generally obtained as a mixture with single and double proto-dehalogenated product and were analyzed by MALDI-MS.

7,7'-Diiodo-9,9,9',9'-tetraoctyl-9H,9'H-2,2'-bifluorene (10t)

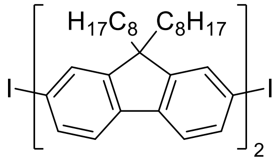

2,7-Diiodo-9,9-dioctyl-9*H*-fluorene (0.32 g, 0.50 mmol, 1.0 equiv) was dissolved in dry THF (1.5 mL) and cooled to – 40 °C. At that temperature isopropylmagnesium bromide (0.32 mL, 0.50 mmol, 1.0 equiv) was added very slowly. The reaction mixture was allowed to warm to – 20 °C before TEMPO (0.23 g, 1.5 mmol, 3.0 equiv) was added and the reaction mixture was heated to reflux immediately.[14] After refluxing over night the reaction was quenched by adding saturated aqueous ammonium chloride solution (1 mL). The mixture was extracted with MTBE (3 x) and the combined organic layers were washed with brine. After drying over magnesium sulfate and removal of the solvent, flash column chromatography (pentane) yielded the title compound as a colorless, fluorescent (UV-light) solid (0.16 g, 63%). ^1H NMR (300 MHz, $CDCl_3$, 300 K) δ (ppm) = 7.78 – 7.54 (m, 10H), 7.48 (d, 2H), 1.91 – 2.08 (m, 8H), 1.36 – 0.98 (m, 40H), 0.82 (t, J = 6.9 Hz, 12H), 0.76 – 0.62 (m, 8H). ^{13}C NMR (75 MHz, $CDCl_3$, 300 K) δ (ppm) = 153.6, 151.1, 141.1, 140.6, 139.6, 136.0, 132.3, 126.4, 121.6, 121.5, 120.2, 92.6, 55.6, 40.4,

31.9, 30.1, 29.3, 29.3, 23.9, 22.8, 14.2. HRMS (APCI): m/z = 1139.3404 calcd. for $C_{58}H_{80}I_2$ [M+Ag]$^+$, found: 1139.3406. IR: 2954 (m), 2925 (s), 2854 (m), 2366 (w), 2337 (w), 1453 (m), 1401 (w), 1256 (w), 1055 (w), 1001 (w), 884 (w), 809 (m).

7,7''-Diiodo-9,9,9',9',9'',9''-hexaoctyl-9H,9'H,9''H-2,2':7',2''-terfluorene (10u)

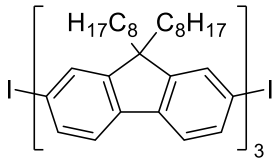

7,7''-Dibromo-9,9,9',9',9'',9''-hexaoctyl-9H,9'H,9''H-2,2':7',2''-terfluorene (150 mg, 113 µmol, 1.00 equiv) was subjected to the conditions of general procedure 3 in 20 mL of Et_2O using *n*-butyllithium (280 µL, 452 µmol, 4.00 equiv, 1.6 M solution) and a solution of iodine in Et_2O (287 mg, 1.13 mmol, 10.0 equiv in 1 mL Et_2O). A 1:1:1 mixture (ratio estimated by MALDI) of the desired product, single and double proto-dehalogenated terfluorene was obtained as a yellow sticky oil and not further purified. MS (MALDI): m/z = 1418.75 calcd. for [$C_{87}H_{120}I_2$], 1418.62 found.

7,7'''-Diiodo-9,9,9',9',9'',9'',9''',9'''-octaoctyl-9H,9'H,9''H,9'''H-2,2':7',2'':7'',2'''-quaterfluorene (10v)

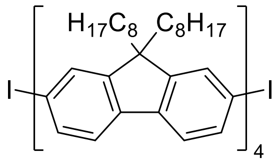

7,7'''-Dibromo-9,9,9',9',9'',9'',9''',9'''-octaoctyl-9H,9'H,9''H,9'''H-2,2':7',2'':7'',2'''-quaterfluorene (120 mg, 70 µmol, 1.00 equiv) was subjected to the conditions of general procedure 3 in 40 mL of Et_2O using *n*-butyllithium (130 µL, 210 µmol, 3.00 equiv) and a solution of iodine in Et_2O (89 mg, 350 µmol, 5.00 equiv in 2 mL of Et_2O). A mixture of the product and once and twice proto-dehalogenated quaterfluorene was obtained as a yellow sticky oil and not further purified. MS (MALDI): m/z = 1808.06 calcd. for [$C_{116}H_{160}I_2$], 1808.23 found.

7,7''''-Diiodo-9,9,9',9',9'',9'',9''',9''',9'''',9''''-decaoctyl-9H,9'H,9''H,9'''H,9''''H-2,2':7',2'':7'',2''':7''',2''''-quinquefluorene (10w)

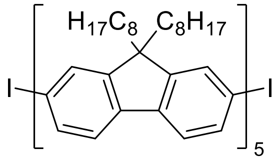

7,7''''-Dibromo-9,9,9',9',9'',9'',9''',9''',9'''',9''''-decaoctyl-9H,9'H,9''H,9'''H,9''''H-2,2':7',2'':7'',2''':7''',2''''-quinquefluorene (110 mg, 52 µmol, 1.00 equiv) was subjected to the conditions of general procedure 3 in 20 mL of THF using *tert*-butyllithium (60 µL, 105 µmol, 2.00 equiv), *n*-butyllithium (200 µL, 314 µmol, 6.00 equiv, 1.6 M solution) and a solution of iodine in THF (134 mg, 523 µmol, 10.0 equiv, in 1 mL of THF). A 1:1:1 mixture (ratio approximated by MALDI) of the desired product, single and double proto-dehalogenated terfluorene was obtained as a yellow sticky oil and not further purified. MS (MALDI): m/z = 2195.37 calcd. for [$C_{145}H_{200}I_2$], 2195.29 found.

3,7-Diiododibenzo[b,d]thiophene (10n)

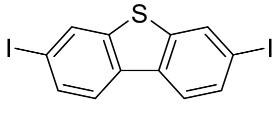

3,7-Diiododibenzo[b,d]thiophene 5,5-dioxide[9] (0.23 g, 0.50 mmol, 1.0 equiv) was dissolved in THF (20 mL) and DIBAL-H (1 M in THF, 2.5 mL, 2.5 mmol, 5.0 equiv) was added dropwise and the reaction mixture was heated to 50 °C for 24 hours. The reaction was quenched by adding water and the phases were separated. The aqueous layer was extracted with ether (3 x), washed with water and brine and dried over magnesium sulfate. After removal of the solvent *in vacuo* recrystallization from hot chloroform yielded pure 3,7-

diiododibenzo[b,d]thiophene as white needles (180 mg, 83%). ^{1}H NMR (300 MHz, $CDCl_3$, 300 K) δ (ppm) = 8.18 (d, ^4J = 1.4 Hz, 2H, 1-H), 7.85 (d, ^3J = 8.3 Hz, 2H, 3-H), 7.76 (dd, ^3J = 8.3 Hz, ^4J = 1.4 Hz, 2H, 4-H). ^{13}C NMR (75 MHz, C_6D_6, 300 K) δ (ppm) = 141.3, 134.4, 133.7, 131.5, 123.0, 92.2.

Dimethyl 4,4'-diiodo-biphenyl-2,2'-dicarboxylate

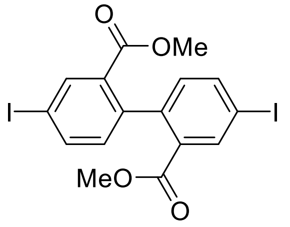

4,4'-Diiodo-[1,1'-biphenyl]-2,2'-dicarboxylic acid[11] (494 mg, 1.00 mmol, 1.00 equiv) was dissolved in methanol (6 mL) and sulfuric acid (530 µL, 10.0 mmol, 10.0 equiv) was added carefully and the reaction was refluxed for 20 hours. After cooling to room temperature the mixture was diluted with ether, washed with water (2 x), aqueous saturated solution of sodium bicarbonate and brine, dried over magnesium sulfate and the solvent was removed *in vacuo*. Flash column chromatography (10% ethyl acetate in pentane) yielded dimethyl 4,4'-diiodo-biphenyl-2,2'-dicarboxylate as a white solid (189 mg, 36%). ^{1}H NMR (300 MHz, C_6D_6, 300 K) δ (ppm) = 8.34 (d, ^4J = 1.9 Hz, 2H, 3/3'-H), 7.85 (dd, ^3J = 8.1 Hz, ^4J = 1.9 Hz, 2H, 5/5'-H), 6.90 (d, ^3J = 8.1 Hz, 2H, 6/6'-H), 3.66 (s, 6H, CH_3). ^{13}C NMR (75 MHz, $CDCl_3$, 300 K) δ (ppm) = 165.8, 142.0, 140.7, 138.9, 131.8, 130.8, 92.7, 52.3, 29.8.

(4,4'-Diiodo-biphenyl-2,2'-diyl)dimethanol

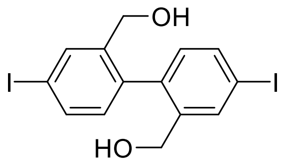

Dimethyl 4,4'-diiodo-biphenyl-2,2'-dicarboxylate (0.15 g, 0.29 mmol, 1.0 equiv) was added to a 1 M solution of DIBAL-H (1.9 mL, 1.9 mmol, 6.6 equiv) at 0 °C. After the addition, the mixture was allowed to warm to room temperature and stirring was continued overnight. The reaction was quenched by adding water (0.5 mL), 15% sodium hydroxide solution (0.5 mL) and water (1 mL) sequentially at 0 °C. After stirring for 30 minutes at room temperature the mixture was extracted with ether (3 x). The combined organic layers were washed with brine, dried over magnesium sulfate and the solvent was removed *in vacuo* to yield (4,4'-diiodo-[1,1'-biphenyl]-2,2'-diyl)dimethanol as a white solid (0.13 g, 97%). ^{1}H NMR (300 MHz, DMSO-d_6, 300 K) δ (ppm) = 7.88 (d, ^4J = 1.8 Hz, 2H, 3/3'-H), 7.64 (dd, ^3J = 8.0 Hz, ^4J = 1.8 Hz, 2H, 5/5'-H), 6.86 (d, ^3J = 8.0 Hz, 2H, 6-H), 5.20 (t, ^3J = 5.5 Hz, 2H, OH), 4.09 (qd, ^3J = 14.0 Hz, ^4J = 5.5 Hz, 4H, CH_2). ^{13}C NMR (75 MHz, DMSO-d_6, 300 K) δ (ppm) = 142.3 (C_q), 136.6 (C_q), 135.5 (CH), 135.1 (CH), 131.01 (CH), 94.0 (C_q), 60.0 (CH_2). HRMS (ESI): m/z = 488.8819 calcd. for $C_{14}H_{12}O_2I_2Na$ [M+Na$^+$], found: 488.8802. IR: 3303 (s), 1701 (m), 1580 (m), 1465 (m), 1420 (m), 1389 (m), 1361 (w), 1338 (w), 1247 (w), 1186 (m), 1141 (w), 1112 (w), 1080 (m), 1038 (s), 1001 (s), 889 (w), 834 (m), 808 (m), 757 (m). Mp: 140-141 °C.

2,2'-Bis(bromomethyl)-4,4'-diiodo-biphenyl

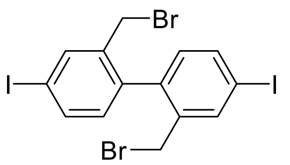

(4,4'-Diiodo-[1,1'-biphenyl]-2,2'-diyl)dimethanol (0.12 g, 0.26 mmol, 1.0 equiv) was suspended in dry DCM (2.5 mL) and PBr_3 (60 µL, 0.64 mmol, 2.5 equiv) was added at 0 °C. The reaction mixture was allowed to warm to room temperature and stirring was continued for 24 h. After carefully quenching with water at 0 °C, the phases were separated and the aqueous layer was extracted with DCM (3 x). The combined organic layers were washed with brine, dried over magnesium sulfate and the solvent was removed *in vacuo*. Flash column chromatography (pentane) yielded pure 2,2'-bis(bromomethyl)-4,4'-diiodo-1,1'-

biphenyl as a white solid (86 mg, 56%). ^{1}H NMR (300 MHz, $CDCl_3$, 300 K) δ (ppm) = 7.89 (d, ^4J = 1.8 Hz, 2H, 3/3'-H), 7.70 (dd, ^3J = 8.0 Hz, ^4J = 1.8 Hz, 2H, 5/5'-H), 6.98 (d, ^3J = 8.0 Hz, 2H, 6/6'-H), 4.25 – 4.04 (m, 4H, CH_2). ^{13}C NMR (75 MHz, $CDCl_3$, 300 K) δ (ppm) = 139.7 (CH), 138.13 (C_q), 138.0 (C_q), 137.6 (CH), 131.7 (CH), 94.4 (C_q), 30.3 (CH_2). IR: 2919 (w), 2850 (w), 1578 (m), 1547 (w), 1467 (s), 1439 (w), 1385 (w), 1266 (w), 1215 (s), 1191 (m), 1147 (w), 1078 (m), 1055 (m), 1028 (m), 998 (s), 888 (w), 871 (w), 841 (m), 820 (s). Mp: 165 °C.

2,2'-Diallyl-4,4'-diiodo-biphenyl

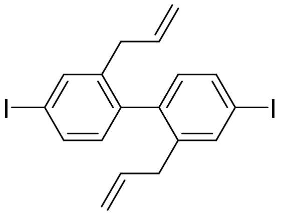

Copper iodide (77 mg, 0.41 mmol, 6.0 equiv) was suspended in dry THF (1 mL) under argon atmosphere and cooled to – 70 °C before a 1 M solution vinylmagnesium bromide in THF (0.81 mL, 0.81 mmol, 3.0 equiv) was added excluding light. After stirring for ten minutes at that temperature, 2,2'-bis(bromomethyl)-4,4'-diiodo-1,1'-biphenyl (80 mg, 0.14 mmol, 1.0 equiv) dissolved in THF (1 mL) was added and the reaction mixture was allowed to warm gradually over night. After quenching with saturated aqueous ammonium chloride and filtration through a celite pad the organic layer was washed with water (2 x) and brine, dried over magnesium sulfate and the solvent was removed *in vacuo*. Flash column chromatography yielded 2,2'-diallyl-4,4'-diiodo-1,1'-biphenyl as a colorless oil (25 mg, 38%). ^{1}H NMR (300 MHz, $CDCl_3$, 300 K) δ (ppm) = 7.63 (d, ^4J = 1.9 Hz, 2H, 3/3'-H), 7.56 (dd, ^3J = 8.0 Hz, ^4J = 1.9 Hz, 2H, 5/5'-H), 6.81 (d, ^3J = 8.0 Hz, 2H, 6/6'-H), 5.73 (ddt, ^3J = 17.0, 10.0, 6.6 Hz, 2H, $RCH_2HC{=}CH_2$), 5.00 (dq, ^3J = 10.0 Hz, ^4J = 1.3 Hz, 2H, RCH=C*H*H), 4.87 (dq, ^3J = 17.0 Hz, ^4J = 1.6 Hz, 2H, RCH=CH*H*), 3.17 – 2.88 (m, 4H). ^{13}C NMR (75 MHz, $CDCl_3$, 300K) δ (ppm) = 139.4 (C_q), 138.4 (C_q), 137.4 (CH), 135.1 (CH), 134.2 (CH), 130.6 (CH), 115.9 (CH_2), 92.7 (C_q), 36.3 (CH_2). HRMS (APCI): m/z = 485.9336 calcd. for $C_{18}H_{16}I_2$ [M^+], found: 485.9333. IR: 3077 (w), 2976 (w), 2921 (w), 2851 (w), 1733 (w), 1638 (w), 1578 (m), 1465 (s), 1383 (m), 1260 (m), 1182 (m), 1079 (s), 1000 (s), 916 (s), 817 (s), 671 (m).

(Z)-3,10-Diiodo-5,8-dihydrodibenzo[a,c][8]annulene

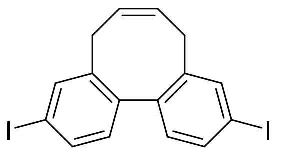

2,2 '-Diallyl-4,4'-diiodo-1,1'-biphenyl (25 mg, 51 µmol, 1.0 equiv) and Grubbs catalyst 2nd generation (3 mg, 3 µmol, 6 mol-%) were dissolved in dry DCM and heated to reflux for five hours. After that the solvent was removed and subsequent flash column chromatography yielded the title compound as a yellow solid (20 mg, 85%). ^{1}H NMR (300 MHz, $CDCl_3$, 300 K) δ (ppm) = 7.61 (dd, ^3J = 7.9 Hz, ^4J = 1.8 Hz, 2H, 4/9-H), 7.56 (d, ^4J = 1.8 Hz, 2H, 2/11-H), 6.97 (d, ^3J = 7.9 Hz, 2H, 1/12-H), 5.92 – 5.71 (m, 2H, R*H*C=C*H*R), 3.09 – 3.00 (m, 2H, C*H*H), 2.96 – 2.82 (m, 2H, CH*H*). ^{13}C NMR (75 MHz, $CDCl_3$, 300 K) δ (ppm) = 140.7 (C_q), 139.2 (C_q), 138.3 (CH), 135.5 (CH), 129.5 (CH), 129.0 (CH), 94.2 (C_q), 33.0 (CH_2). HRMS (APCI): m/z = 564.8074 calcd. for $C_{16}H_{12}I_2Ag$ [$M+Ag^+$], found: 564.8076. IR: 3018 (m), 2931 (w), 2901 (w), 2842 (w), 1897 (w), 1648 (w), 1580 (m), 1470 (m), 1434 (m), 18 (m), 1279 (w), 1195 (m), 1082 (m), 1003 (s), 906 (w), 906 (w), 85 (m) 830 (m), 812 (m). Mp: 183 °C.

3,10-Diiodo-5,6,7,8-tetrahydrodibenzo[a,c][8]annulene (10k)

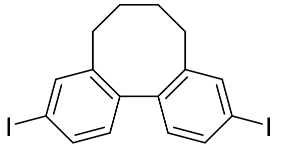

(Z)-3,10-Diiodo-5,8-dihydrodibenzo[a,c][8]annulene (17 mg, 37 µmol, 1.0 equiv) and palladium on charcoal [10 weight-%] (1 mg, 1 µmol, 3 mol-%) were evacuated in a pressure tube and purged with hydrogen. Then ethyl acetate (1 mL) was added and the mixture was stirred for three hours at room temperature. The solvent was removed *in vacuo* and the residue purified by flash column chromatography (pentane) to yield the title compound as a white solid (17 mg, 99%). ^{1}H NMR (300 MHz, $CDCl_3$, 300 K) δ (ppm) = 7.63 (d, ^{4}J = 1.8 Hz, 2H, 3/3'-H), 7.57 (dd, ^{3}J = 8.0 Hz, ^{4}J = 1.8 Hz, 2H, 5/5'-H), 6.92 (d, ^{3}J = 8.0 Hz, 2H, 6/6'-H), 2.70 – 2.63 (m, 2H, H_{alkyl}), 2.17 – 1.96 (m, 4H, H_{alkyl}), 1.61 – 1.35 (m, 2H, H_{alkyl}). ^{13}C NMR (75 MHz, $CDCl_3$, 300 K) δ (ppm) = 145.1 (C_q), 139.2 (C_q), 138.4 (CH), 135.0 (CH), 130.7 (CH), 94.0 (C_q), 32.5 (CH_2), 29.3 (CH_2). HRMS (APCI): m/z = 517.2958 calcd. for $C_{30}H_{50}Ag$ [M+Ag$^+$], found: 517.2963. IR: 2926 (s), 2852 (m), 2360 (w), 1732 (w), 1579 (m), 1467 (s), 1383 (m), 1187 (m), 1078 (m), 1001 (s), 890 (m), 810 (s). Mp: 122 °C.

3,3'-Bis(trimethylsilyl)-biphenyl

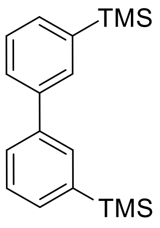

Magnesium turnings (800 mg, 33.0 mg, 1.25 equiv.) were placed in a flame dried three-necked flask with a reflux condenser and stirred under vacuum overnight. Et_2O (10 mL) was added along with 1,2-dibromo ethane (100 µL). Then a solution of 1-bromo-3-(trimethylsilyl)benzene (5.0 mL, 26 mmol, 1.0 equiv.) and 1,2-dibromo ethane (100 µL) in Et_2O (10 mL) was added over 1 h maintaining steady reflux. After stirring for additional 2 h at reflux (external heating source) and cooling down to room temperature the Grignard solution was titrated and a concentration of 1.3 M was obtained. The Grignard solution (1.55 mL, 1.3 M in Et_2O, 2.00 mmol, 1.00 equiv.) was then added to a solution of TEMPO (344 mg, 2.20 mmol, 1.10 equiv.) in THF (3 mL) and heated to reflux overnight. After cooling to room temperature the reaction was quenched with water and extracted with Et_2O (3 x). The combined organic extracts were washed with water and brine, dried over $MgSO_4$ and the solvent was removed *in vacuo*. FCC (100% pentane) afforded the title compound as a colorless oil (150 mg, 50%). ^{1}H NMR (300 MHz, $CDCl_3$, 300 K) δ (ppm) = 7.74 – 7.67 (m, 2H), 7.63 – 7.49 (m, 4H), 7.48 – 7.41 (m, 2H), 0.32 (s, 18H). ^{13}C NMR (75 MHz, $CDCl_3$, 300 K) δ (ppm) = 141.1, 132.5, 132.3, 128.2, 128.1, -0.9.

3,3'-Diiodo-biphenyl (10i)

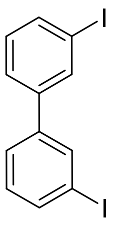

3,3'-Bis(trimethyl silyl)-biphenyl (100 mg, 335 µmol, 1.00 equiv.) was dissolved in CH_2Cl_2 (2 mL) and cooled to 0 °C in an ice-bath. A solution of ICl (40 µL, 0.77 mmol. 2.3 equiv.) in CH_2Cl_2 (0.5 mL) was added and the reaction mixture was stirred at 0 °C for 1 h and then at room temperature for another hour. The reaction was quenched with a saturated aqueous solution of $Na_2S_2O_3$ and extracted with CH_2Cl_2 (3 x). The combined organic layers were washed with brine, dried over $MgSO_4$ and the solvent was removed *in vacuo*. FCC with pentane as eluent afforded the title compound as a white solid (84 mg, 62%). ^{1}H NMR (300 MHz, $CDCl_3$, 300 K) δ (ppm) = 7.89 (t, ^{4}J = 1.7 Hz, 2H), 7.75 – 7.65 (m, 2H), 7.53 – 7.45 (m, 2H), 7.17 (t, ^{3}J = 7.8 Hz, 2H). ^{13}C NMR (75 MHz, $CDCl_3$, 300 K) δ (ppm) = 141.9, 136.9, 136.2, 130.6, 126.5, 95.0.

References

[1] W. Jiang, H. Xu, X. Ban, G. Yuan, Y. Sun, B. Huang, L. Duan, Y. Qiu, *Org. Lett.* **2014**, *16*, 1140-1143.
[2] B. Balan, C. Vijayakumar, S. Ogi, M. Takeuchi, *J. Mater. Chem.* **2012**, *22*, 11224-11234.
[3] K. Kokado, Y. Tokoro, Y. Chujo, *Macromolecules* **2009**, *42*, 9238-9242.
[4] M. S. Maji, T. Pfeifer, A. Studer, *Chem. Eur. J.* **2010**, *16*, 5872-5875.
[5] N. G. Pschirer, K. Byrd, U. H. F. Bunz, *Macromolecules* **2001**, *34*, 8590-8592.
[6] D. Vonlanthen, J. Rotzler, M. Neuburger, M. Mayor, *Eur. J. Org. Chem.* **2010**, *2010*, 120-133.
[7] K. L. Chan, S. E. Watkins, C. S. K. Mak, M. J. McKiernan, C. R. Towns, S. I. Pascu, A. B. Holmes, *Chemical Communications* **2005**, 5766-5768.
[8] M. S. Inkpen, S. Du, M. Driver, T. Albrecht, N. J. Long, *Dalton Trans.* **2013**, *42*, 2813-2816.
[9] F. Yang, K. Sun, Z. J. Cao, Z. H. Li, M. S. Wong, *Synth. Met.* **2008**, *158*, 391-395.
[10] R. Qin, Z. Bo, *Macromol. Rapid Commun.* **2012**, *33*, 87-91.
[11] É. Balogh-Hergovich, G. Speier, Z. Tyeklár, *Synthesis* **1982**, *1982*, 731-732.
[12] T. Shiraki, S. Shindome, F. Toshimitsu, T. Fujigaya, N. Nakashima, *Polymer Chemistry* **2015**, *6*, 5103-5109.
[13] X. Liu, Q. Yi, Y. Han, Z. Liang, C. Shen, Z. Zhou, J.-l. Sun, Y. Li, W. Du, R. Cao, *Angew. Chem. Int. Ed.* **2015**, *54*, 1846-1850.
[14] M. S. Maji, T. Pfeifer, A. Studer, *Angew. Chem. Int. Ed.* **2008**, *47*, 9547-9550.

NMR Spectra

3,7-Diiododibenzo[b,d]thiophene (10n)

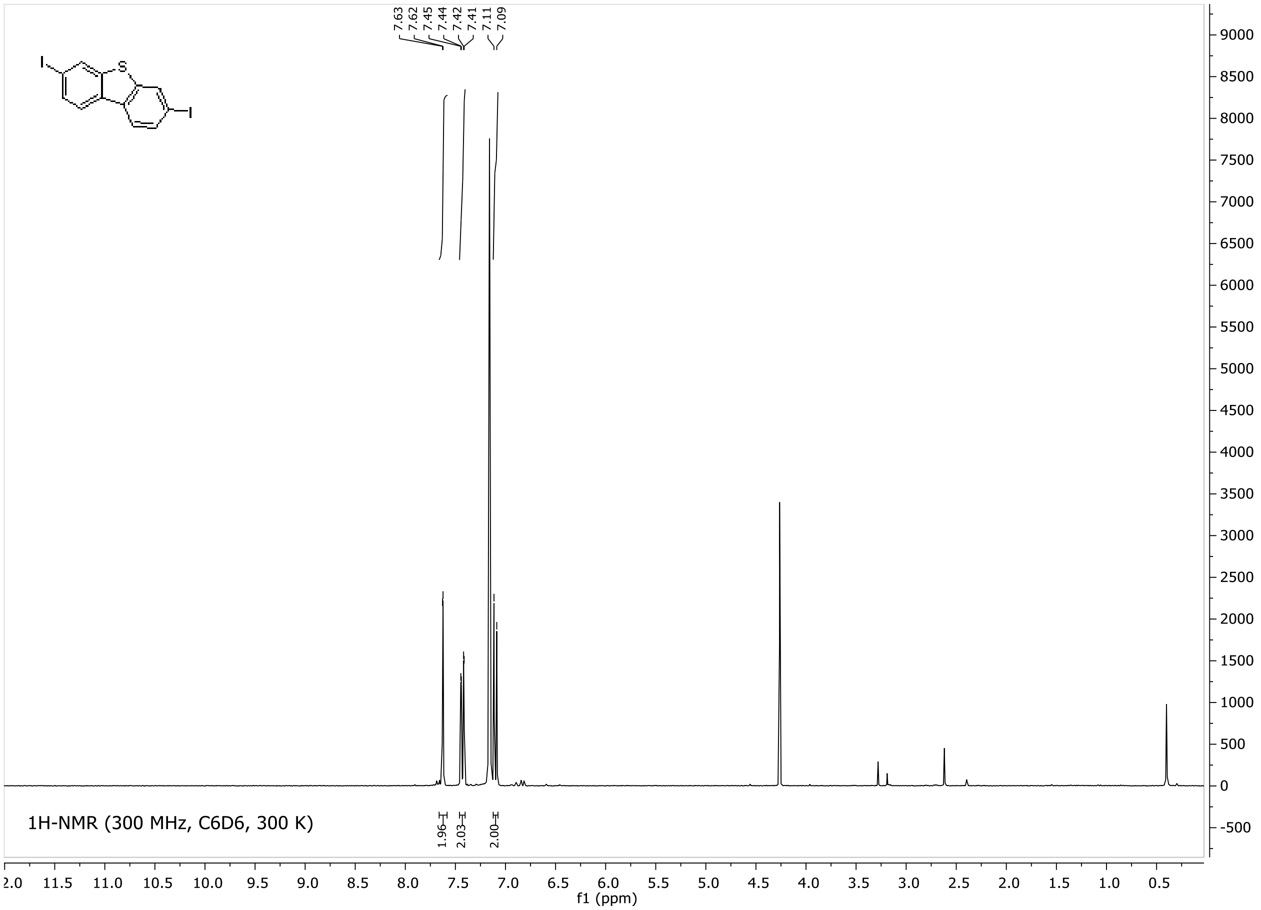

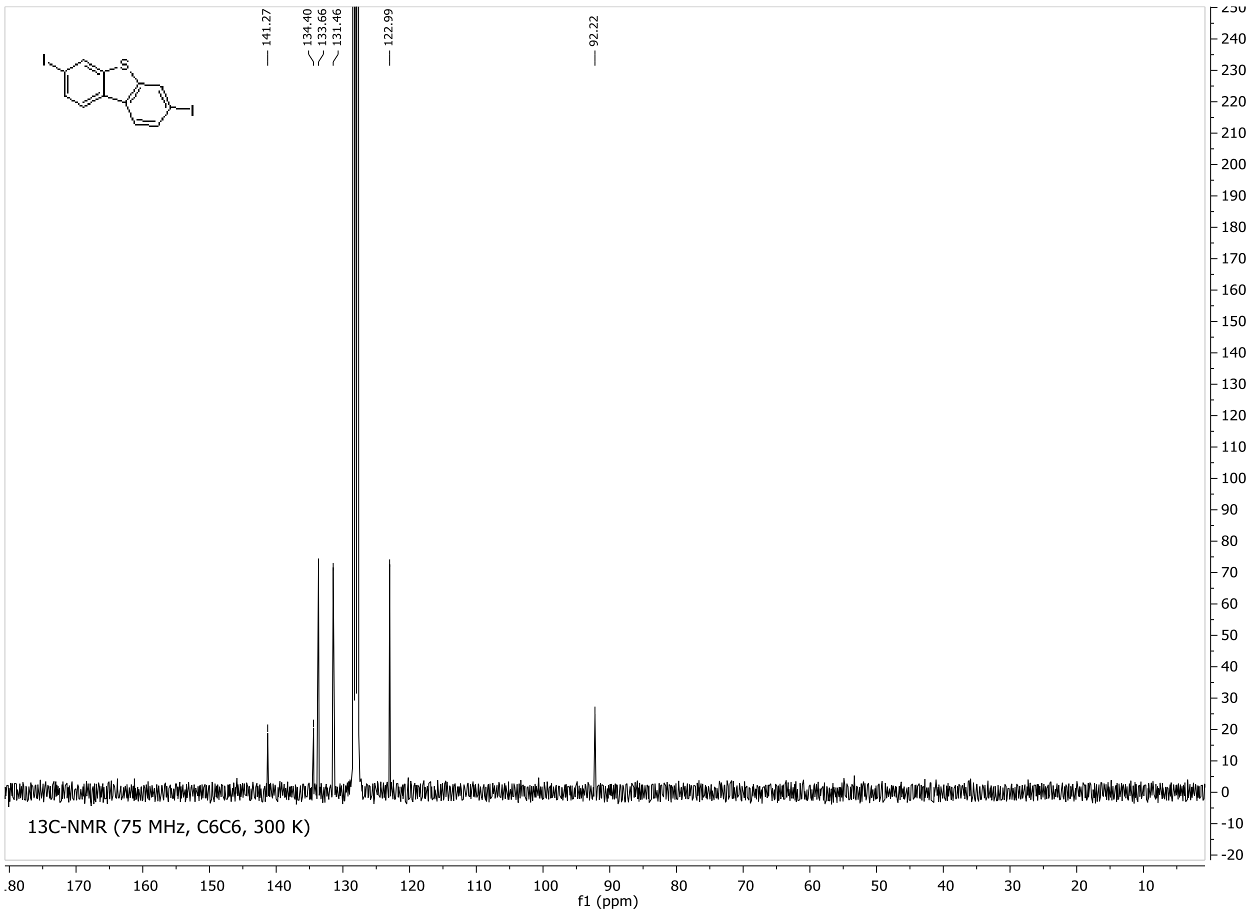
141.27
134.40
133.66
131.46
122.99
92.22
13C-NMR (75 MHz, C6C6, 300 K)
f1 (ppm)

(4,4'-Diiodo-biphenyl-2,2'-diyl)dimethanol

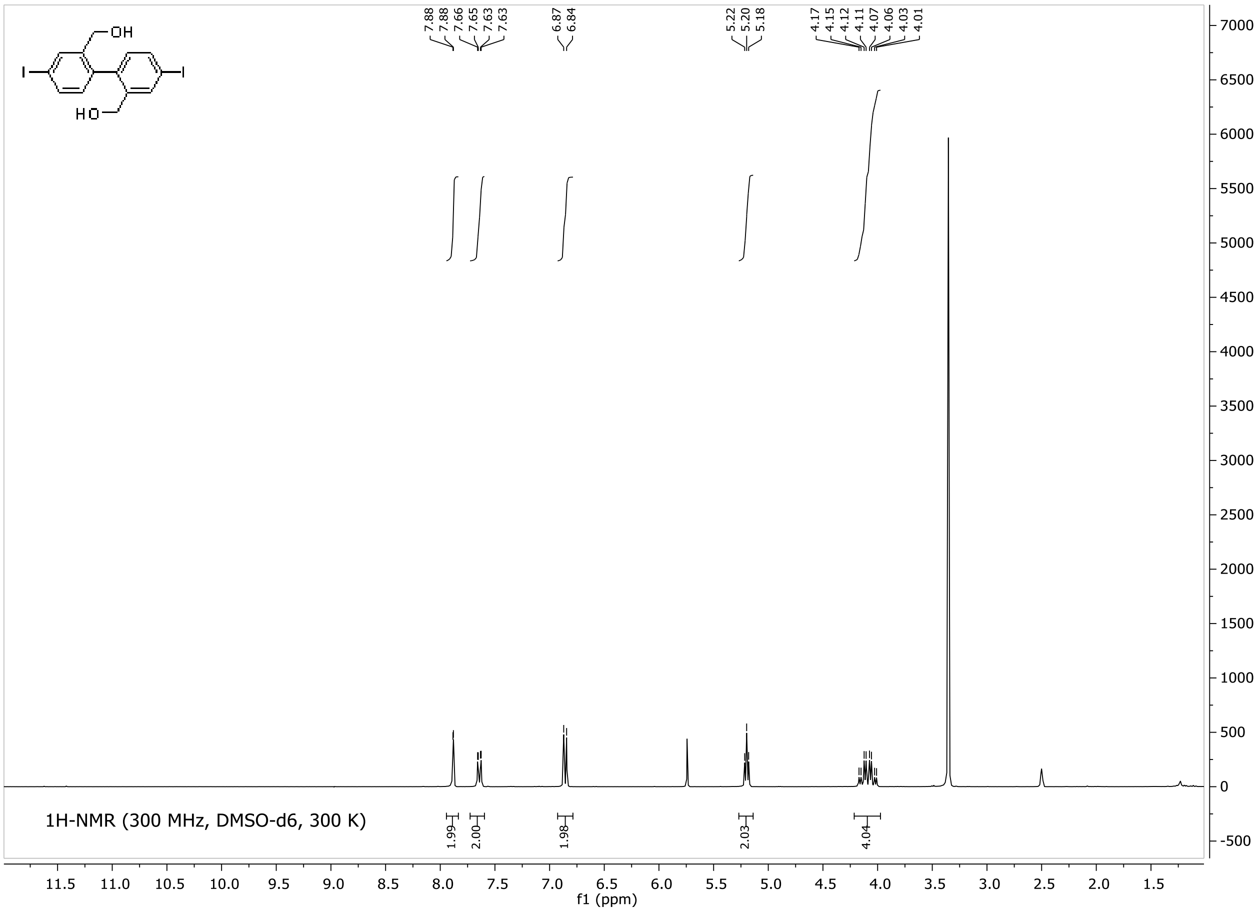

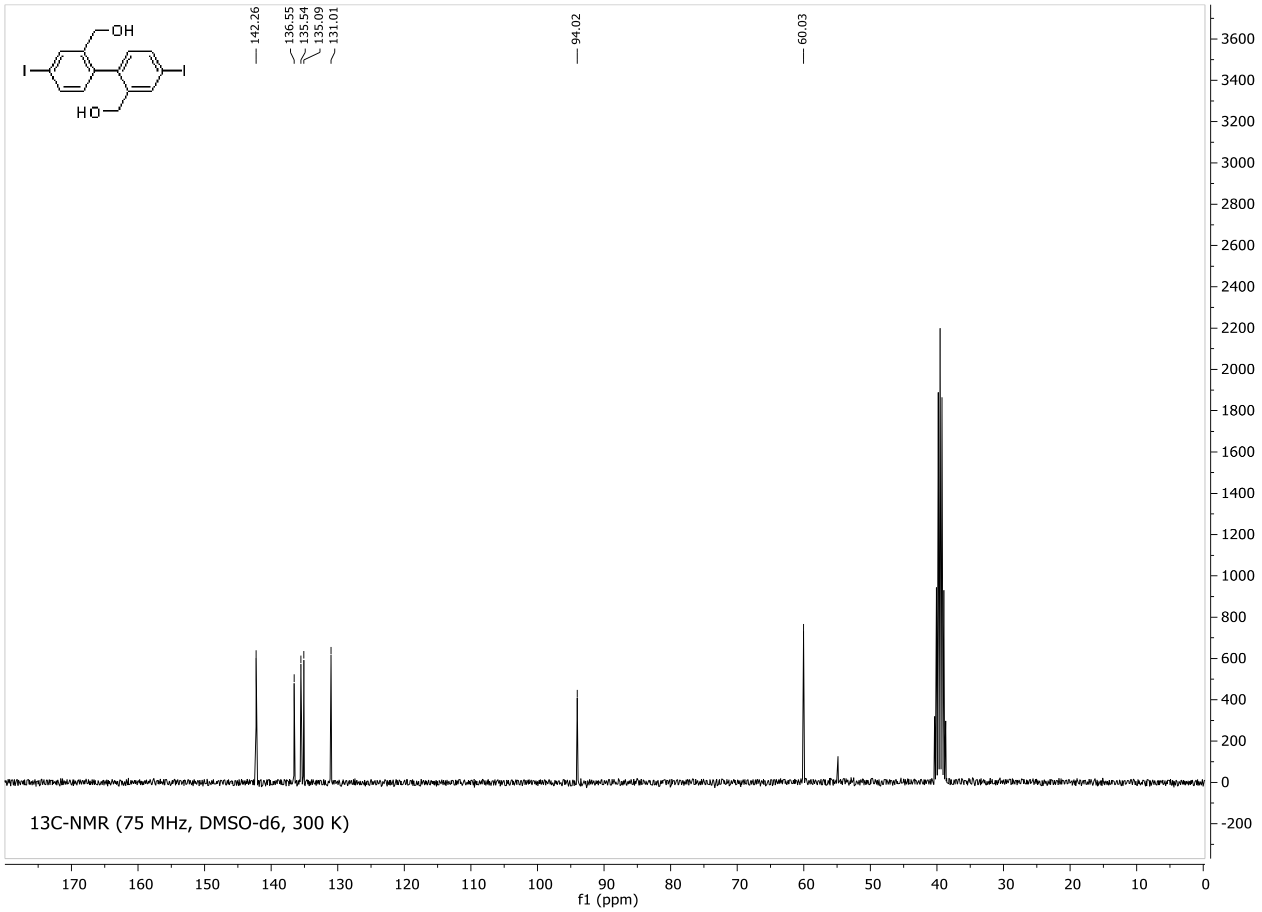

OH
HO
I
I
142.26
136.55
135.54
135.09
131.01
94.02
60.03
13C-NMR (75 MHz, DMSO-d6, 300 K)
f1 (ppm)

2,2'-Bis(bromomethyl)-4,4'-diiodo-biphenyl

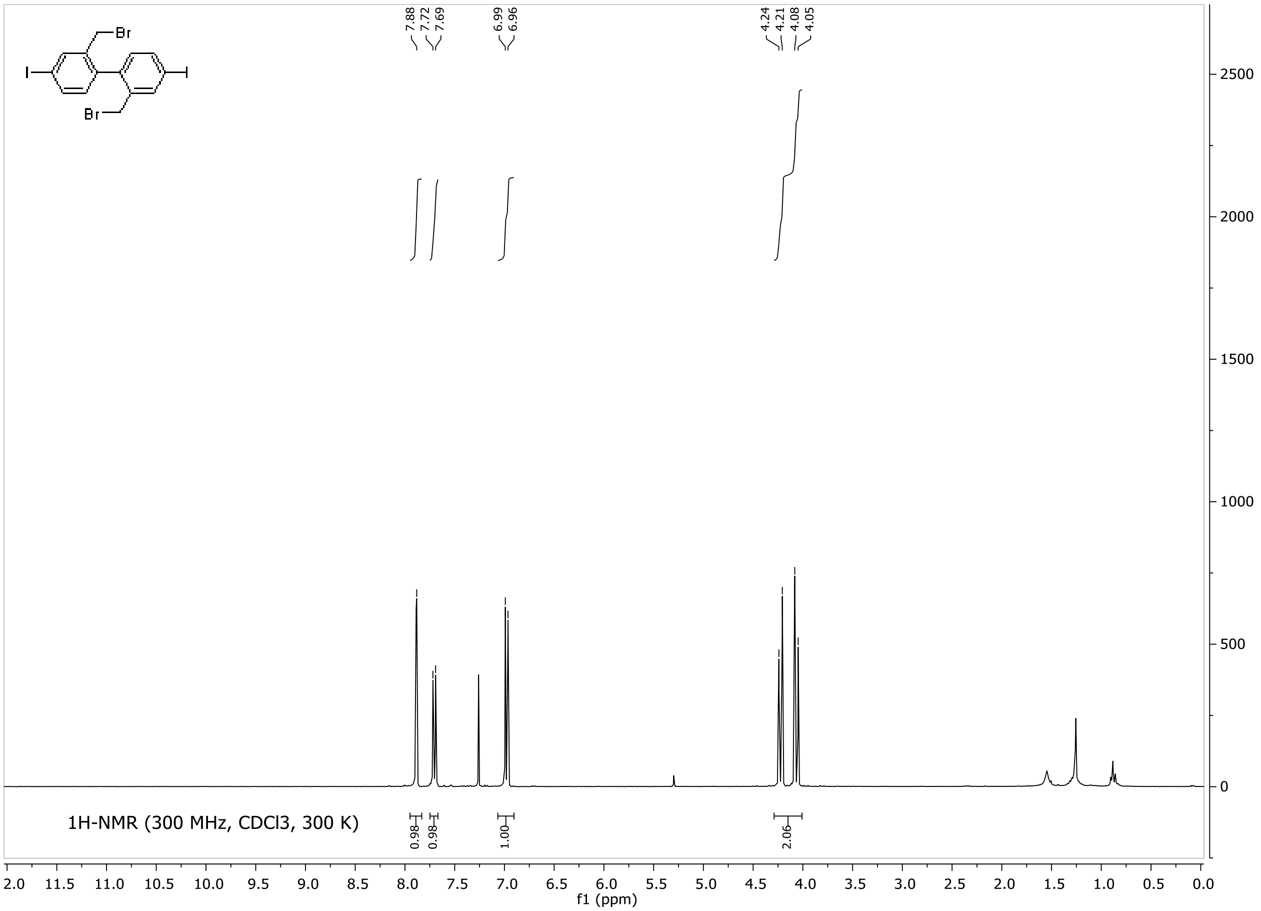

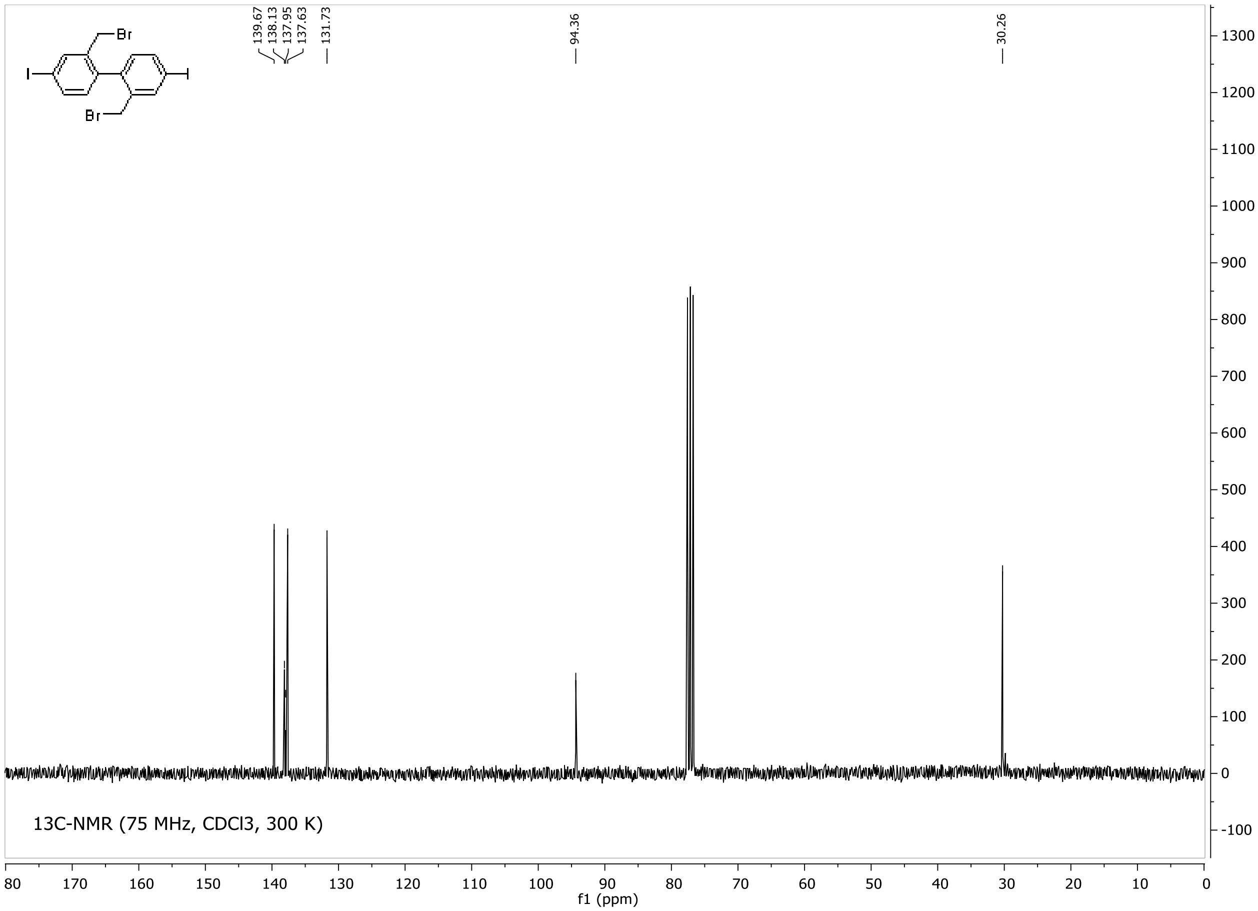
Br
Br
I
I
139.67
138.13
137.95
137.63
131.73
94.36
30.26
13C-NMR (75 MHz, CDCl3, 300 K)
f1 (ppm)

2,2'-Diallyl-4,4'-diiodo-biphenyl

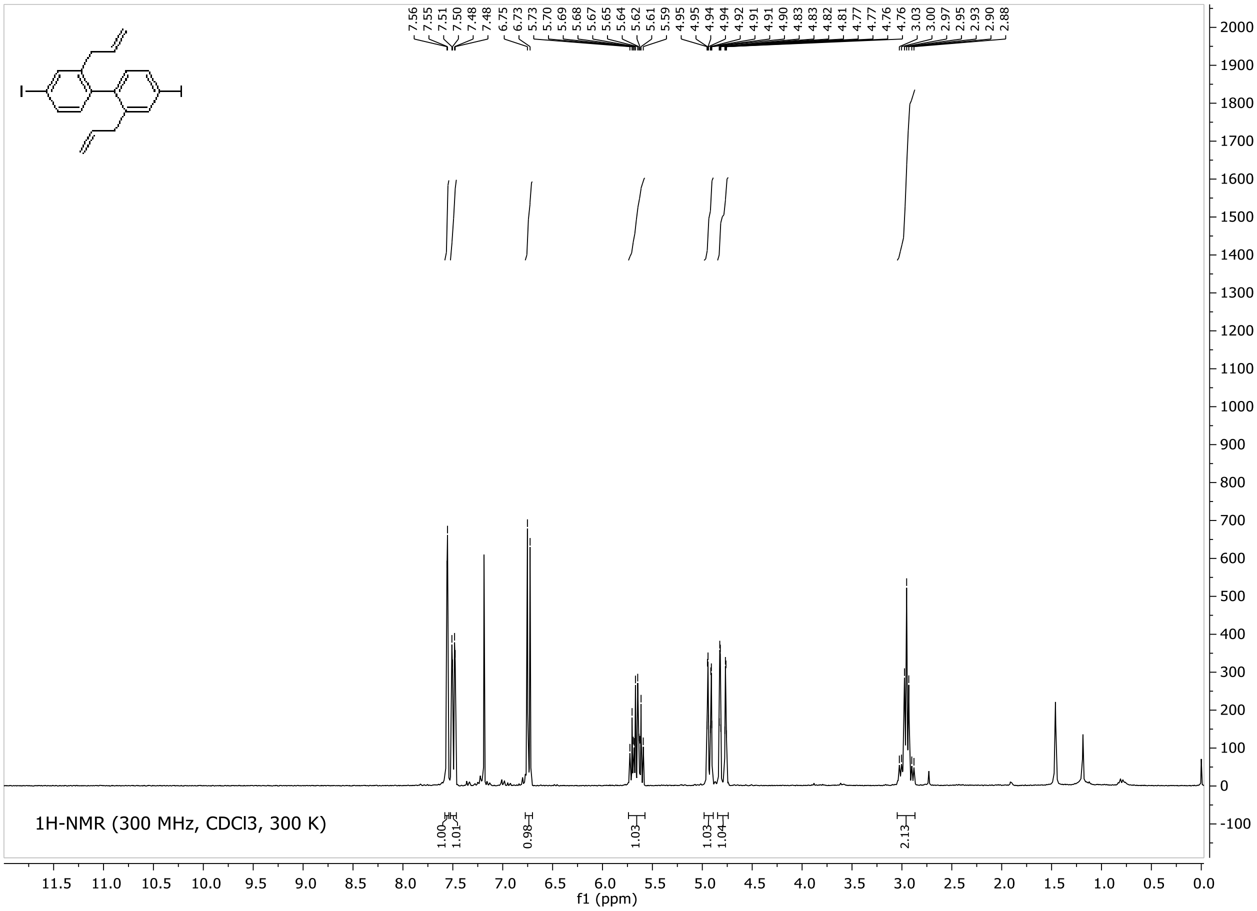

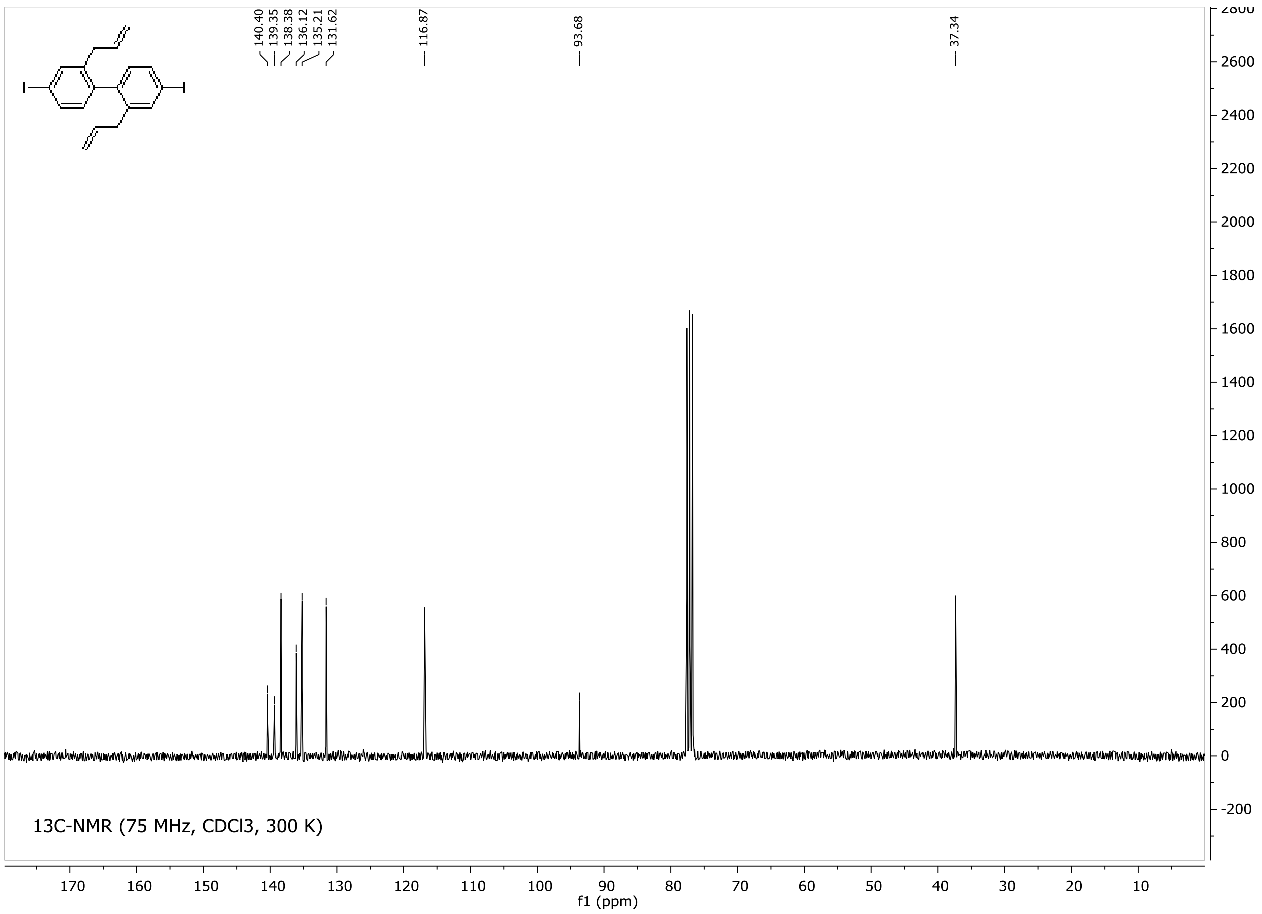

140.40
139.35
138.38
136.12
135.21
131.62
116.87
93.68
37.34
13C-NMR (75 MHz, CDCl3, 300 K)
f1 (ppm)

(Z)-3,10-Diiodo-5,8-dihydrodibenzo[a,c][8]annulene

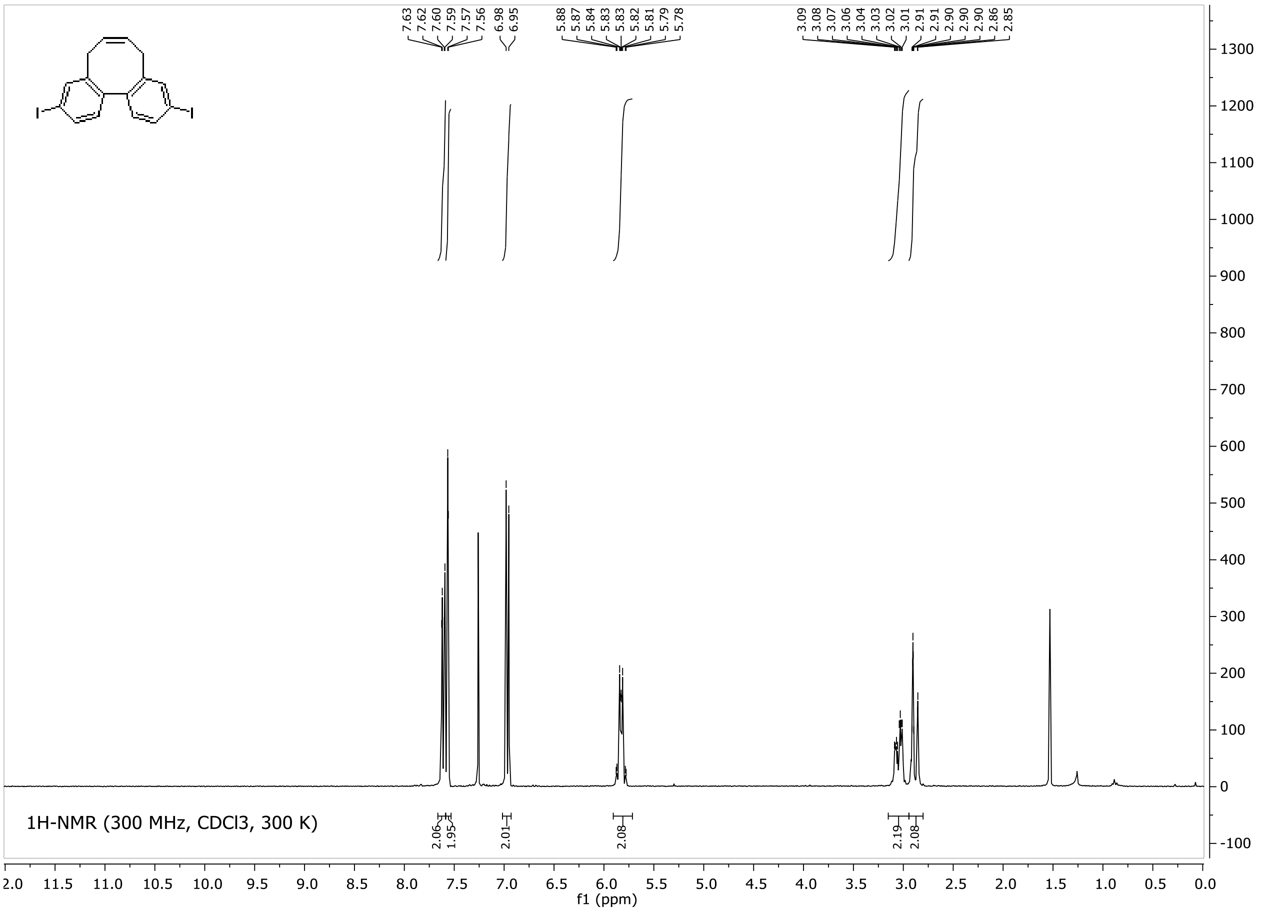

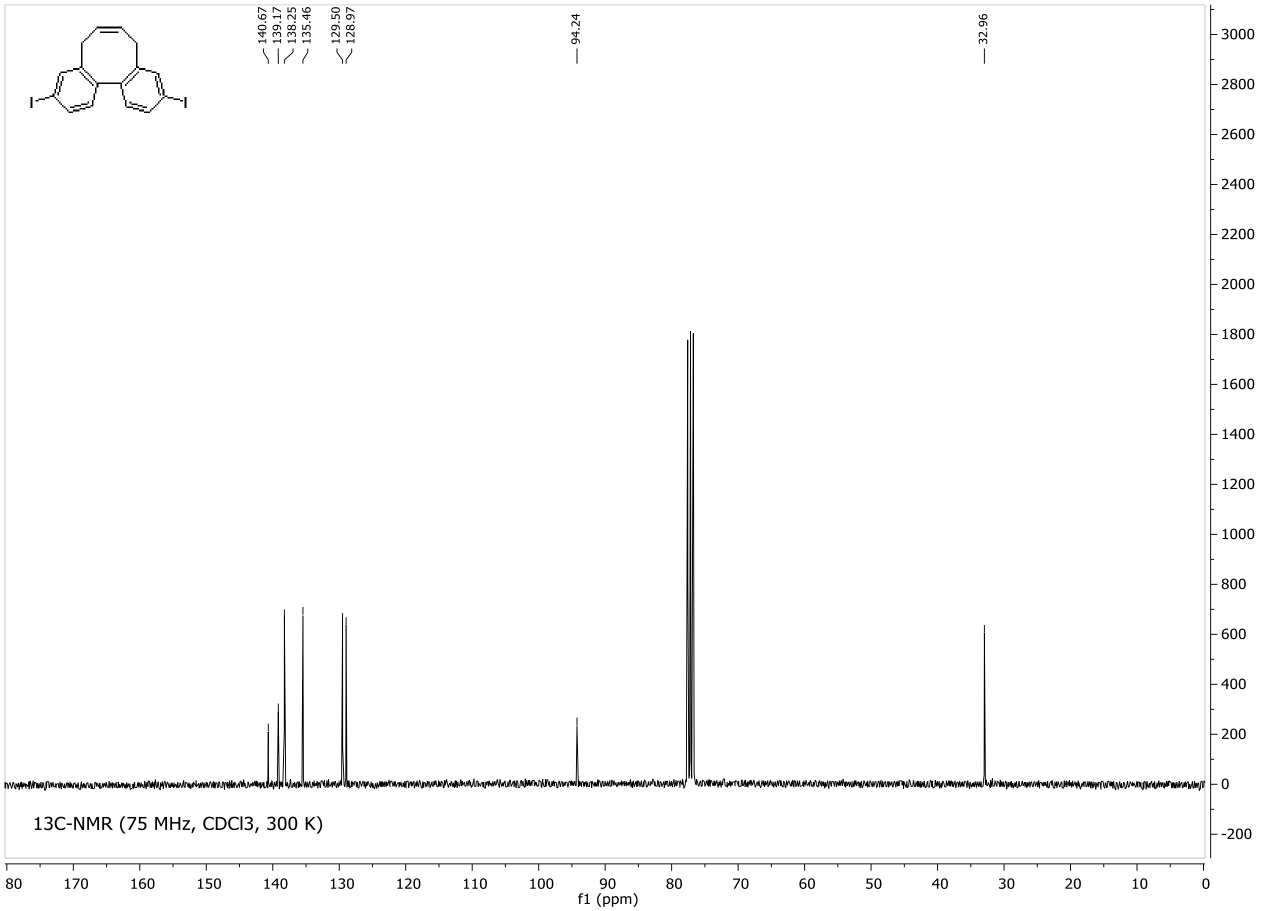
140.67
139.17
138.25
135.46
129.50
128.97
94.24
32.96
13C-NMR (75 MHz, CDCl3, 300 K)
f1 (ppm)

3,10-Diiodo-5,6,7,8-tetrahydrodibenzo[a,c][8]annulene (10k)

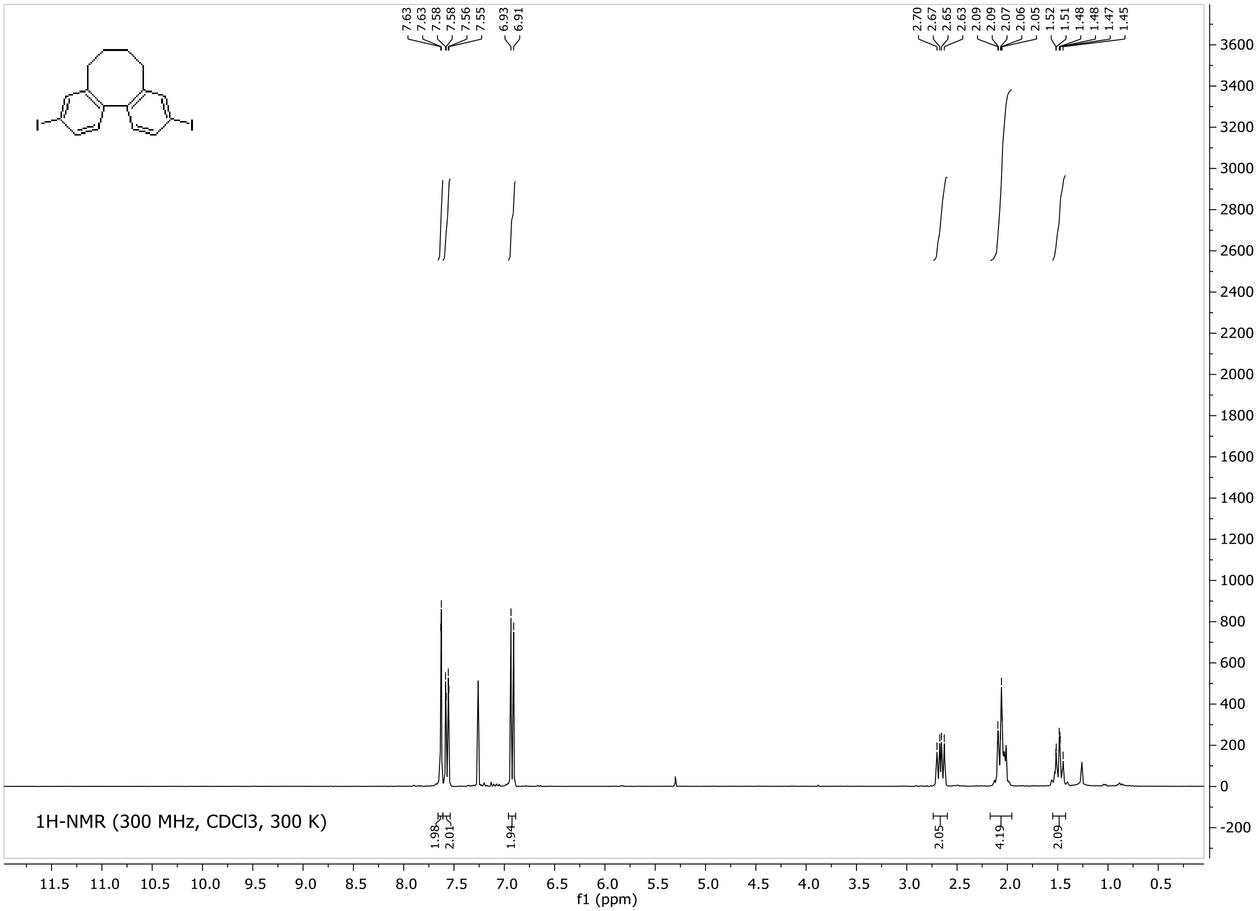

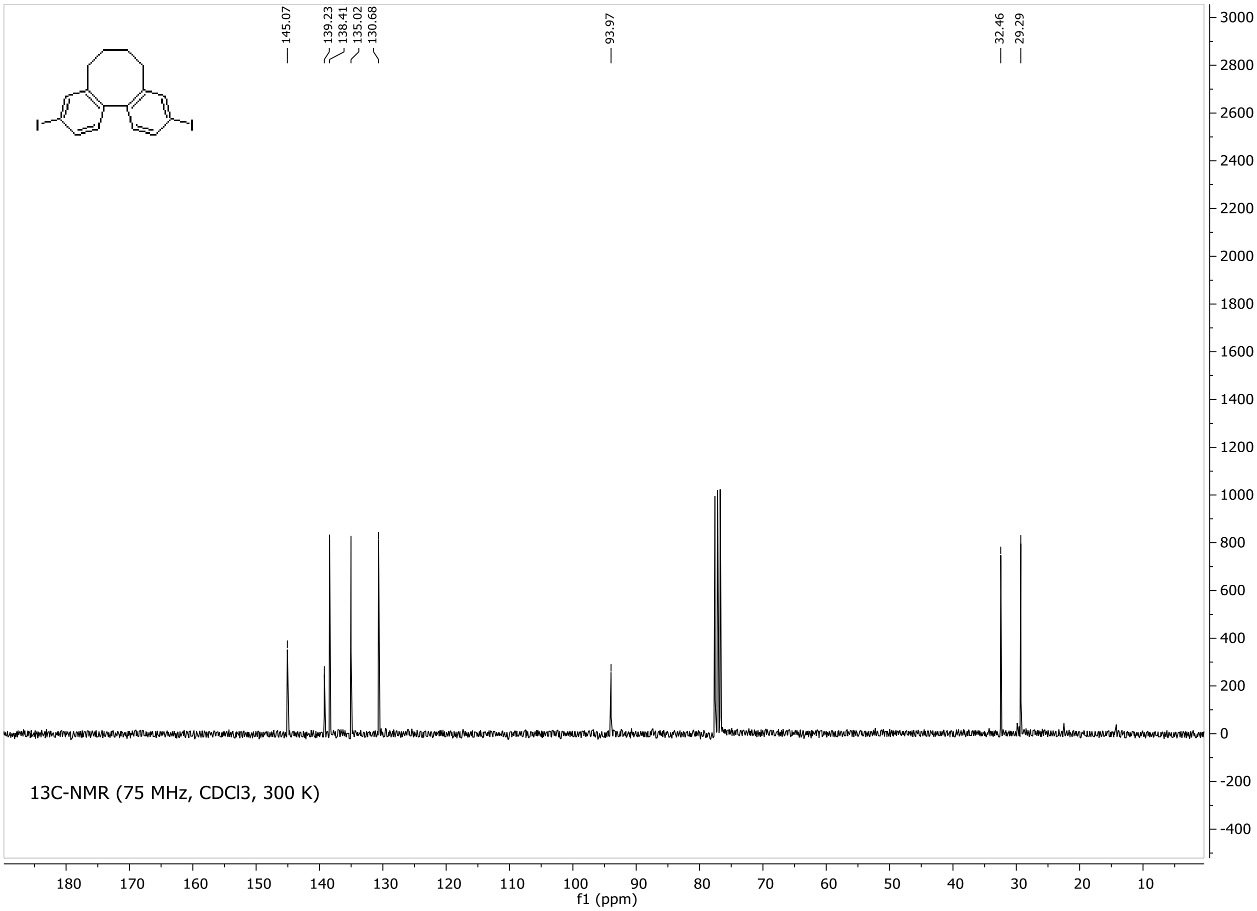
145.07
139.23
138.41
135.02
130.68
93.97
32.46
29.29
13C-NMR (75 MHz, CDCl3, 300 K)
f1 (ppm)

3,3'-Bis(trimethylsilyl)-biphenyl

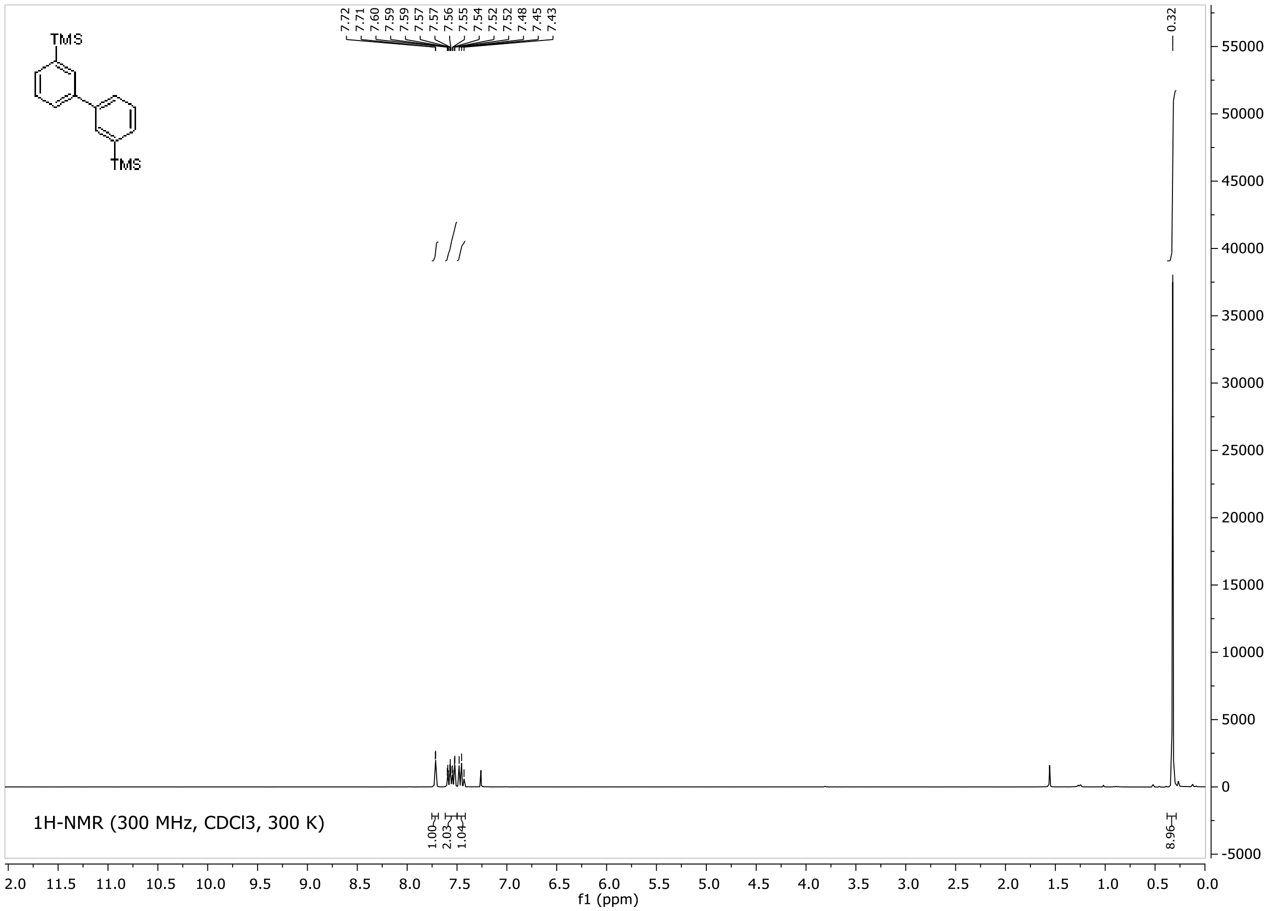

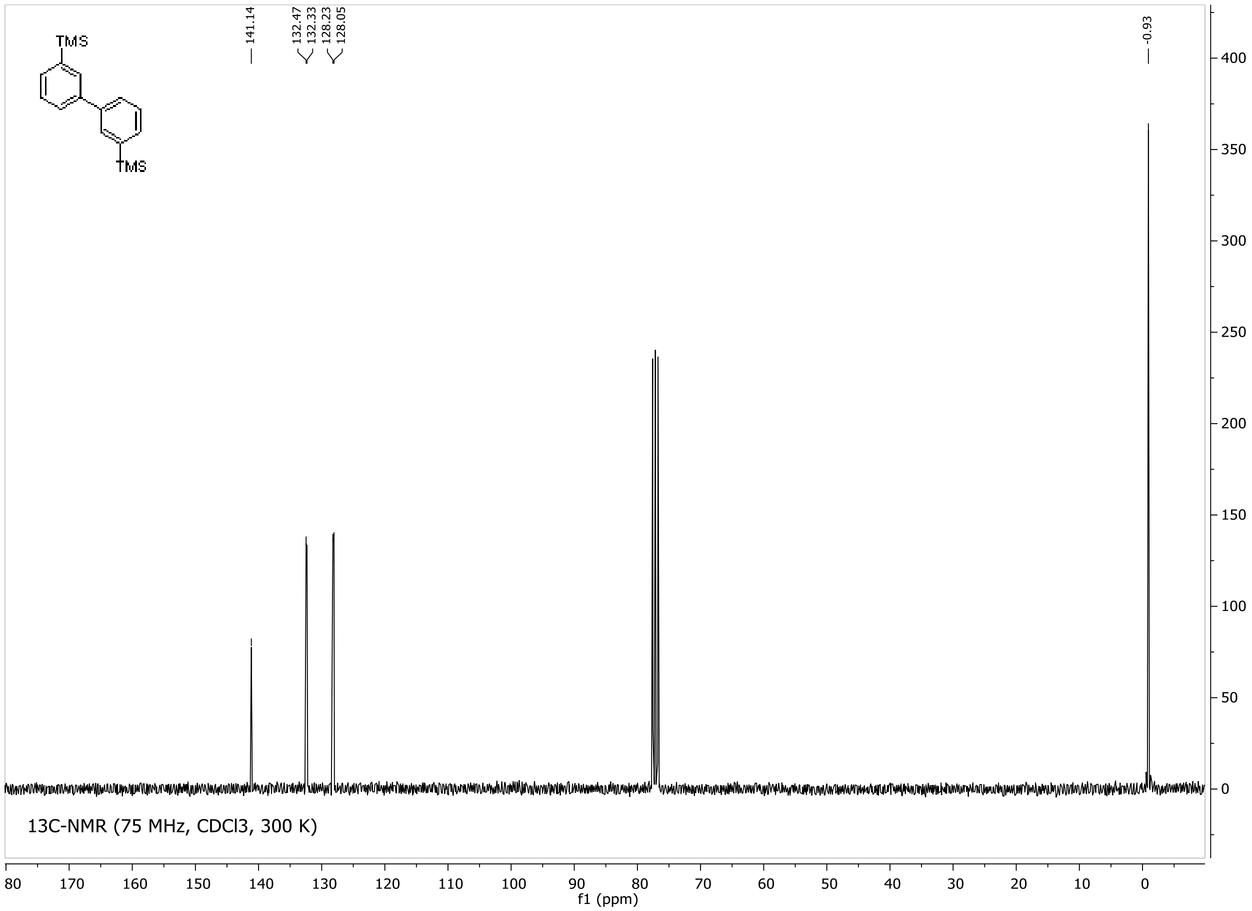
TMS
TMS
141.14
132.47
132.33
128.23
128.05
-0.93
13C-NMR (75 MHz, CDCl3, 300 K)
f1 (ppm)

3,3'-Diiodo-biphenyl (10l)

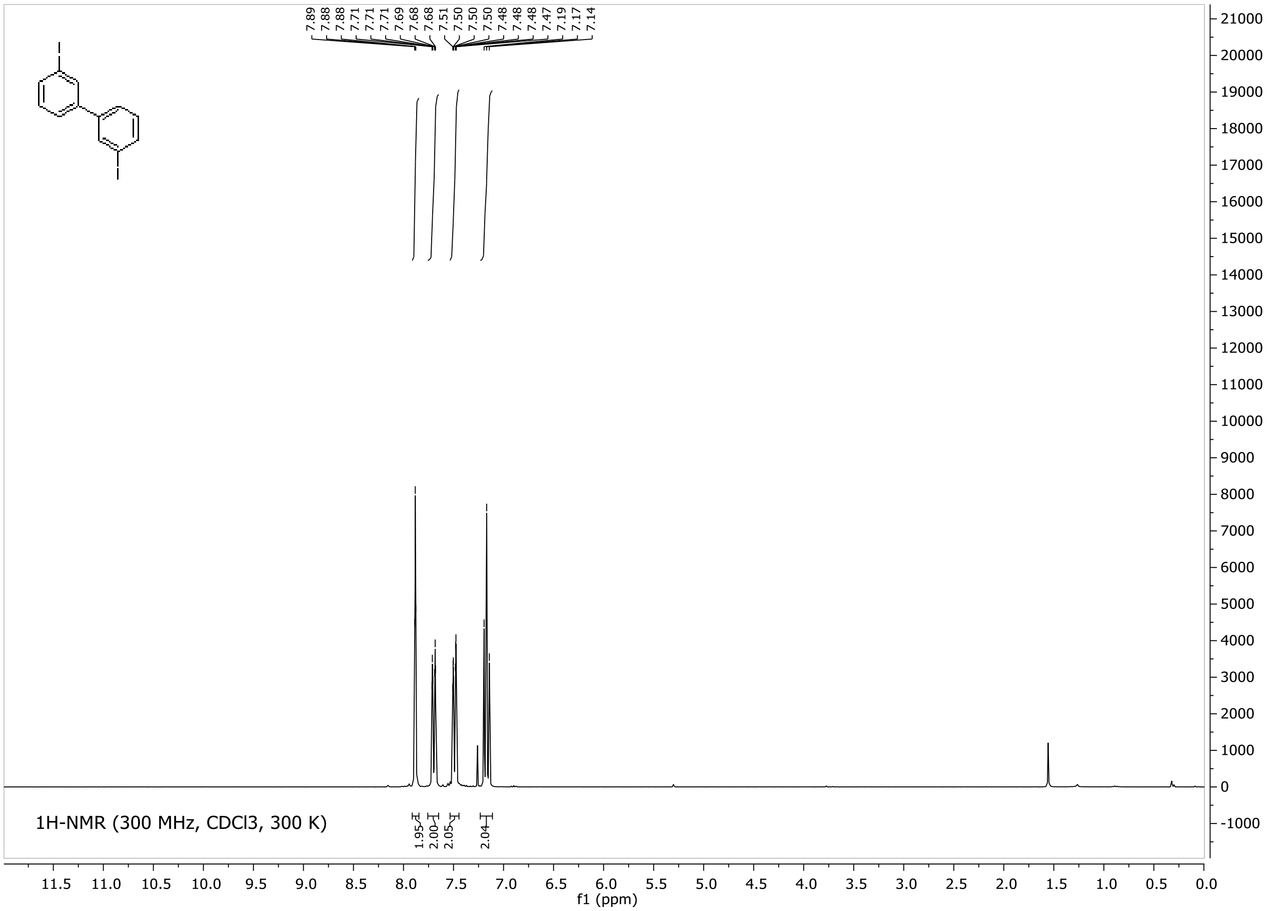

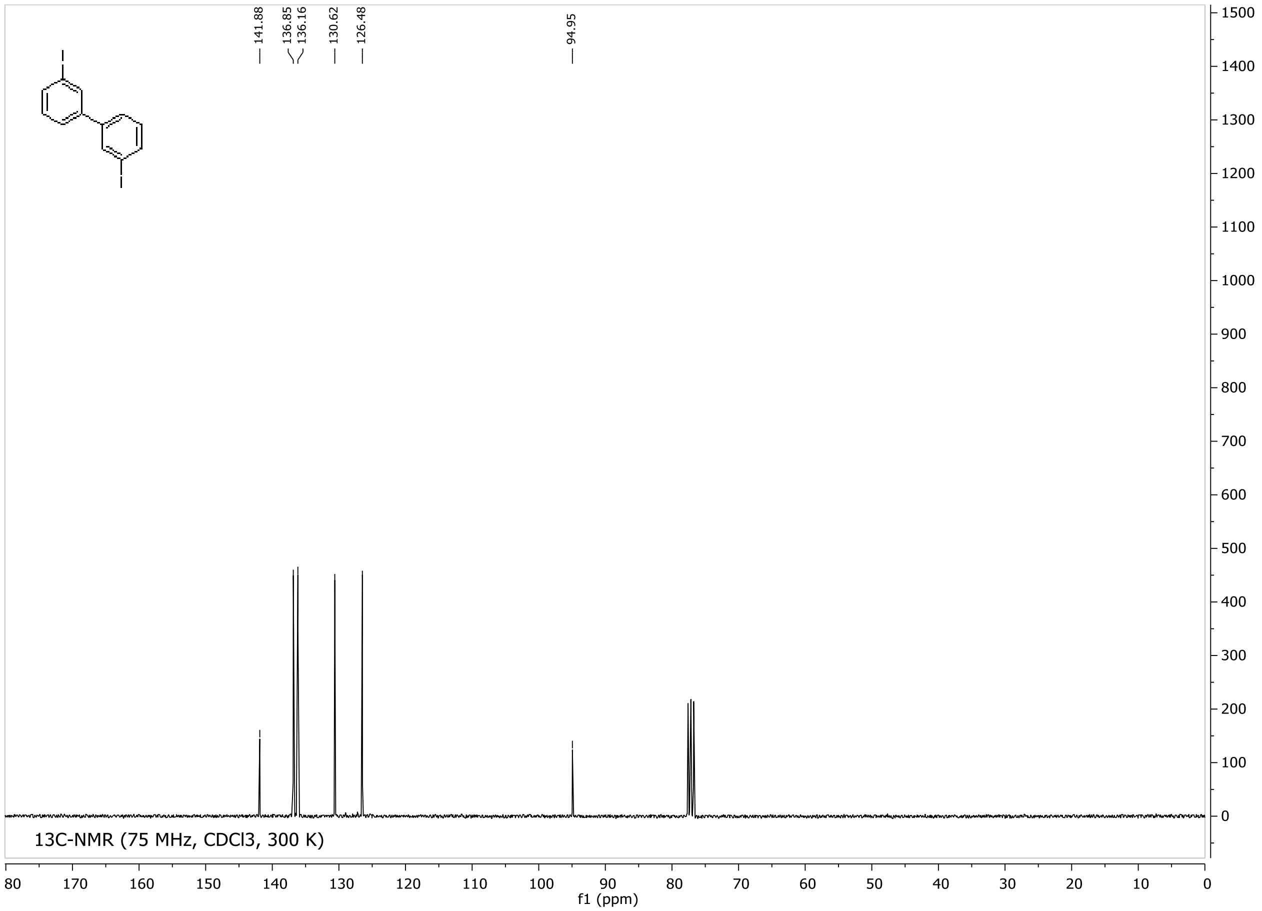

141.88
136.85
136.16
130.62
126.48
94.95
13C-NMR (75 MHz, CDCl3, 300 K)
f1 (ppm)

GC traces and mass spectra analyses of the $S_{RN}1$ substitution reactions

Conversions are shown for all reactions analyzed by GC-FID. They were determined by integration of all peaks in the chromatogram arising from reactions involving the starting material with respect to the carbon number of each individual product. For reactions analyzed by MALDI and other mass spectrometry techniques we were not able to give a quantitative measure for the conversion. The corresponding mass spectra however show a large signal of the starting material to assure that the reaction was not close to completion.

10a

Reaction of **10a** according to general procedure 1. The reaction was stopped after 5 minutes of irradiation (4% conversion; yield of **11a**: 4%) and analyzed by GC-FID (trace shown below). The peaks were assigned by GC-MS (EI).

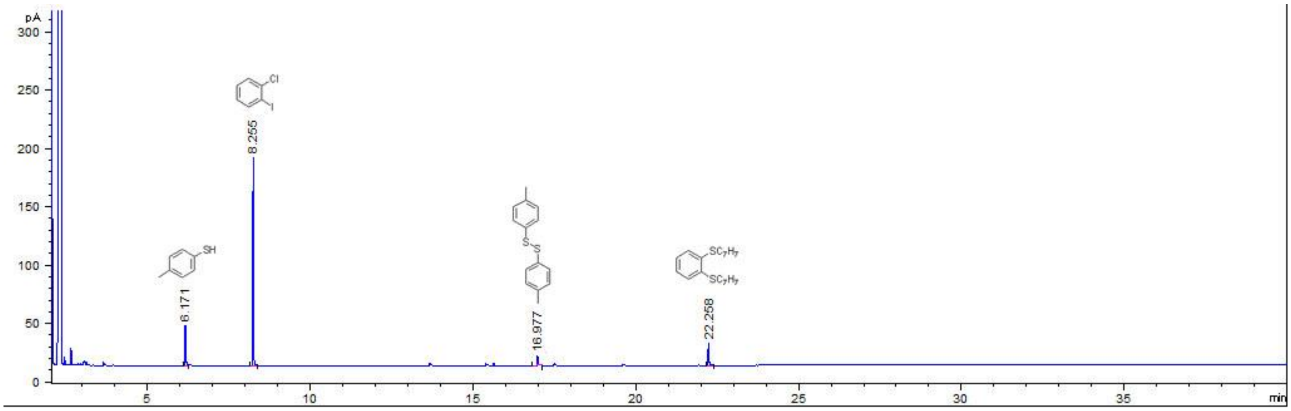

10b

Reaction of **10b** according to general procedure 1. The reaction was stopped after 5 minutes of irradiation (16% conversion; yield of **11b**: 11%) and analyzed by GC-FID (trace shown below). The peaks were assigned by GC-MS (EI).

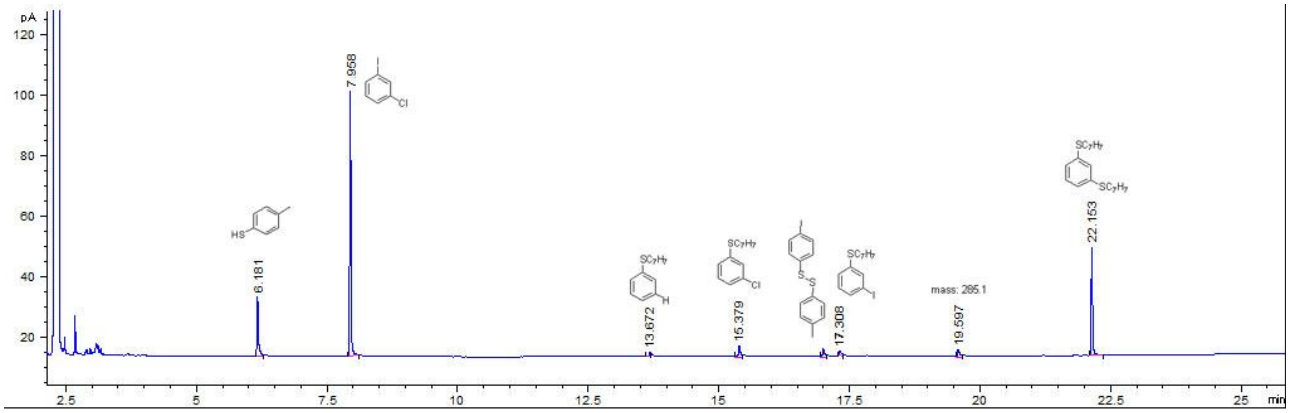

10c

Reaction of **10c** according to general procedure 1. The reaction was stopped after 5 minutes of irradiation (15% conversion; yield of **11c**: 12%) and analyzed by GC-FID (trace shown below). The peaks were assigned by GC-MS (EI).

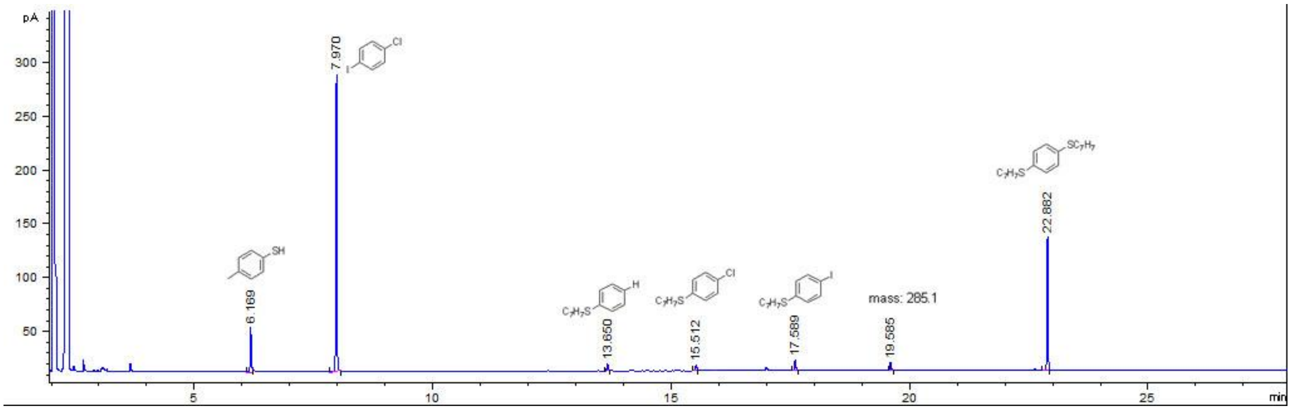

10d

Reaction of **10d** according to general procedure 1. The reaction was stopped after 6 hours of irradiation (48% conversion; yield of **13d**: 21%) and analyzed by GC-FID (trace shown below). The peaks were assigned by GC-MS (EI).

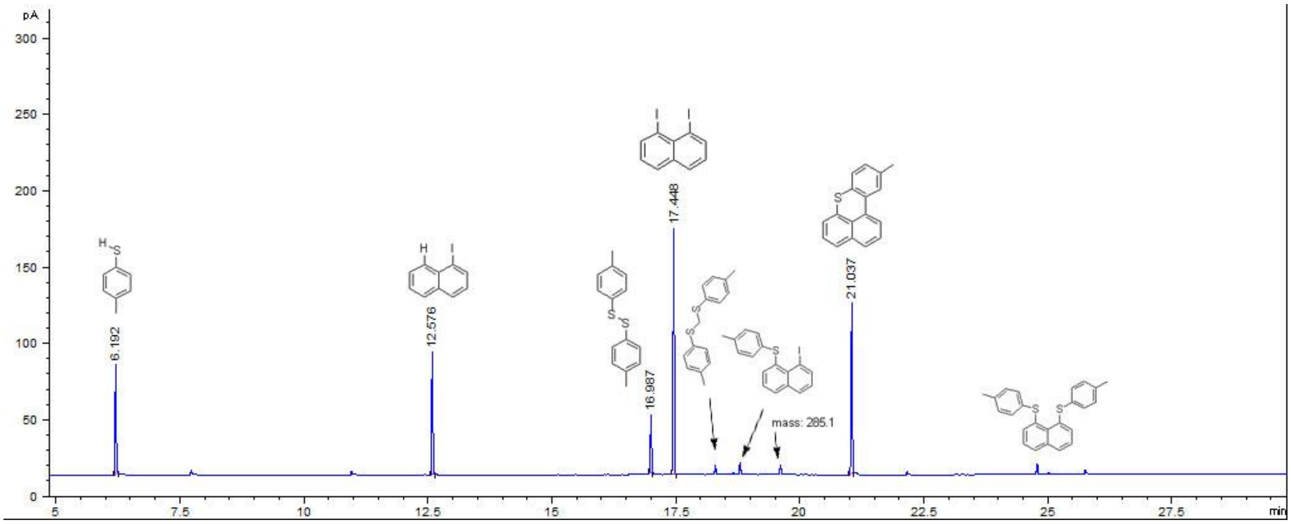

10e

Reaction of **10e** according to general procedure 1. The reaction was stopped after 6 hours of irradiation (15% conversion; yield of **11e**: 10%) and analyzed by GC-FID (trace shown below). The peaks were assigned by GC-MS (EI).

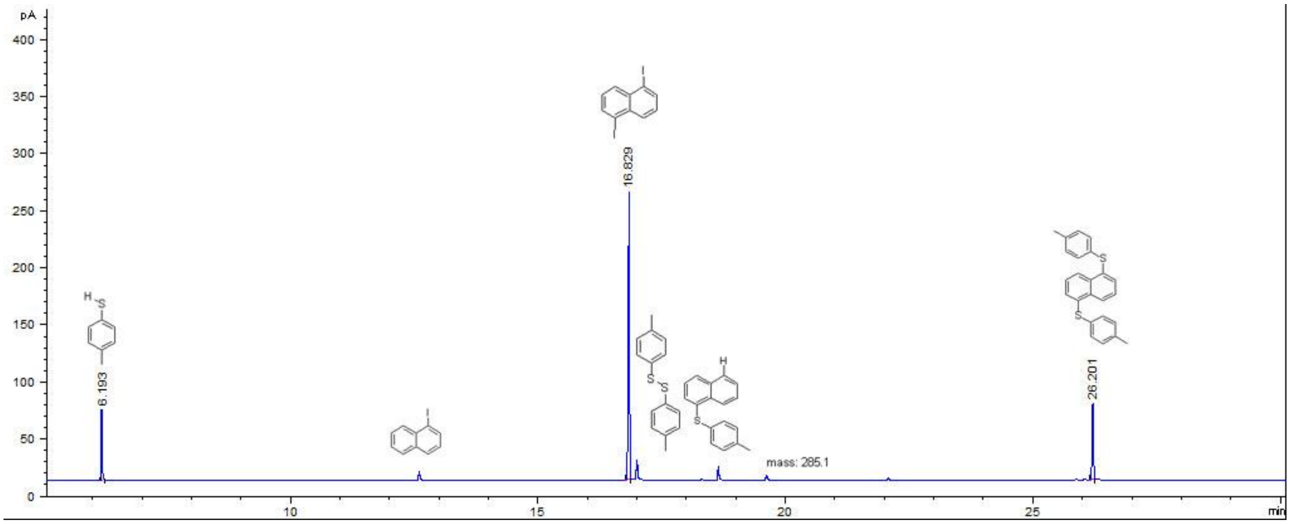

10f

Reaction of **10f** according to general procedure 1. The reaction was stopped after 6 hours of irradiation (27% conversion; yield of **11f**: 20%) and analyzed by GC-FID (trace shown below). The peaks were assigned by GC-MS (EI).

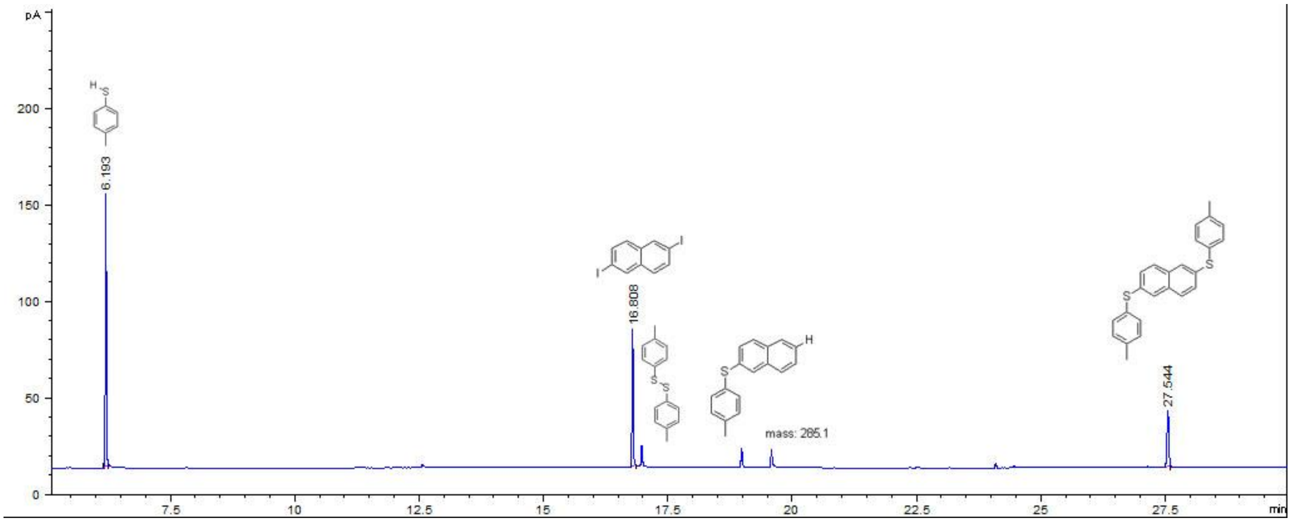

10g

Reaction of **10g** according to general procedure 1. The reaction was stopped after 5 hours of irradiation (41% conversion; yield of **11g**: 33%) and analyzed by GC-FID (trace shown below). The peaks were assigned by GC-MS (EI).

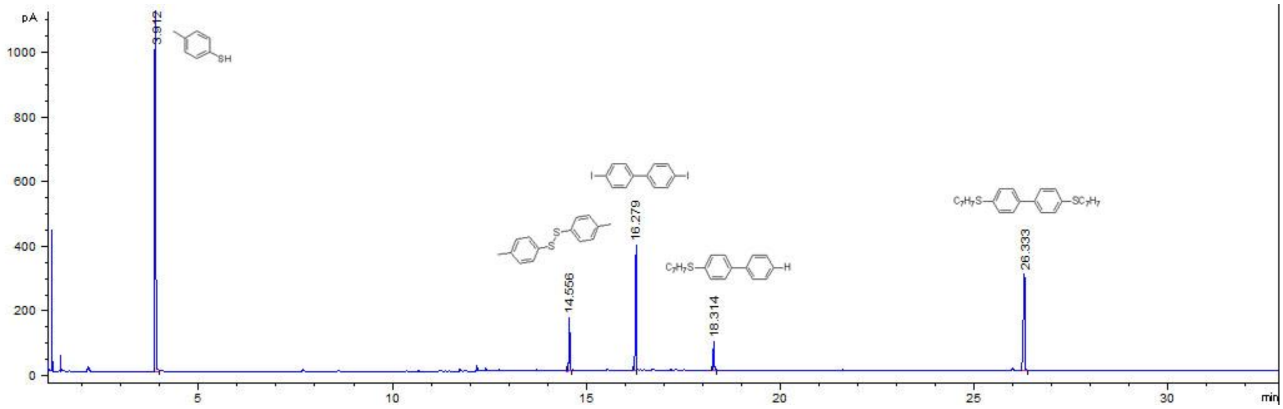

10h

Reaction of **10h** according to general procedure 1. The reaction was stopped after 5 hours of irradiation (80% conversion; yield of **11h**: 6%; the high conversion was necessary due to the high amount of side products arising from dehalogenation) and analyzed by GC-FID (trace shown below). The peaks were assigned by GC-MS (EI).

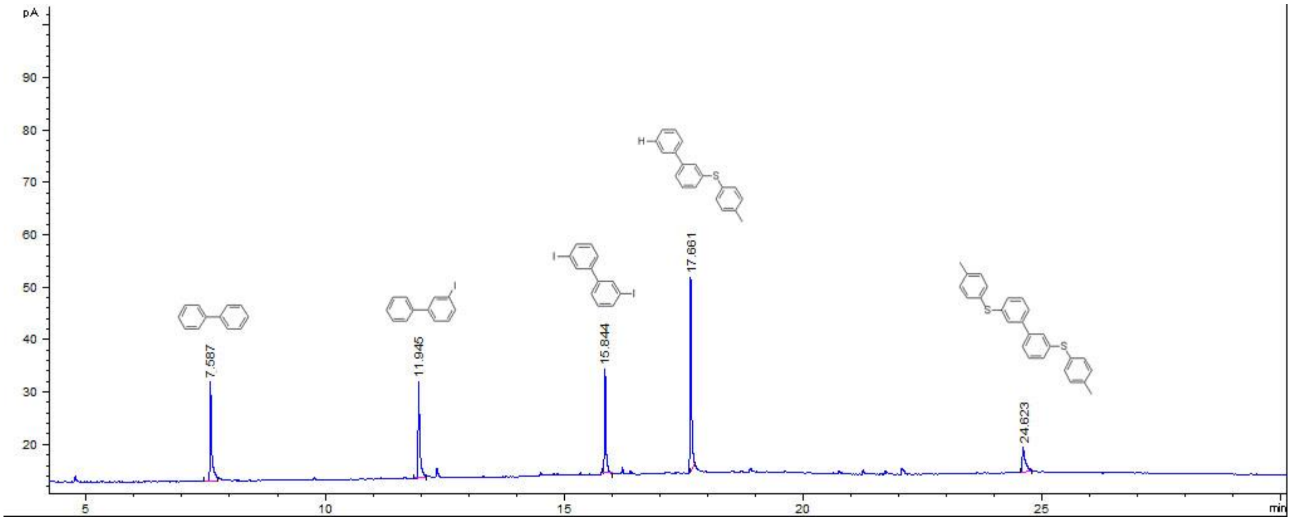

10i

Reaction of **10i** according to general procedure 1. The reaction was stopped after 8 hours of irradiation (45% conversion; yield **11i**: 2%; yield of **12i**: 3%) and analyzed by GC-FID (trace shown below). The peaks were assigned by GC-MS (EI).

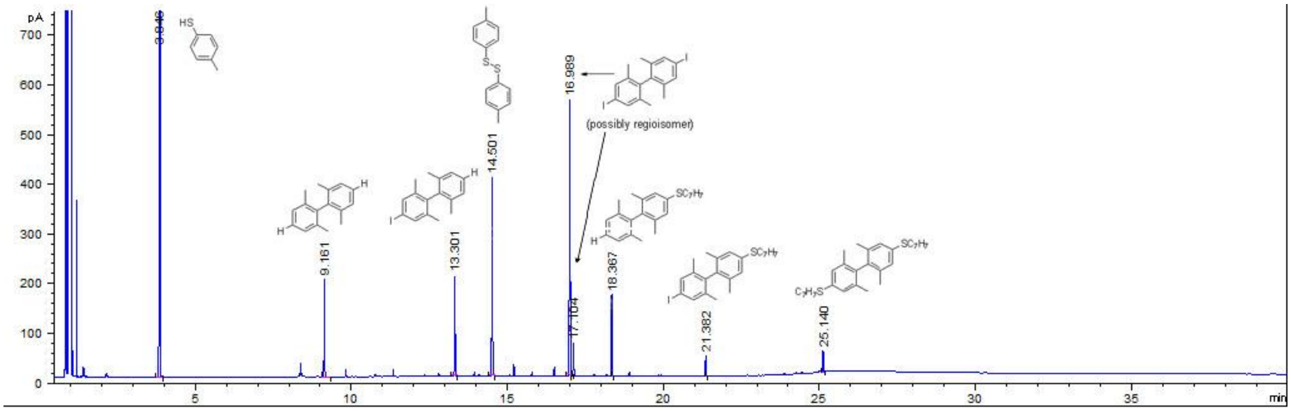

Since this experiment yielded both, mono and bis substituted product, we investigated the influence of the concentration of the substrate on the ratio of mono versus bis substitution. The diagram below shows a linear correlation of concentration to ratio of bis/mono supporting our claim of competition between intra- and inter-molecular ET in this case.

c (M)	Ratio (bis/mono)
0,05	1,00
0,1	0,90
0,15	0,72
0,2	0,61

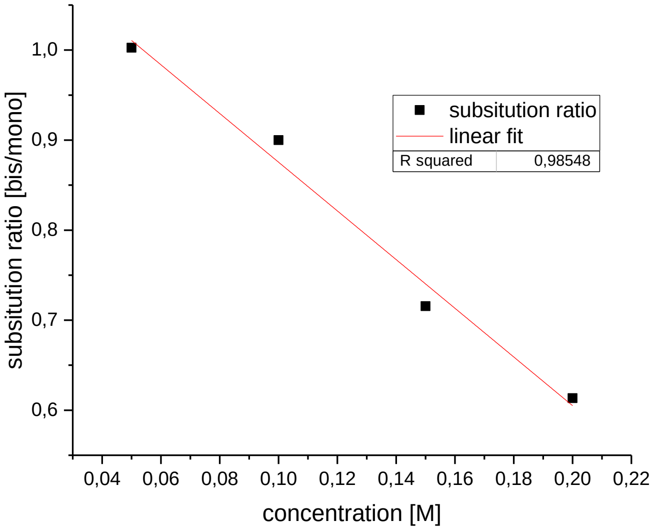

10j

Reaction of **10j** according to general procedure 1. The reaction was stopped after 16 hours of irradiation (47% conversion; yield **11jh**: 19%) and analyzed by GC-FID (trace shown below). The peaks were assigned by GC-MS (EI).

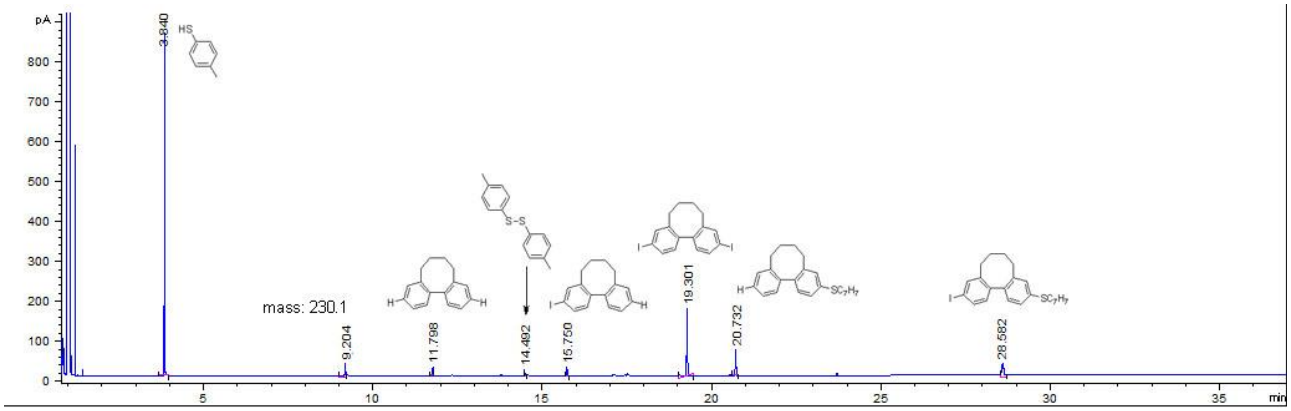

10k

Reaction of **10k** according to general procedure 1. The reaction was stopped after 1 hour of irradiation and analyzed by MS (APCI). The mass of the product of mono substitution (m/z = 638.2443) was not detected.

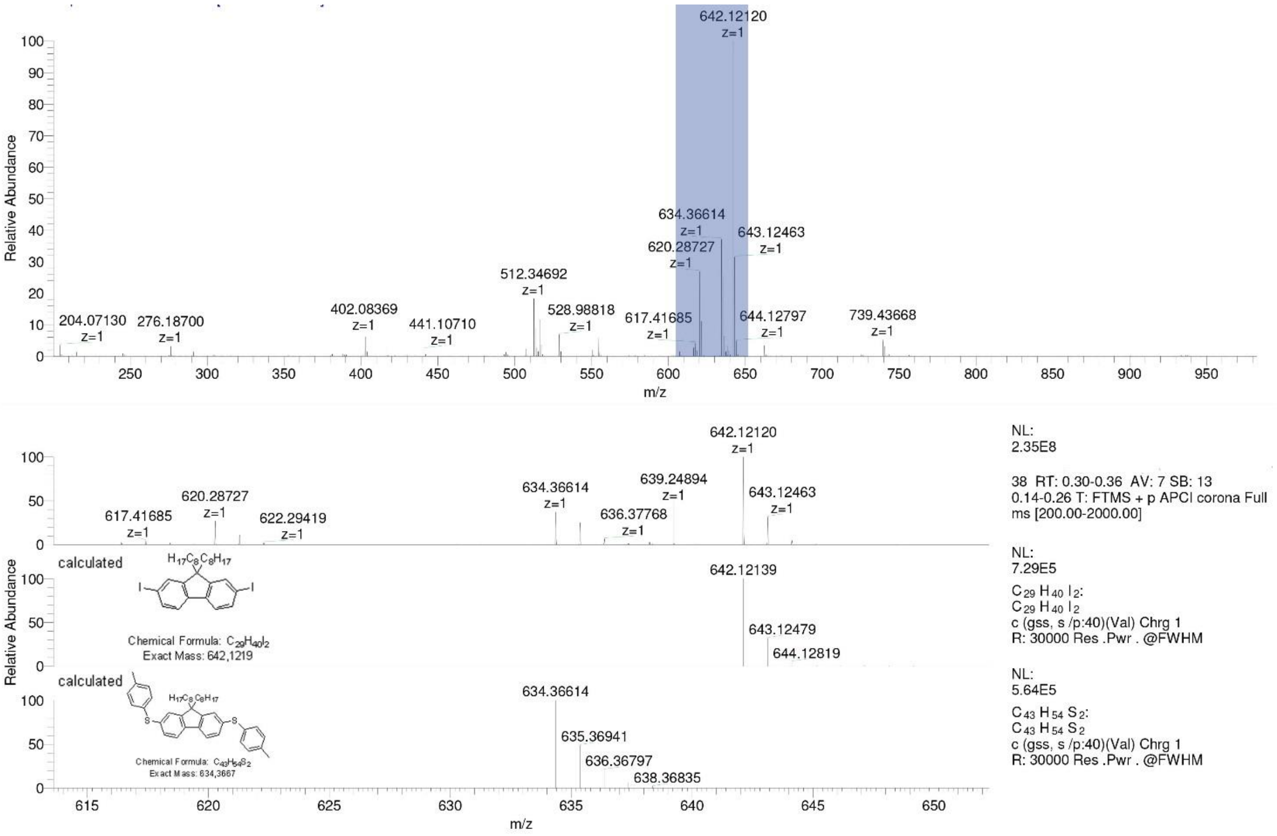

10l

Reaction of **10l** according to general procedure 1. The reaction was stopped after 1 hour of irradiation and analyzed by MS (APCI). The mass of the product of mono substitution (**11l**, m/z = 527.1144 for [$C_{27}H_{30}INS$]; m/z = 634.0195 for [M+Ag]; m/z = 757.0463 for [M+AgSC$_7$H$_7$]) was not detected.

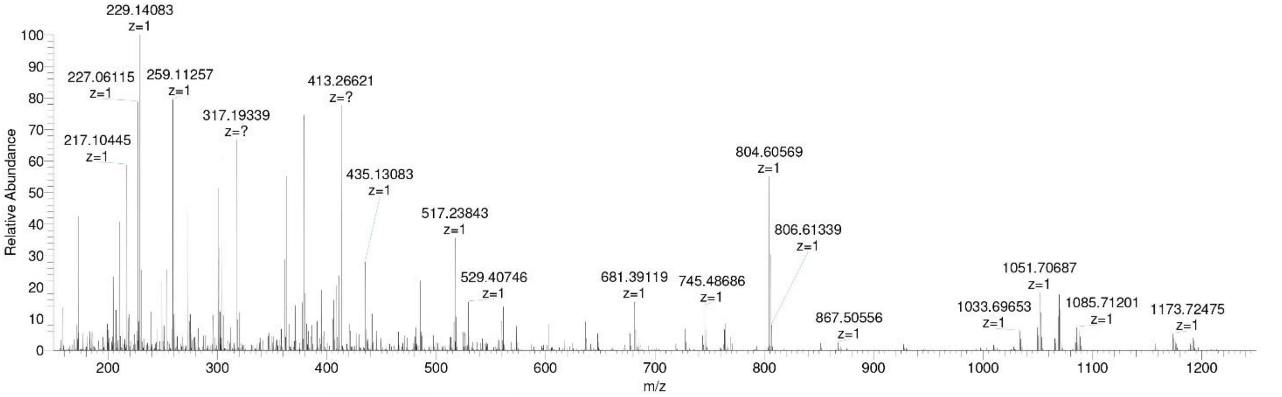

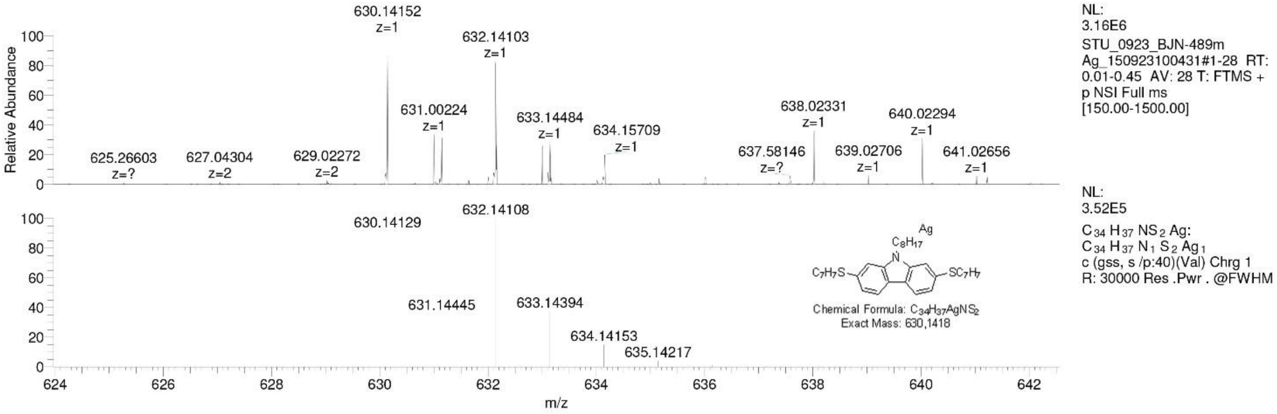

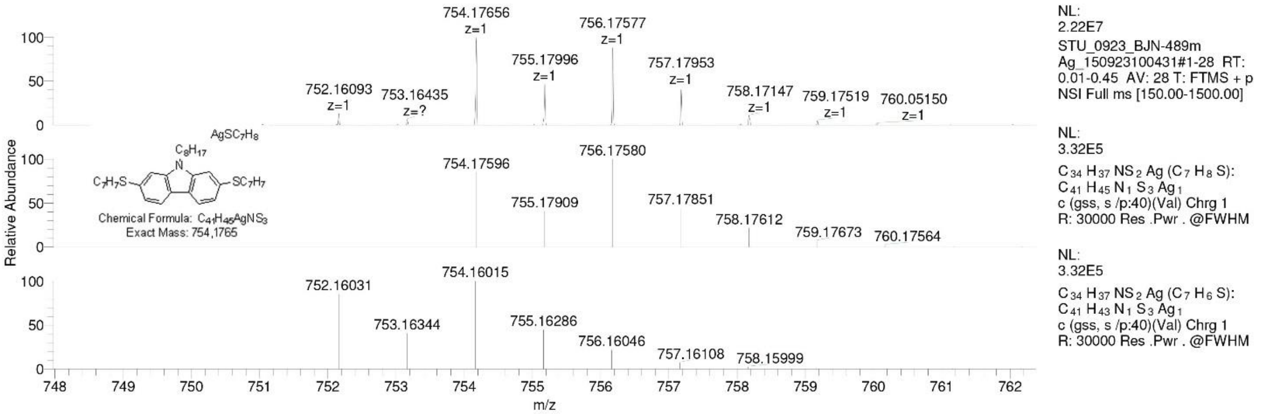

10m

Reaction of **10m** according to general procedure 1. The reaction was stopped after 6 hours of irradiation (40% conversion; yield **11m**: 33%) and analyzed by GC-FID (trace shown below). The peaks were assigned by GC-MS (EI).

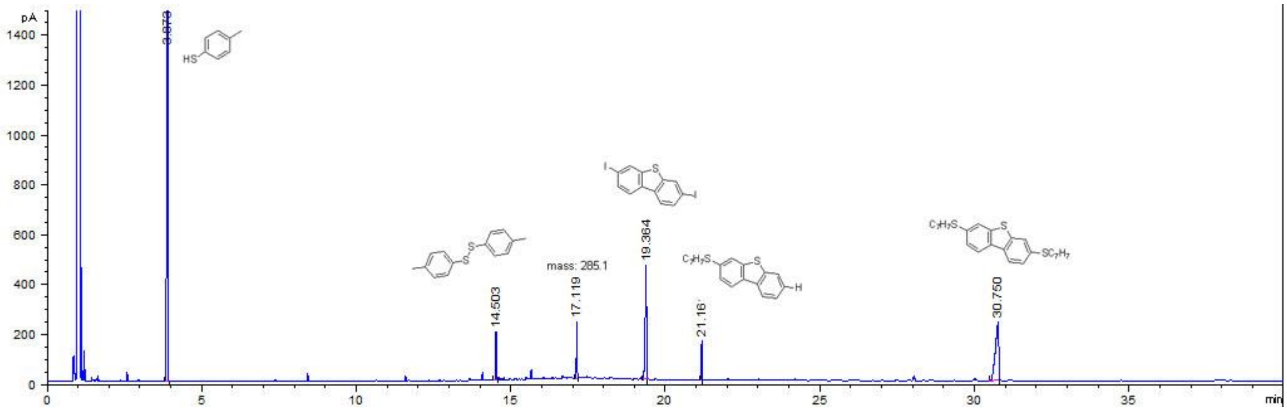

10n

Reaction of **10n** according to general procedure 1. The reaction was stopped after 6 hours of irradiation (19% conversion; yield of **12n**: 5%) and analyzed by GC-FID (trace shown below). The peaks were assigned by GC-MS (EI).

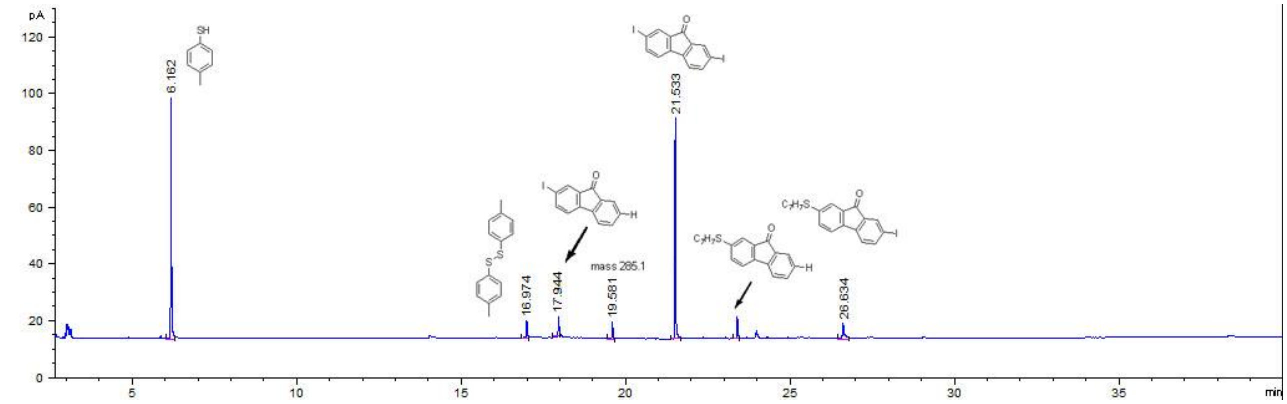

10o

Reaction of **10o** according to general procedure 1. The reaction was stopped after 6 hours of irradiation (47% conversion; yield **11o**: 5%; yield **12o**: 11%) and analyzed by GC-FID (trace shown below). The peaks were assigned by GC-MS (EI).

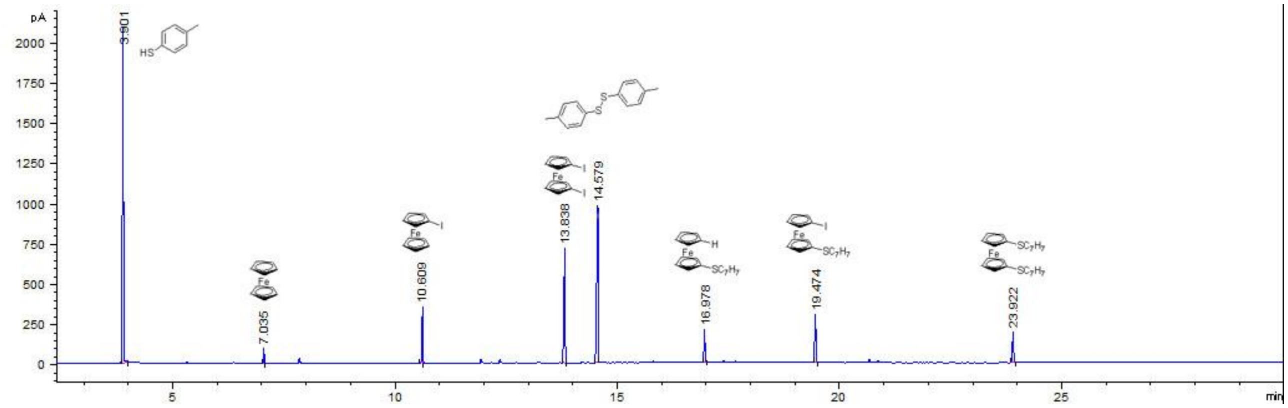

10p

Reaction of **10p** according to general procedure 1. The reaction was stopped after 7 hours and analyzed by MALDI-MS. The peak detected at m/z = 472.27 is caused by the matrix material (DCTB). The product caused by mono substitution (**11p**, m/z = 478.0252) was not detected. The starting material does not show up in MALDI but was detected in APCI measurements.

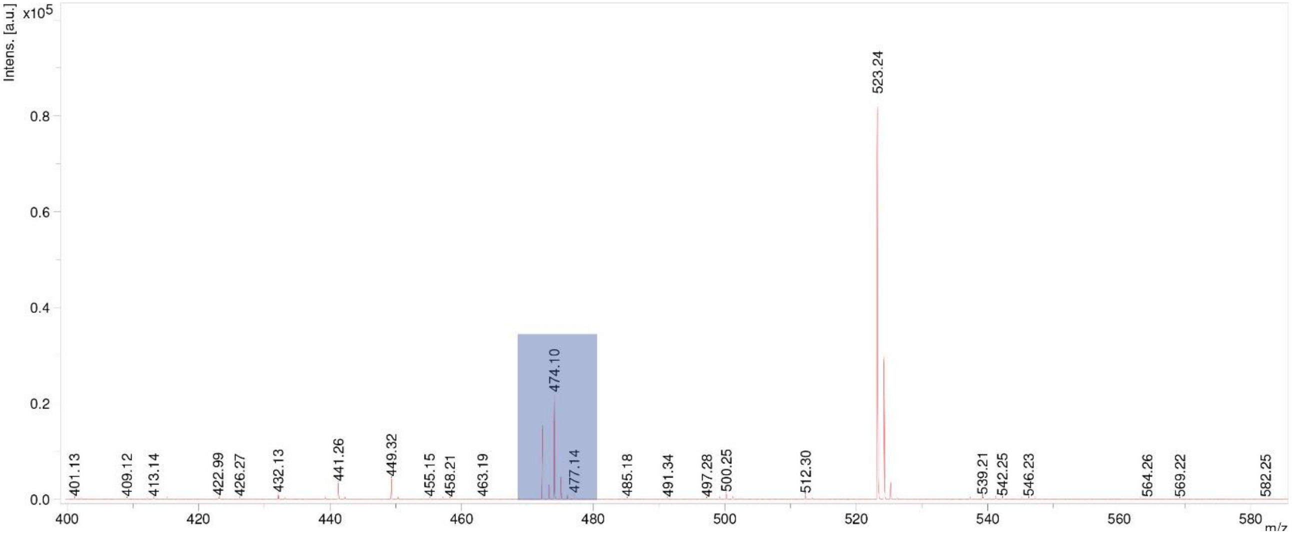

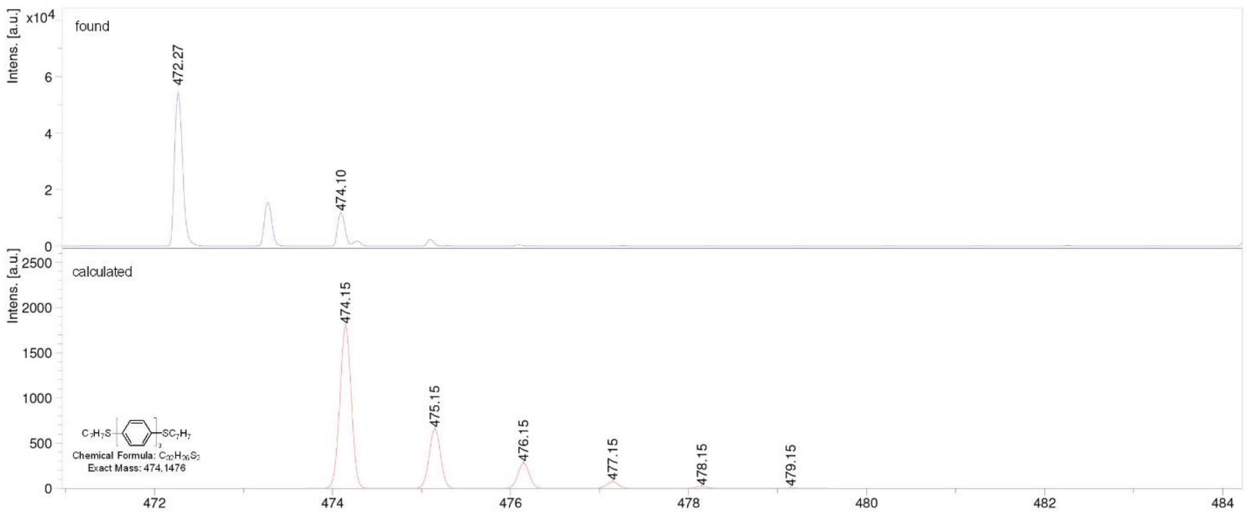

10q

Reaction of **10q** according to general procedure 1. The reaction was stopped after 7 hours and analyzed by MALDI-MS. The peak detected at m/z = 472.27 is caused by the matrix material (DCTB). The product caused by mono substitution (**11q**, m/z = 478.0252) was not detected. The starting material does not show up in MALDI but was detected in APCI measurements.

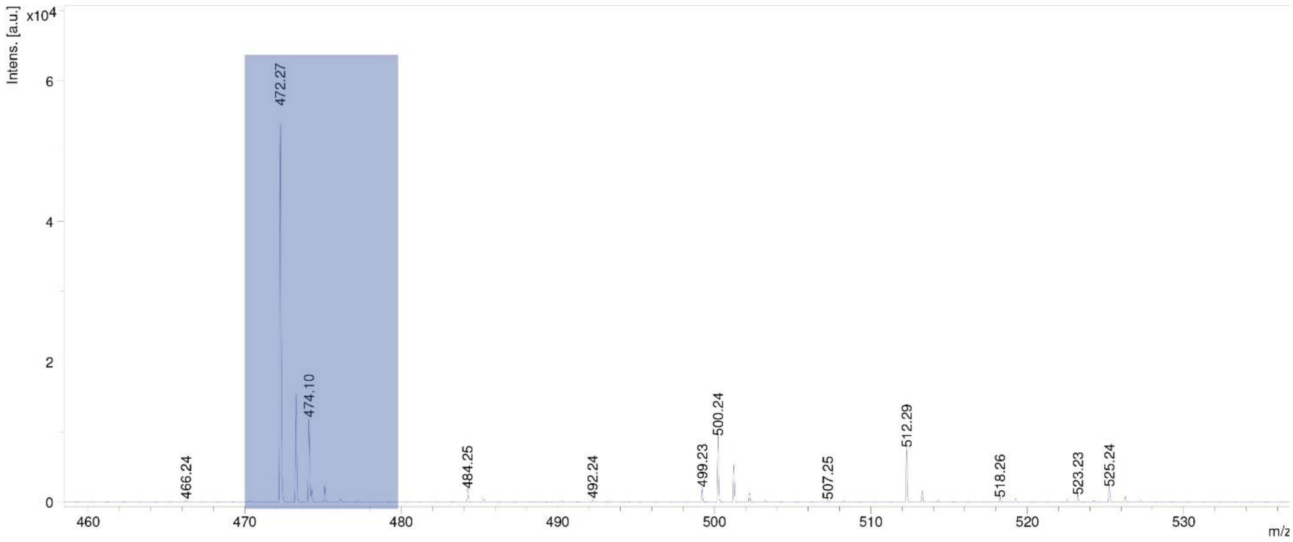

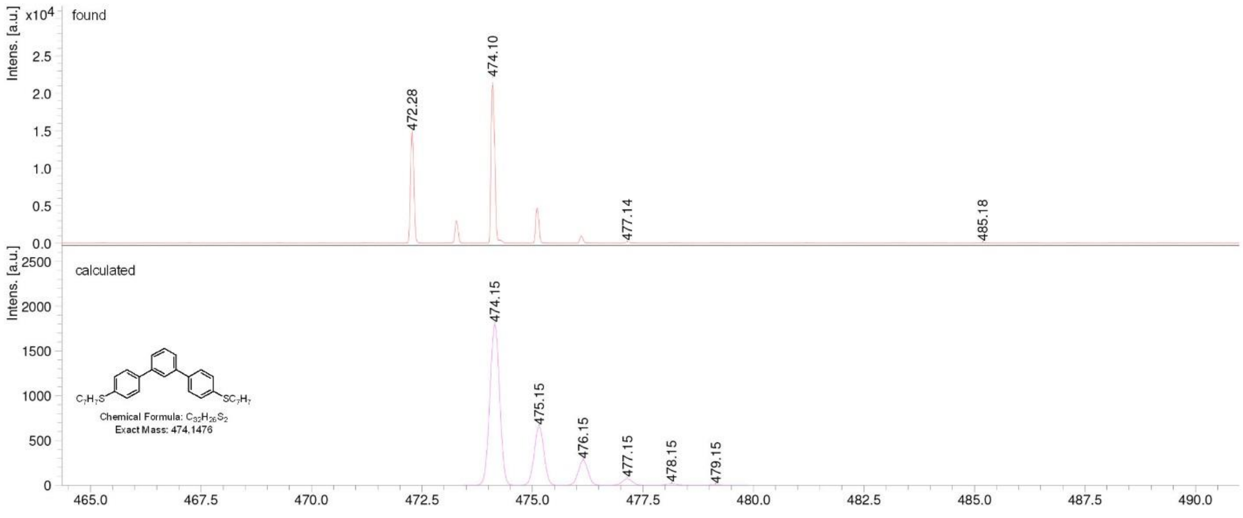

10r

Reaction of **10r** according to general procedure 1. The reaction was stopped after 7 hours and analyzed by MALDI-MS. The products caused by mono substitution (**11r**, m/z = 679.9532) as well as double substitution (**12r**, m/z = 676.0755) were not detected. The starting material does not show up in MALDI but was detected in APCI measurements.

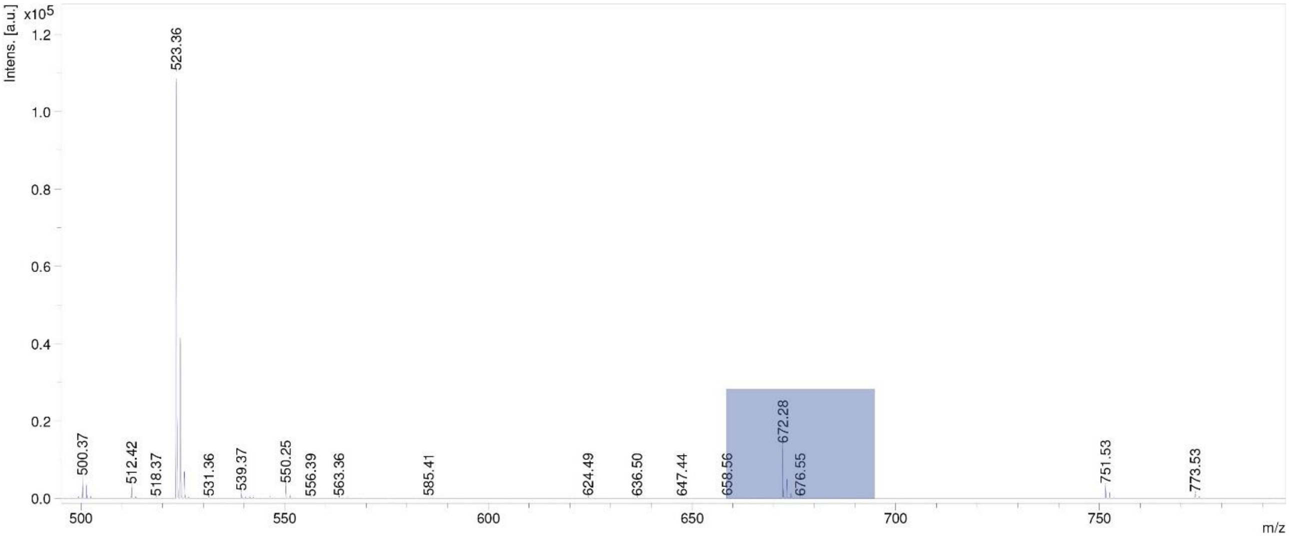

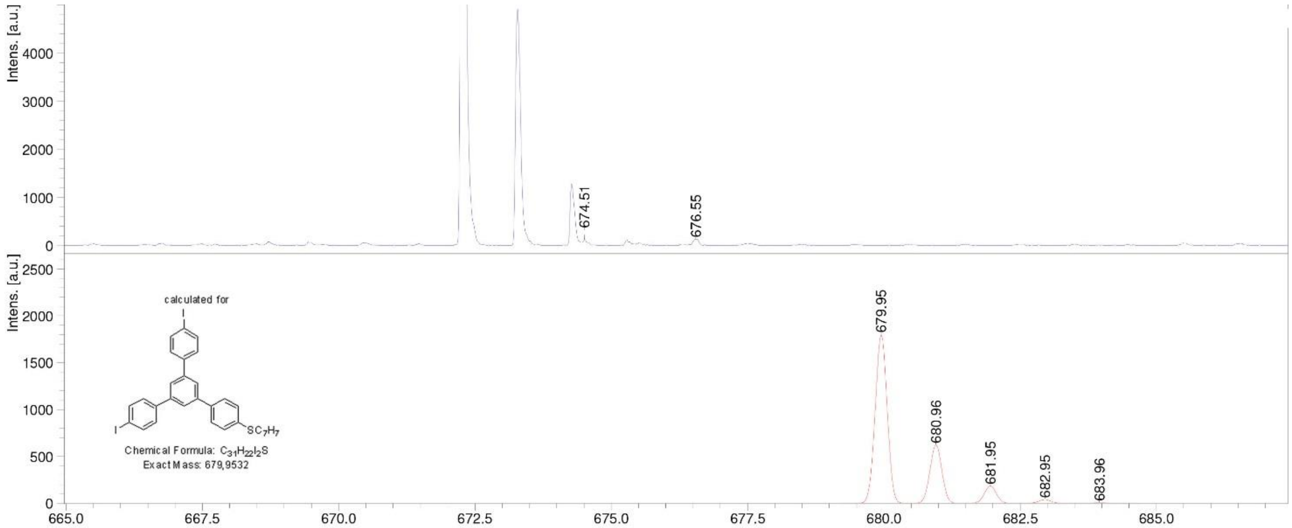
Intens. [a.u.]
674.51
676.55
calculated for
SC7H7
Chemical Formula: C31H22I2S
Exact Mass: 679,9532
679.95
680.96
681.95
682.95
683.96
665.0
667.5
670.0
672.5
675.0
677.5
680.0
682.5
685.0

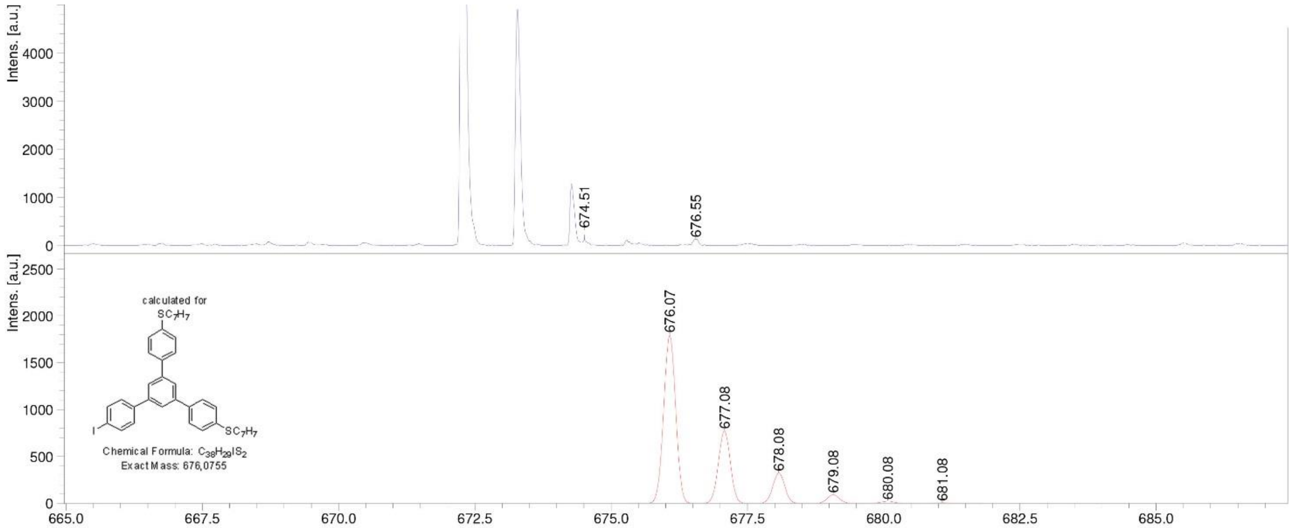
Intens. [a.u.]
674.51
676.55
calculated for
SC7H7
SC7H7
Chemical Formula: C38H29IS2
Exact Mass: 676,0755
676.07
677.08
678.08
679.08
680.08
681.08
665.0
667.5
670.0
672.5
675.0
677.5
680.0
682.5
685.0

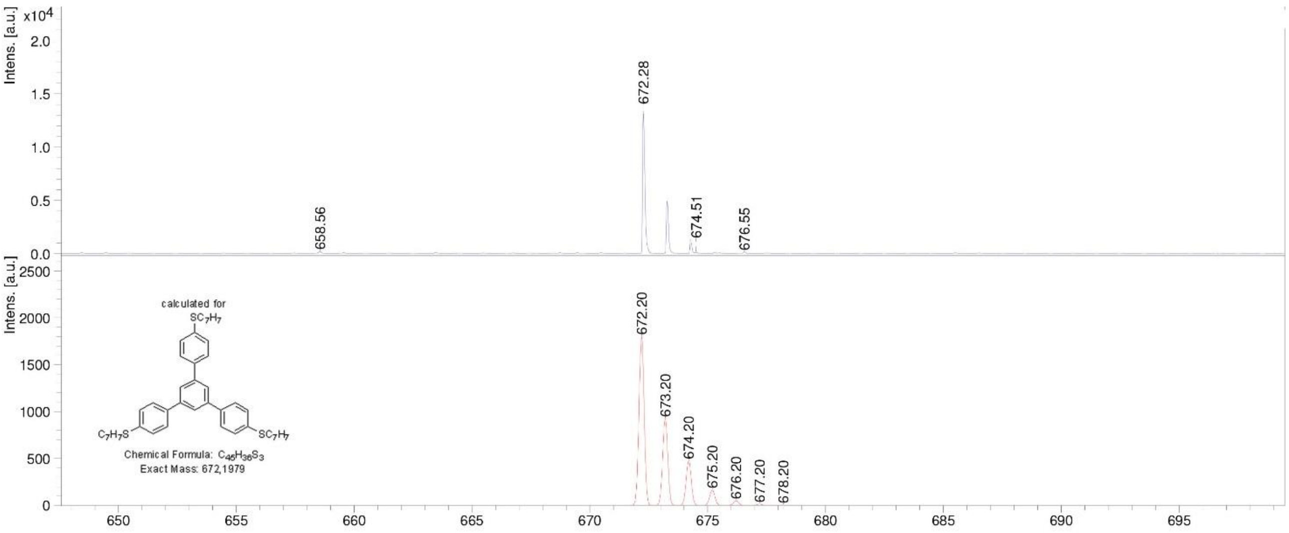

Intens. [a.u.]
x10^4
2.0
1.5
1.0
0.5
0.0
672.28
658.56
674.51
676.55
Intens. [a.u.]
2500
2000
1500
1000
500
0
calculated for
SC7H7
C7H7S
SC7H7
Chemical Formula: C45H36S3
Exact Mass: 672,1979
672.20
673.20
674.20
675.20
676.20
677.20
678.20
650
655
660
665
670
675
680
685
690
695

10s

Reaction of **10s** according to general procedure 1. The reaction was stopped after 8 hours and analyzed by MS (APCI). The product caused by mono substitution (**11s**, m/z = 1026.5573) was not detected.

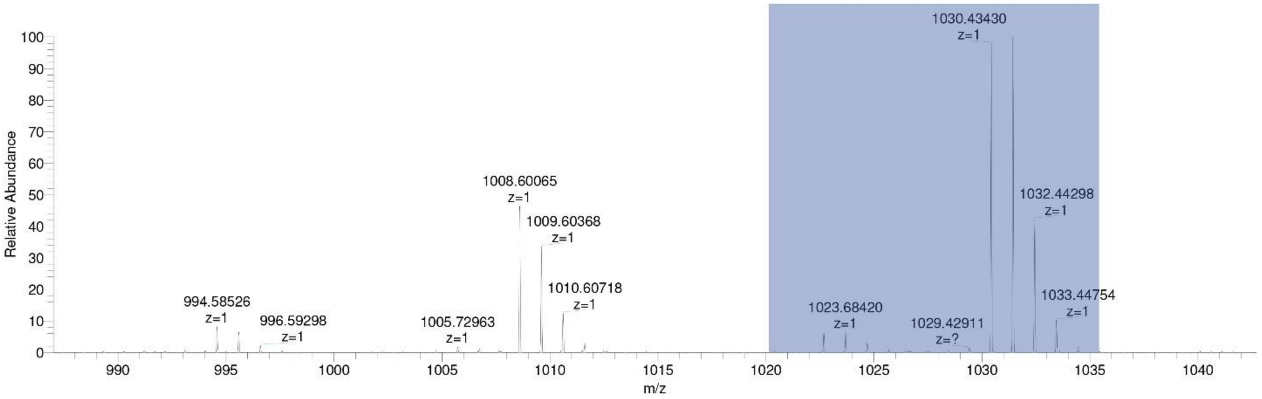

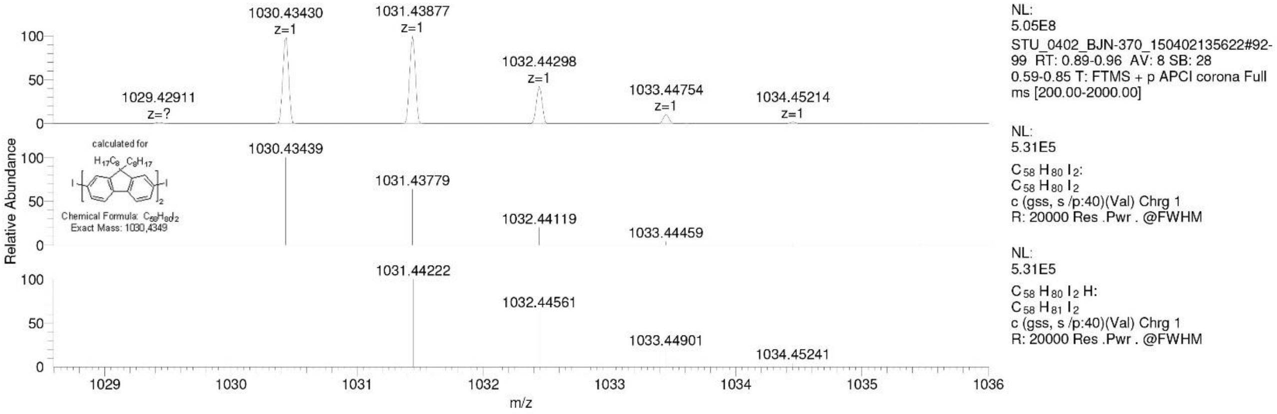

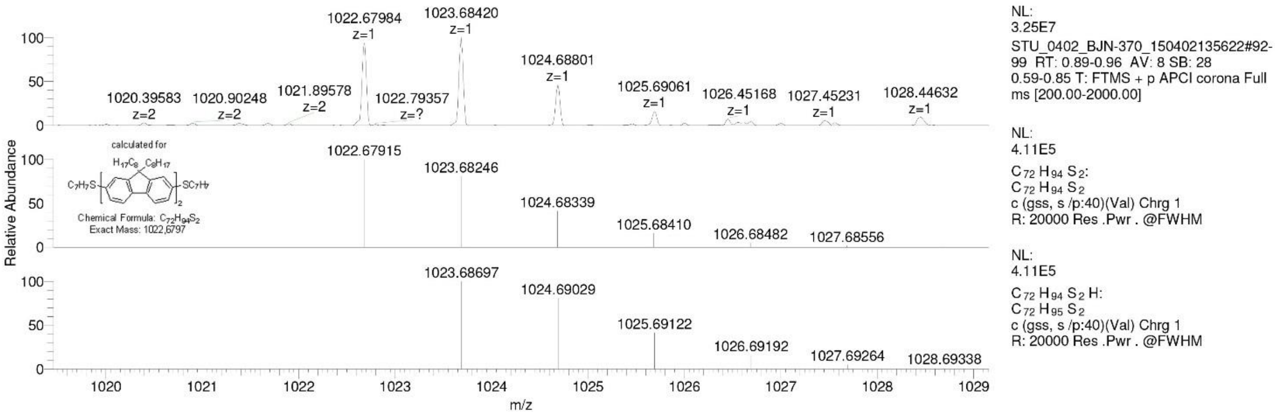

10t

Reaction of **10u** according to general procedure 2. The reaction was stopped after 16 hours and analyzed by MALDI-MS. The product caused by mono substitution (**11t**, m/z = 1414.8703) was not detected.

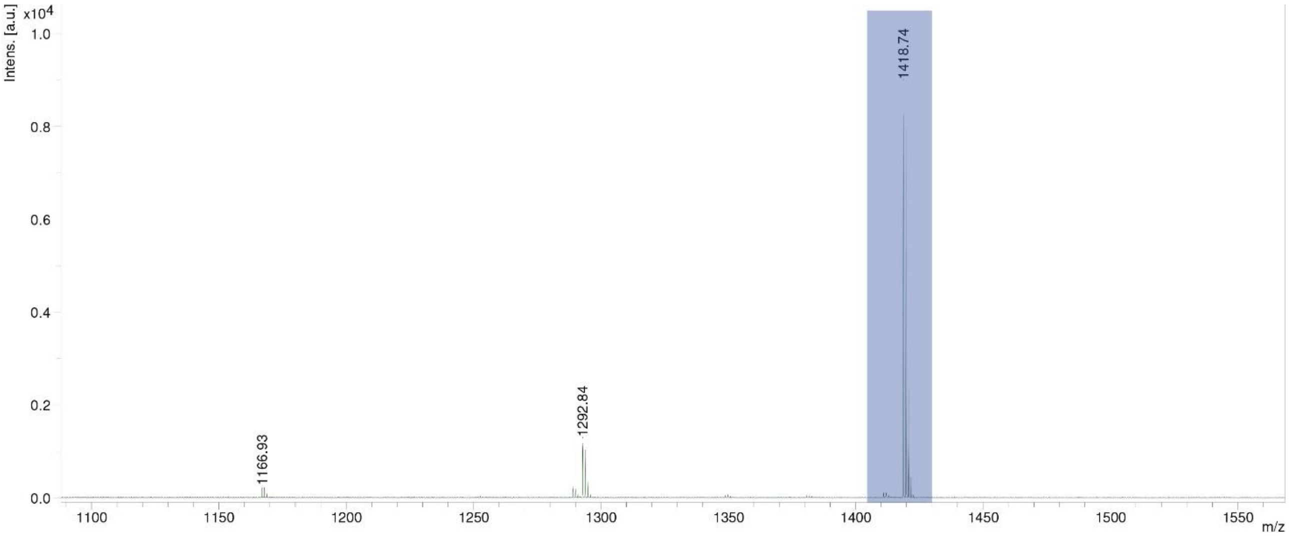

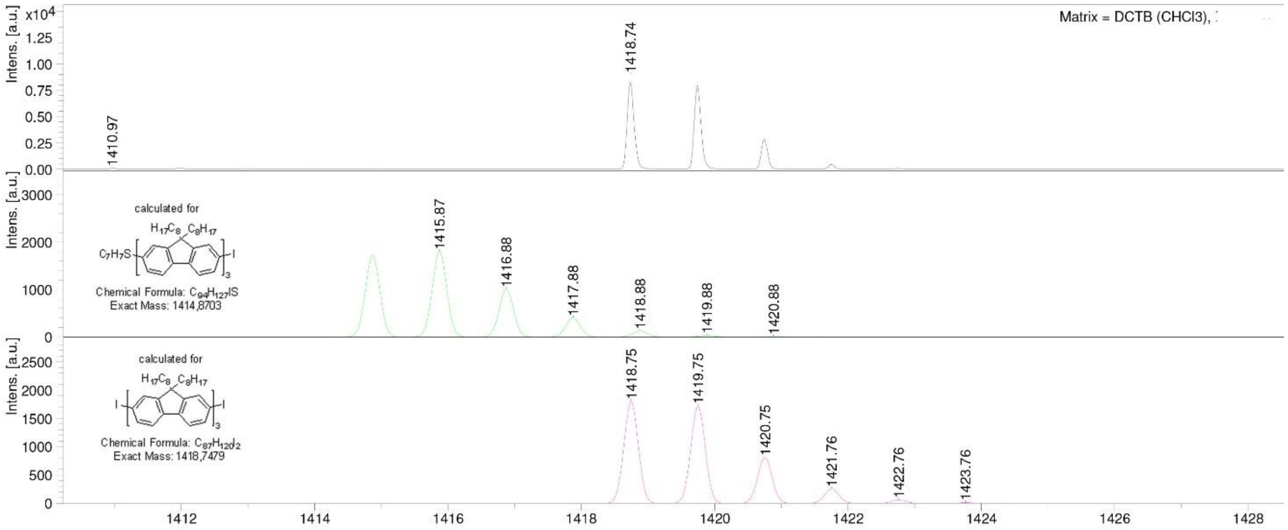

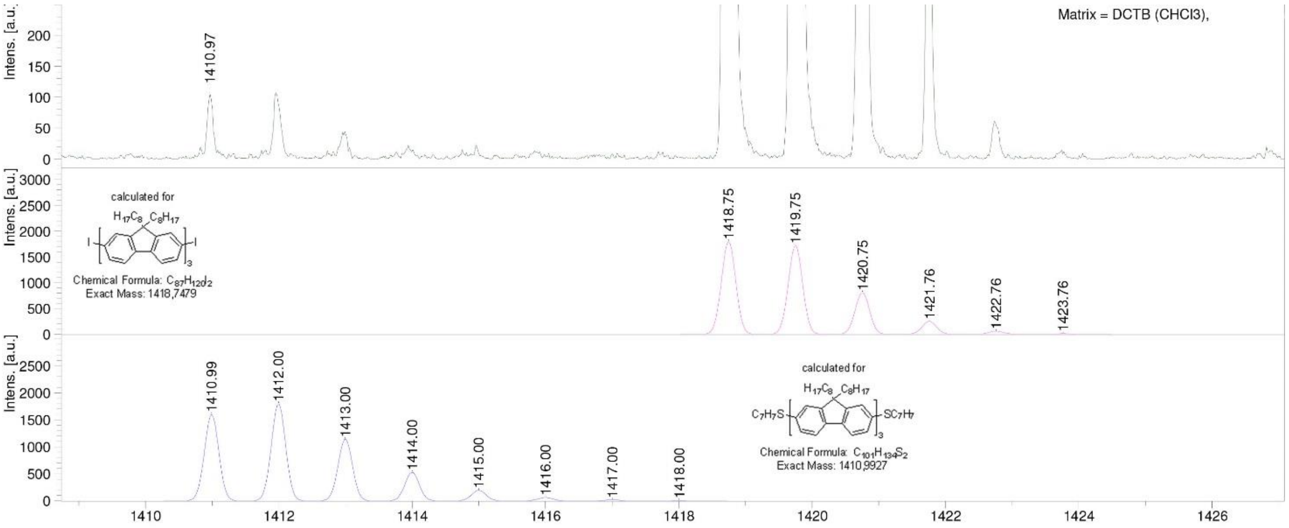

10u

Reaction of **10u** according to general procedure 2. The reaction was stopped after 16 hours and analyzed by MALDI-MS. The product caused by mono substitution (**11u**, m/z = 1803.1833) was not detected.

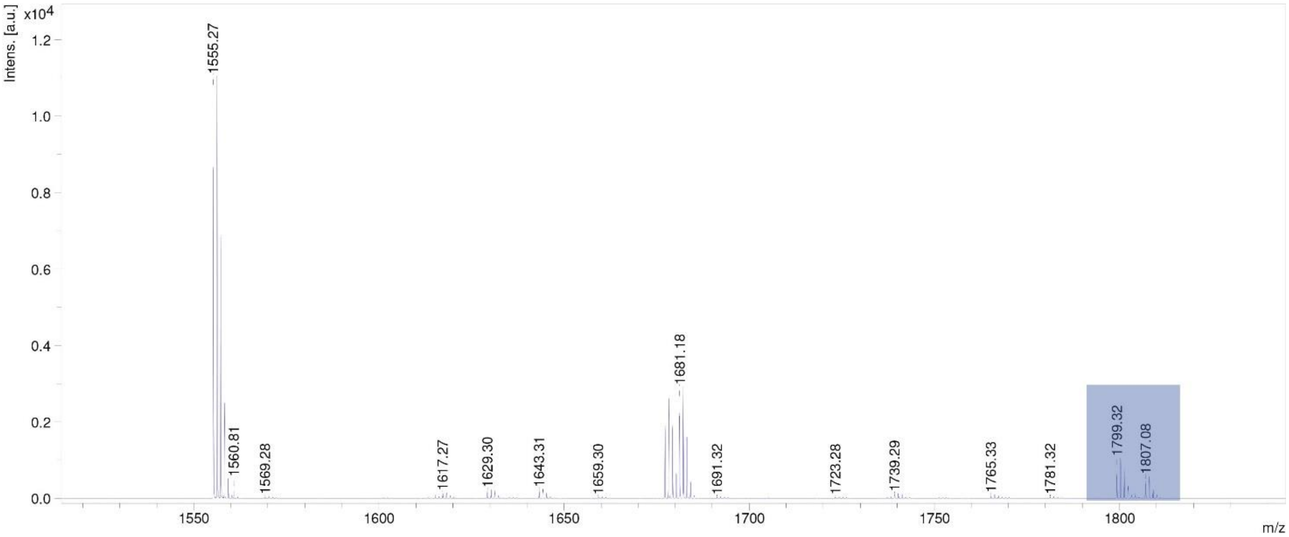

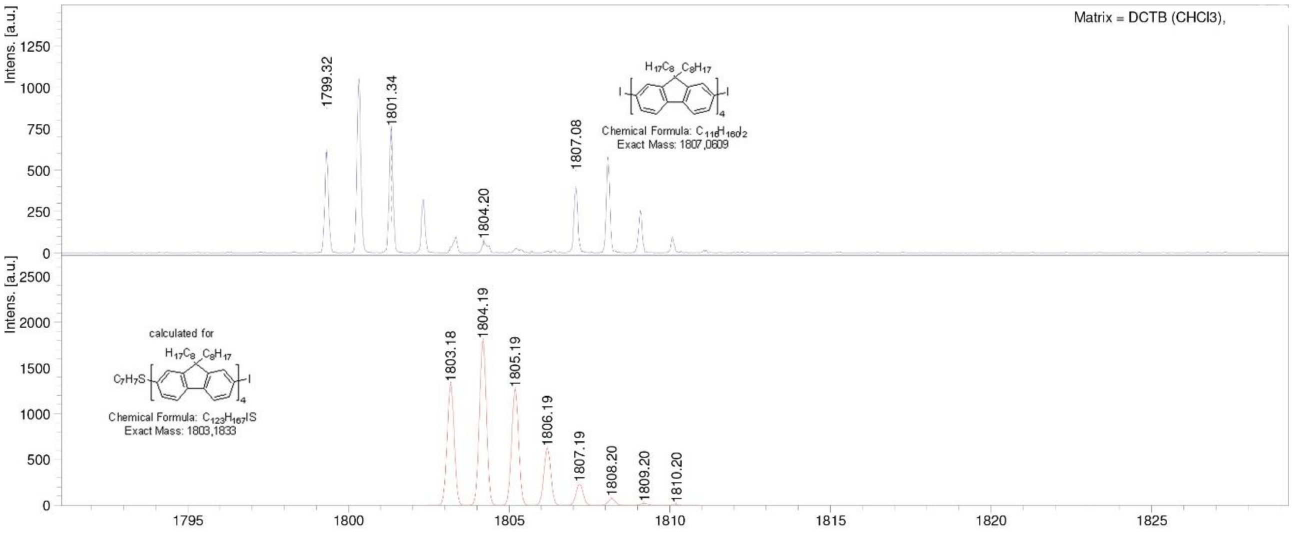
Matrix = DCTB (CHCl3),
Intens. [a.u.]
1799.32
1801.34
1804.20
1807.08
Chemical Formula: $C_{116}H_{160}I_2$
Exact Mass: 1807,0609
calculated for
Chemical Formula: $C_{123}H_{167}IS$
Exact Mass: 1803,1833
1803.18
1804.19
1805.19
1806.19
1807.19
1808.20
1809.20
1810.20

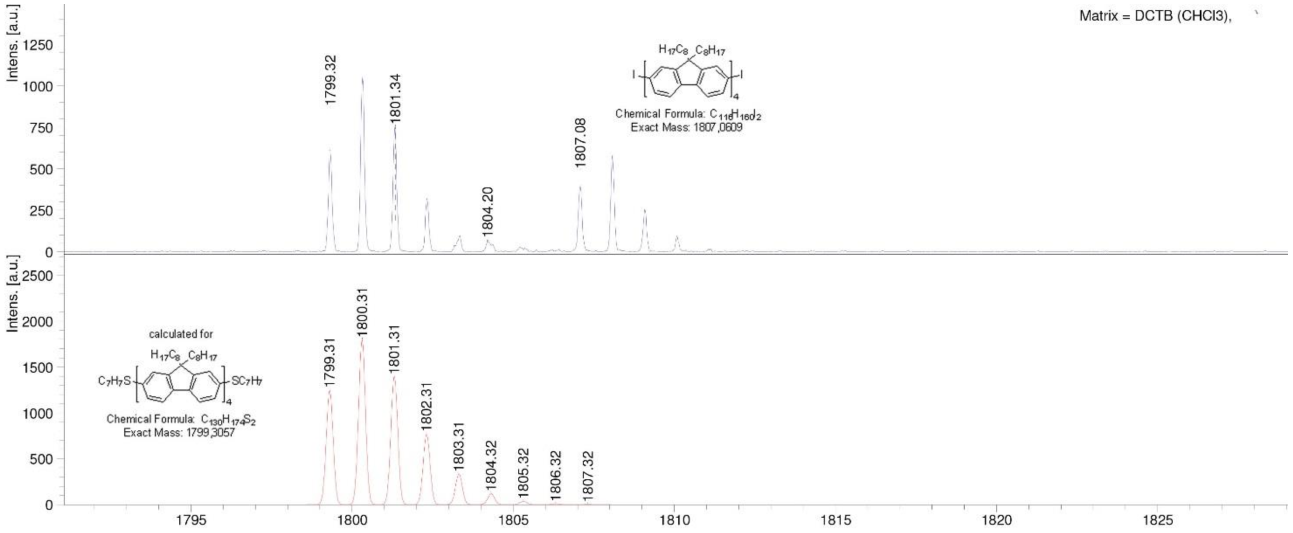
Matrix = DCTB (CHCl3),
Intens. [a.u.]
1799.32
1801.34
1804.20
1807.08
Chemical Formula: $C_{116}H_{160}I_2$
Exact Mass: 1807,0609
calculated for
Chemical Formula: $C_{130}H_{174}S_2$
Exact Mass: 1799,3057
1799.31
1800.31
1801.31
1802.31
1803.31
1804.32
1805.32
1806.32
1807.32

10v

Reaction of **10v** according to general procedure 2. The reaction was stopped after 16 hours and analyzed by MALDI-MS. The product caused by mono substitution (**11v**, m/z = 2191.4963) was not detected.

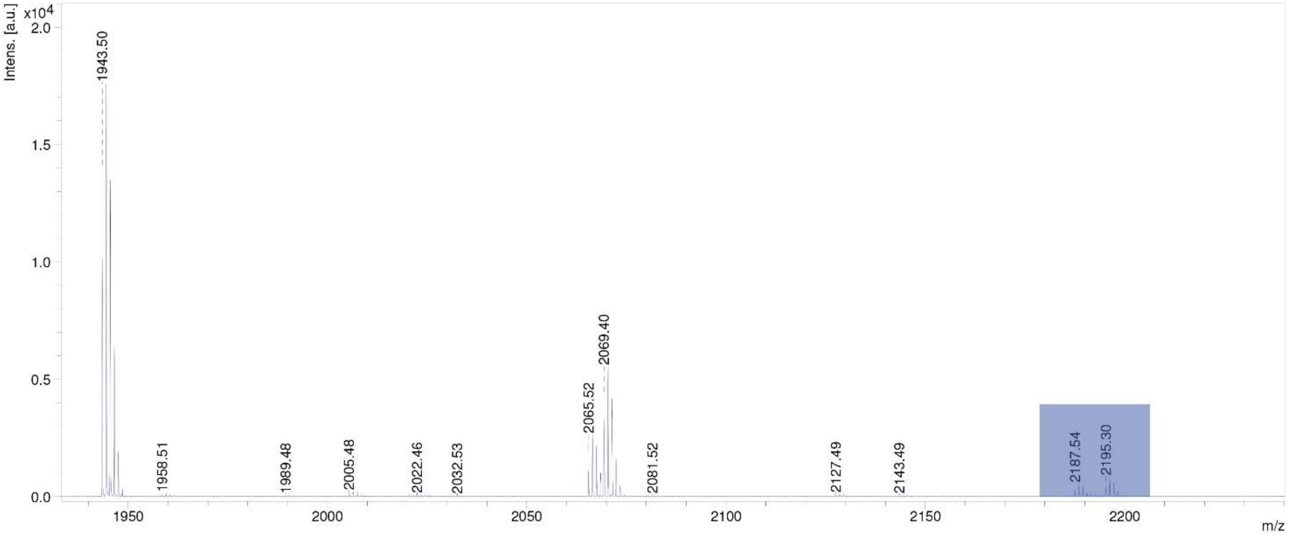

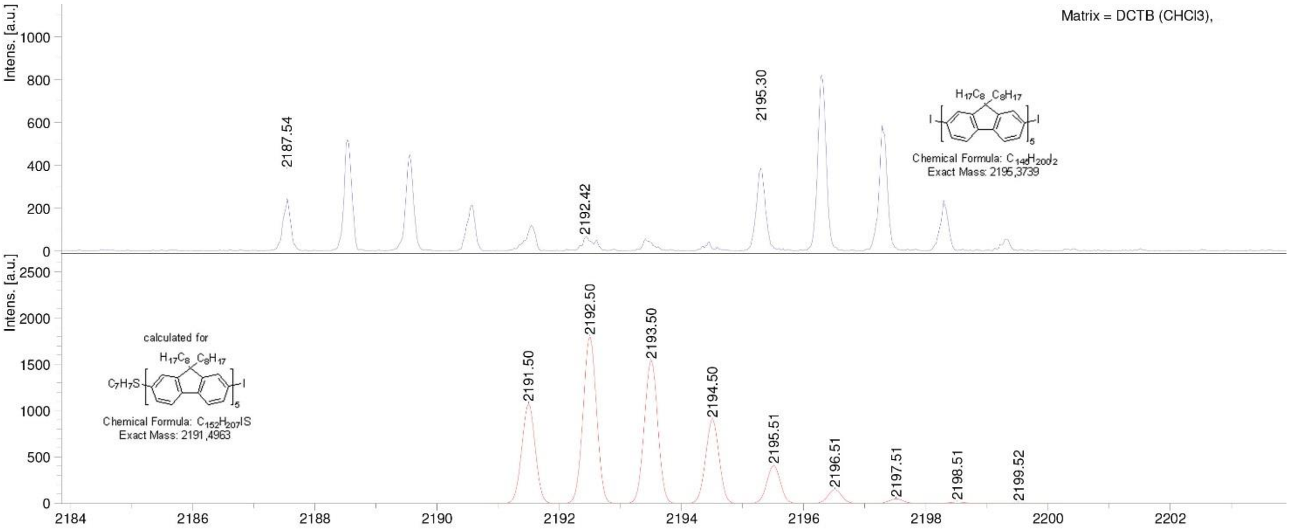

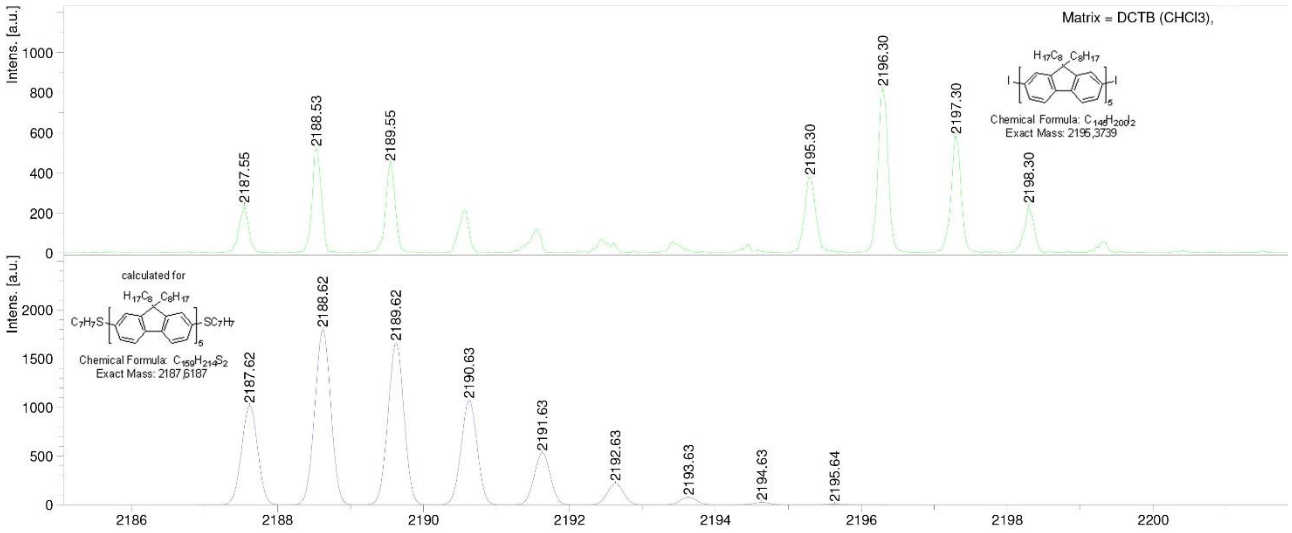

10wa

Reaction of **10wa** according to general procedure 2 using MeCN as a solvent instead of DMSO. The crude reaction mixture was analyzed by MALDI-MS. The open form of the chemical switch (**10wa**) could not be obtained in pure form, about 10% of the material was in the open form (**10wb**). We therefore subjected the mixture of both switches to the substitution reaction. After 6 h only a little mono- and mostly bis substitution could be observed, presumably arising from the 10% of closed form impurity. After 24 h the ratio changed and relative to the bis substitution more mono substitution could be observed (see below). The increase of mono substitution cannot arise from the closed form impurity because experiments with the pure closed form do not show formation of the mono substitution (see below, **10wb**) and therefore have to be a result of the substitution reaction on **10wa**, which seems to be much slower than the reaction of **10wb**.

6 hours

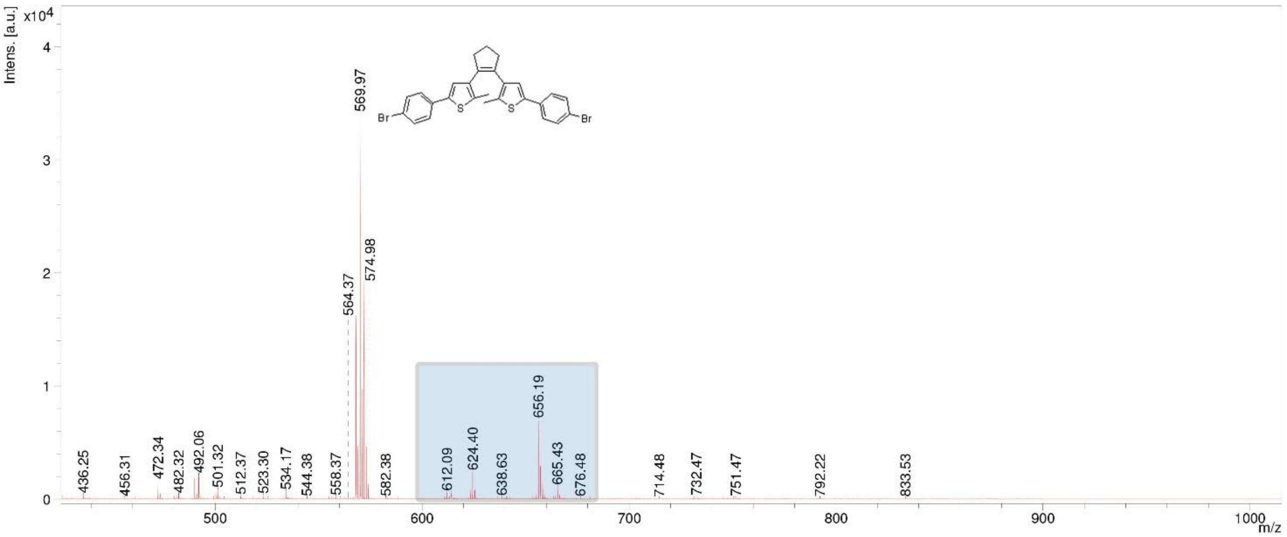
x10^4
Intens. [a.u.]
569.97
574.98
564.37
656.19
624.40
612.09
638.63
665.43
676.48
436.25
456.31
472.34
482.32
492.06
501.32
512.37
523.30
534.17
544.38
558.37
582.38
714.48
732.47
751.47
792.22
833.53
Br
S
m/z

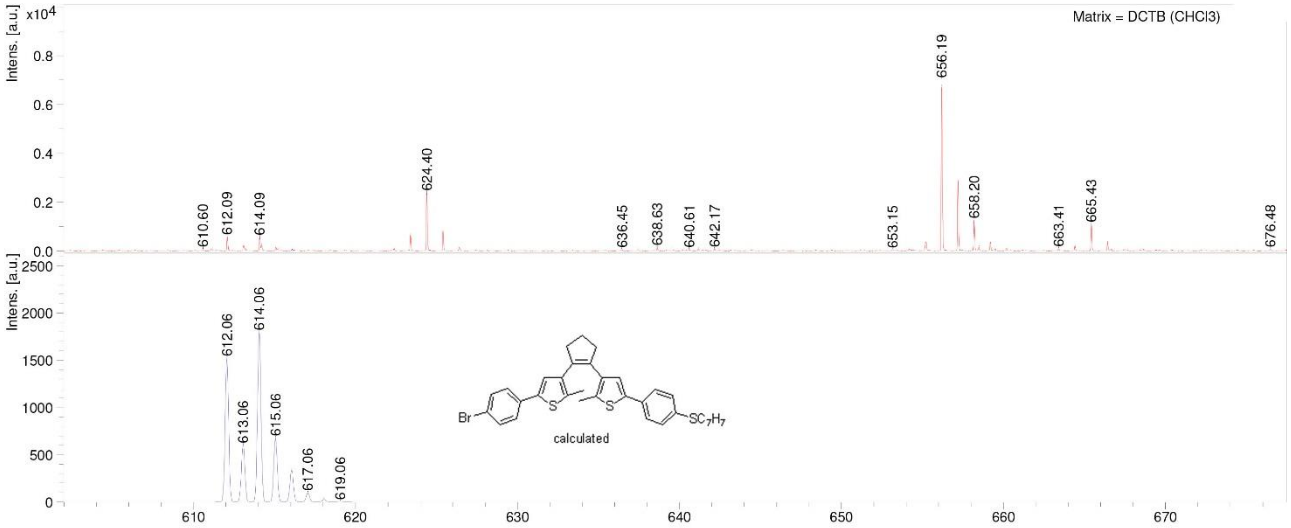
Matrix = DCTB (CHCl3)
x10^4
Intens. [a.u.]
656.19
624.40
658.20
665.43
610.60
612.09
614.09
636.45
638.63
640.61
642.17
653.15
663.41
676.48
612.06
614.06
613.06
615.06
617.06
619.06
Br
S
SC7H7
calculated

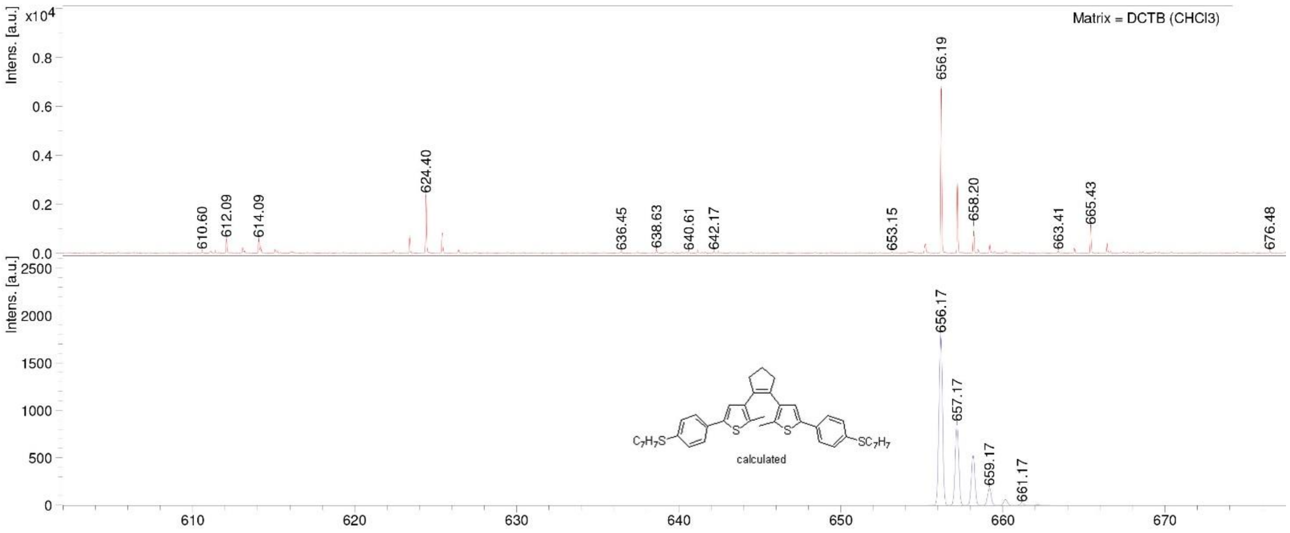

Intens. [a.u.]
x10^4
Matrix = DCTB (CHCl3)
656.19
624.40
658.20
665.43
610.60
612.09
614.09
636.45
638.63
640.61
642.17
653.15
663.41
676.48
656.17
657.17
659.17
661.17
C7H7S
SC7H7
S
S
calculated
m/z

24 hours

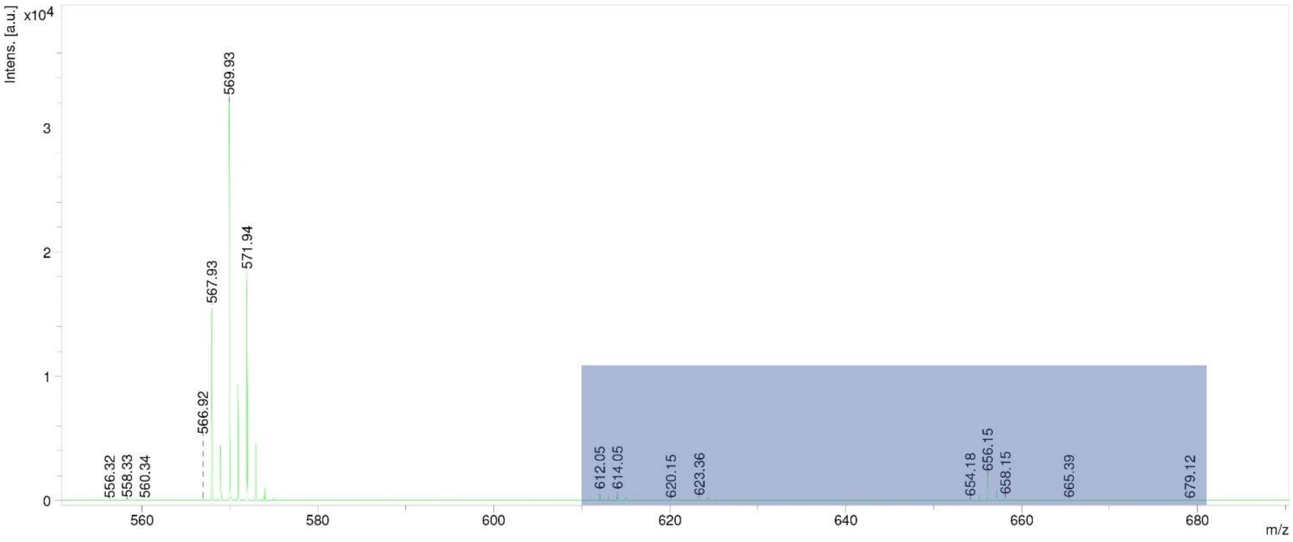

Intens. [a.u.]
x10^4
569.93
571.94
567.93
566.92
556.32
558.33
560.34
612.05
614.05
620.15
623.36
654.18
656.15
658.15
665.39
679.12
m/z

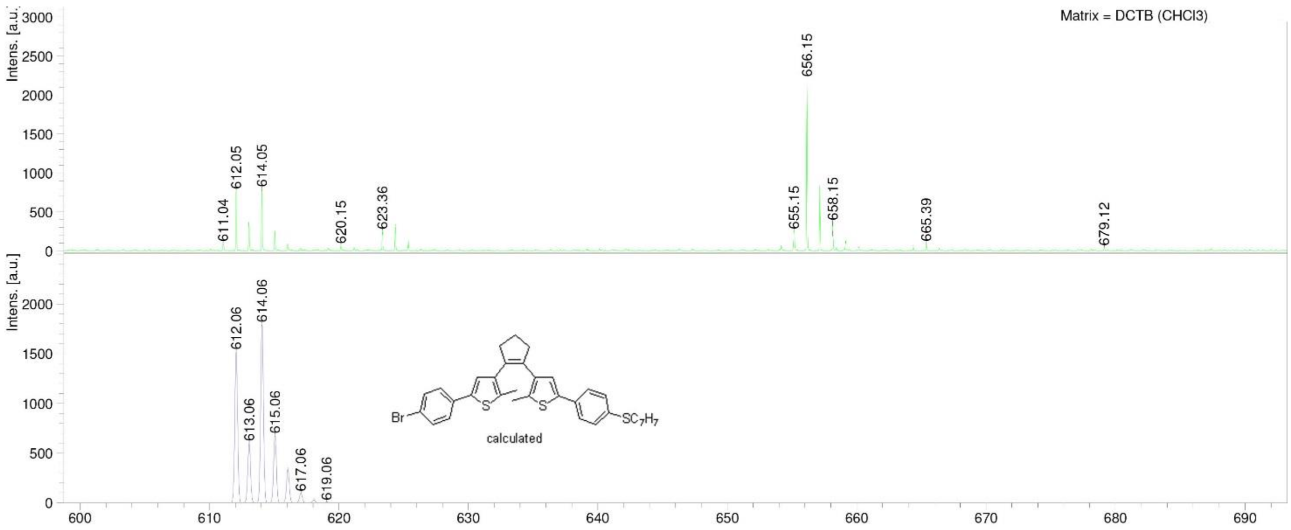

Matrix = DCTB (CHCl3)
Intens. [a.u.]
611.04
612.05
614.05
620.15
623.36
655.15
656.15
658.15
665.39
679.12
612.06
613.06
614.06
615.06
617.06
619.06
Br
S
S
SC7H7
calculated

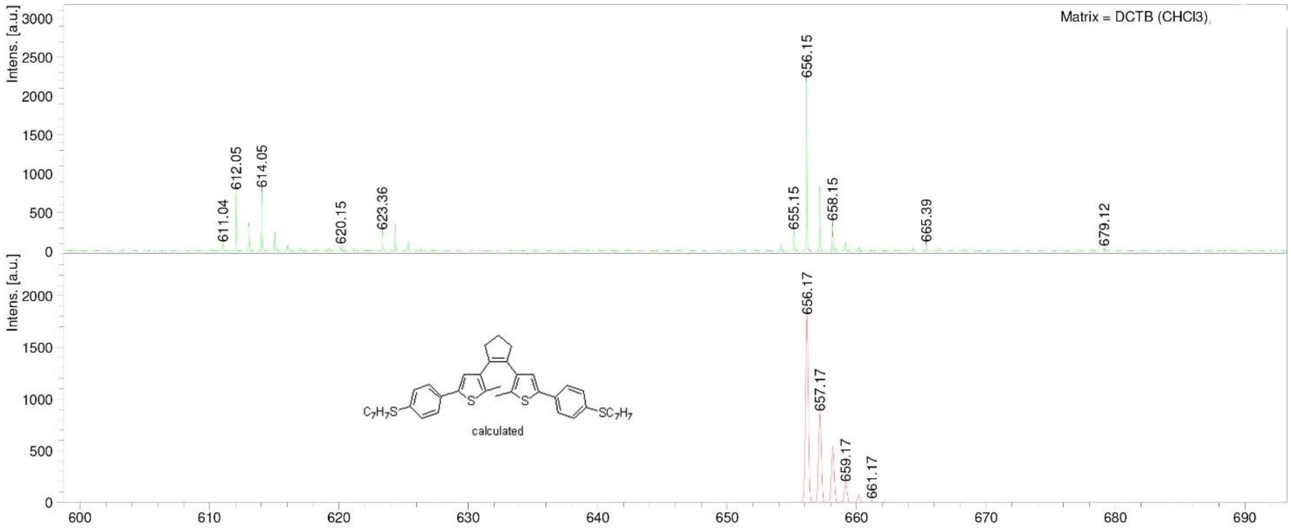

Matrix = DCTB (CHCl3)
Intens. [a.u.]
611.04
612.05
614.05
620.15
623.36
655.15
656.15
658.15
665.39
679.12
656.17
657.17
659.17
661.17
C7H7S
S
S
SC7H7
calculated

10wb

Reaction of **10wb** according to general procedure 1. The reaction was stopped after 2 hours and analyzed by MALDI-MS. The product caused by mono substitution (**11wb**, m/z = 612.0615) could not be observed.

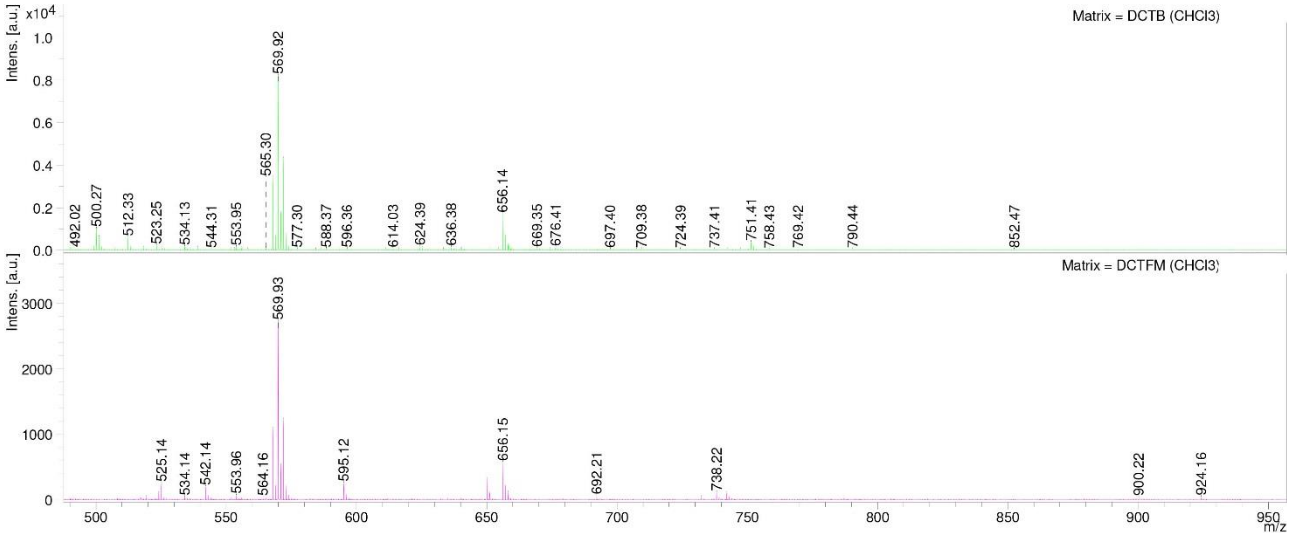

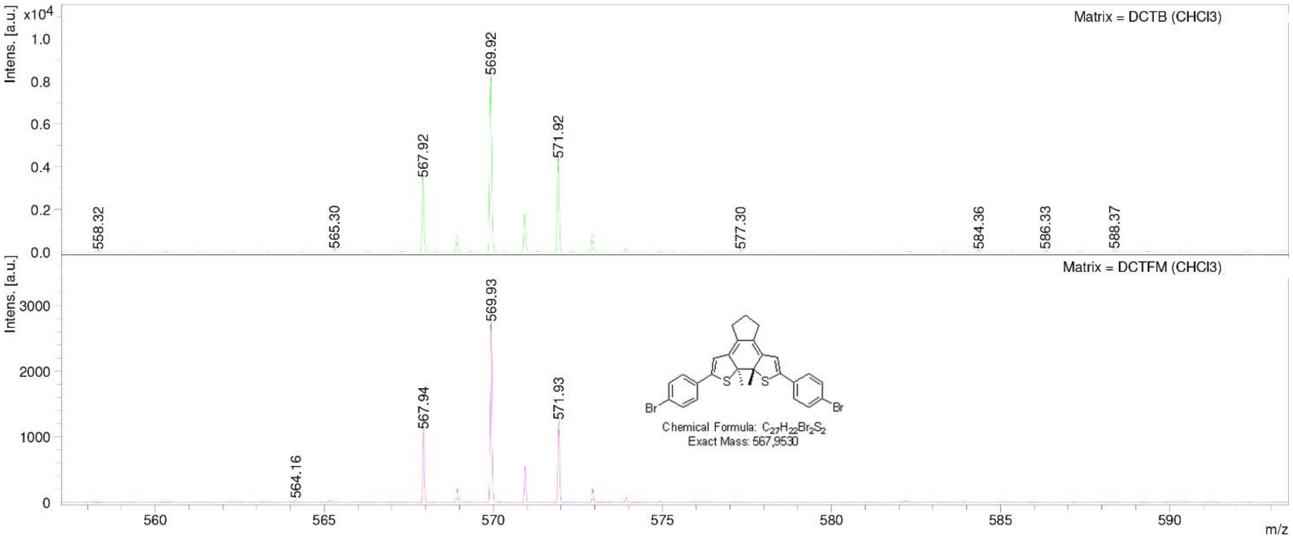

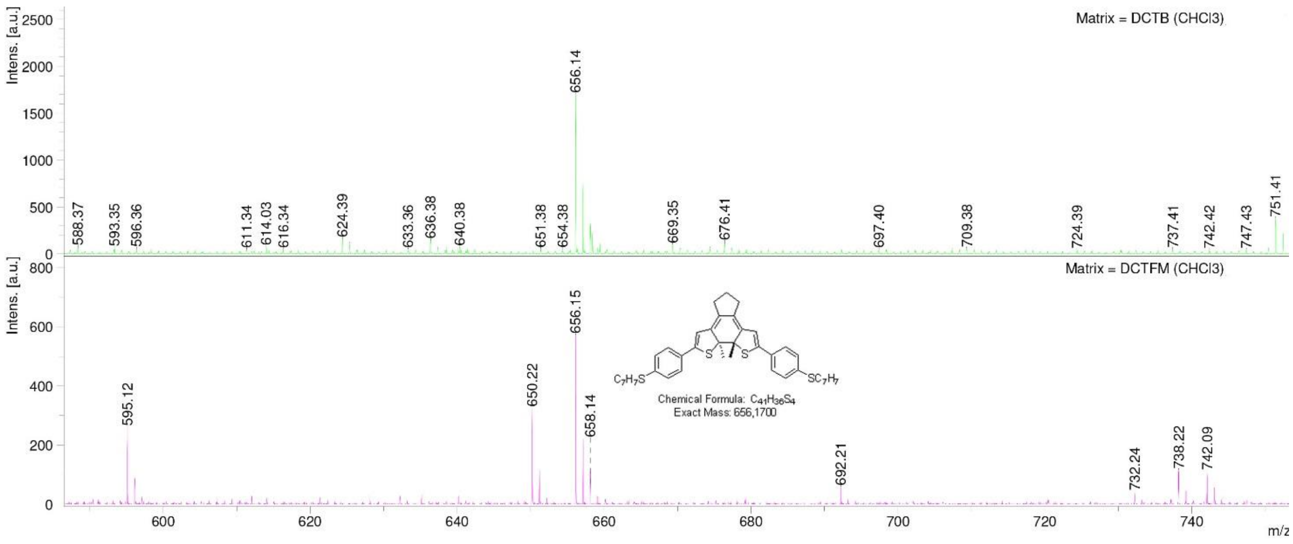
Matrix = DCTB (CHCl3)
Matrix = DCTFM (CHCl3)
Intens. [a.u.]
m/z
C7H7S
SC7H7
Chemical Formula: C41H36S4
Exact Mass: 656,1700

Crystallographic data

Exceptions and special features: Compound **10i** crystallized with two molecules in the asymmetric unit. For both compounds, **10i** and **10j**, one iodine atom was found disordered over two positions. Several restraints (SADI, SAME, ISOR and SIMU) were used in order to improve refinement stability.

X-ray crystal structure analysis of 10i (stu8016, CCDC: 152630)1: formula $C_{16}H_{16}I_2$, *M* = 462.09, colourless crystal, 0.23 x 0.20 x 0.13 mm, *a* = 8.4867(2), *b* = 11.5788(2), *c* = 16.5698(4) Å, *β* = 92.762(4) °, *V* = 1626.4(1) Å^3, ρ_{calc} = 1.887 gcm^{-3}, μ = 3.850 mm^{-1}, empirical absorption correction (0.471 ≤ T ≤ 0.634), *Z* = 4, monoclinic, space group *P*c (No. 7), λ = 0.71073 Å, *T* = 223(2) K, ω and φ scans, 13607 reflections collected (±*h*, ±*k*, ±*l*), 6158 independent (R_{int} = 0.035) and 5928 observed reflections [I>2σ(I)], 344 refined parameters, *R* = 0.033, wR^2 = 0.078, max. (min.) residual electron density 0.46 (-0.90) e.Å^{-3}, hydrogen atoms were calculated and refined as riding atoms.

X-ray crystal structure analysis of 10j (stu8104, CCDC: 1526302): formula $C_{16}H_{14}I_2$, *M* = 460.07, colourless crystal, 0.26 x 0.20 x 0.03 mm, *a* = 11.4988(3), *b* = 15.9491(3), *c* = 8.2449(2) Å, *β* = 97.906(1) °, *V* = 1497.7(1) Å^3, ρ_{calc} = 2.040 gcm^{-3}, μ = 4.180 mm^{-1}, empirical absorption correction (0.409 ≤ T ≤ 0.884), *Z* = 4, monoclinic, space group $P2_1$/c (No. 14), λ = 0.71073 Å, *T* = 223(2) K, ω and φ scans, 11238 reflections collected (±*h*, ±*k*, ±*l*), 3703 independent (R_{int} = 0.046) and 3202 observed reflections [I>2σ(I)], 173 refined parameters, *R* = 0.038, wR^2 = 0.093, max. (min.) residual electron density 0.83 (-0.78) e.Å^{-3}, hydrogen atoms were calculated and refined as riding atoms.

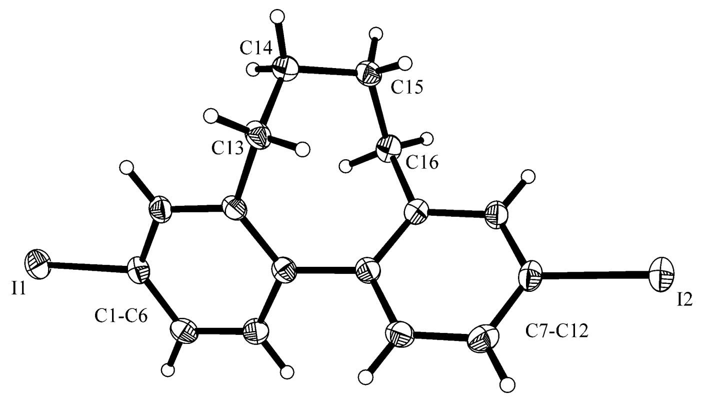

Figure 1. Molecular entity within the crystal structure of compound **10j.**

(Thermal ellipsoids are shown with 30% probability.)

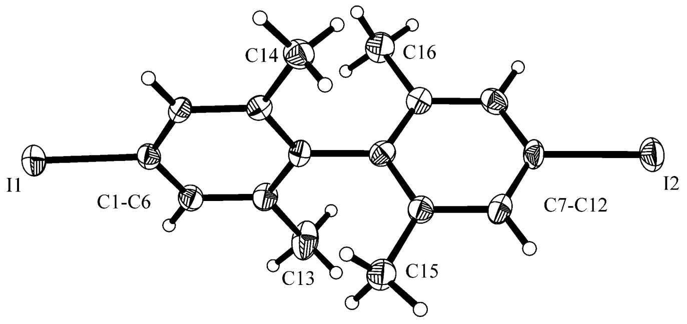

Figure 2. Molecular entity within the crystal structure of compound **10i**.

Only one molecule of two found in the asymmetric unit is shown.

(Thermal ellipsoids are shown with 30% probability.)

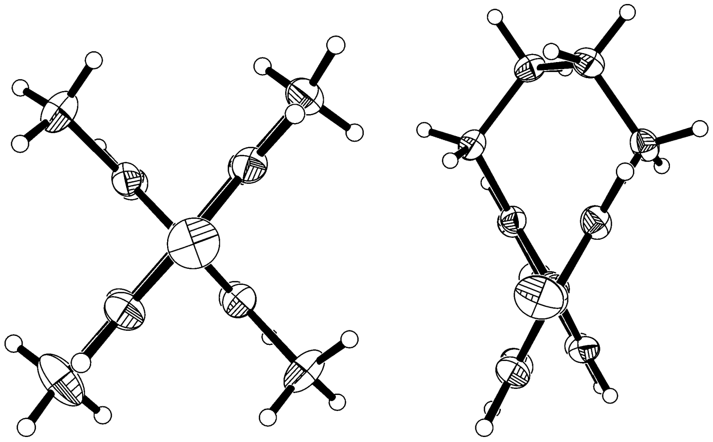

a) 83.0 ° b) 58.1 °

Figure 3. View along the biphenyl system in compounds **10i** (a) and **10j** (b)
